# Synthetic Optimizations for Gram-Scale Preparation of 1-*O*-Methyl d-*Glycero*-α-d-*gluco*-heptoside 7-Phosphate from d-Glucose

**DOI:** 10.3390/molecules27217534

**Published:** 2022-11-03

**Authors:** Konstantin V. Potapov, Roman A. Novikov, Pavel N. Solyev, Sergey N. Kochetkov, Alexander A. Makarov, Vladimir A. Mitkevich

**Affiliations:** 1 Engelhardt Institute of Molecular Biology, Russian Academy of Sciences, 32 Vavilov St., 119991 Moscow, Russia; 2 Zelinsky Institute of Organic Chemistry, Russian Academy of Sciences, 47 Leninsky Avenue, 119991 Moscow, Russia; 3 Biotechnology Department, Sirius University of Science and Technology, 1 Olympic Avenue, 354349 Sirius, Russia

**Keywords:** heptose phosphates, silylation, phosphorylation, bacterial LPS, PAMPs

## Abstract

Heptose phosphates—unique linkers between endotoxic lipid A and *O*-antigen in the bacterial membrane—are pathogen-associated molecular patterns recognized by the receptors of the innate immune system. Understanding the mechanisms of immune system activation is important for the development of therapeutic agents to combat infectious diseases and overcome antibiotic resistance. However, in practice, it is difficult to obtain a substantial amount of heptose phosphates for biological studies due to the narrow scope of the reported synthetic procedures. We have optimized and developed an inexpensive and convenient synthesis for the first performed gram-scale production of 1-*O*-methyl d-*glycero*-α-d-*gluco*-heptoside 7-phosphate from readily available d-glucose. Scaling up to such amounts of the product, we have increased the efficiency of the synthesis and reduced the number of steps of the classical route through the direct phosphorylation of the *O*^6^,*O*^7^-unprotected heptose. The refined method could be of practical value for further biological screening of heptose phosphate derivatives.

## 1. Introduction

**Figure 1:**
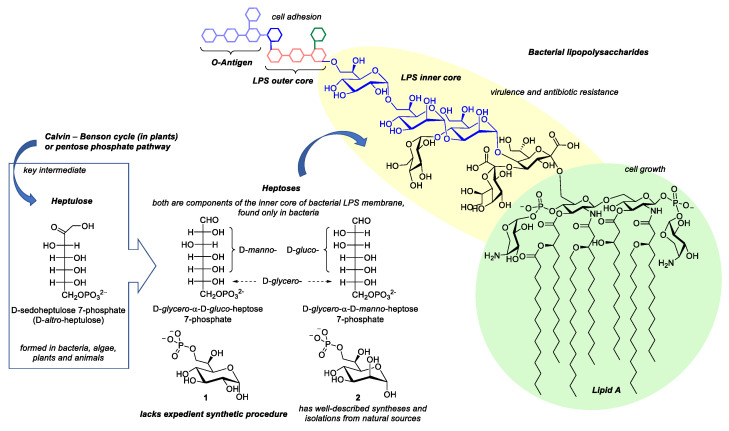
d-glycero-α-d-gluco-heptose and d-glycero-α-d-manno-heptose origin and utilization in the bacterial membrane (generalized LPS structure representation, fatty acids, and heptose epimers may vary in different genera).

Heptoses are seven-carbon (C7) sugars synthesized and utilized exclusively by bacteria [1]. The discovery of natural heptoses prompted chemists to create methods for their total synthesis in the laboratory both to prove their structures and to deduce biosynthetic pathways of their further transformations and products, such as spicamycin, miharamycin and desferrisalmicin. Research on their synthesis received a new round when it became clear that heptoses, in particular d-*manno*-heptose, were present in the phosphorylated forms as a linkage in the lipopolysaccharide (LPS) membrane of gram-negative bacteria, and can also occur as an intermediate in the biosynthesis of 4’-amino-*O*-glycans glycoproteins of gram-positive bacteria [2]. d-Heptopyranose phosphates have recently been recognized as a novel microbial-associated molecular pattern (MAMP) with immunostimulatory potential for cells [3,4]. Such pro-inflammatory activity requires sequential phosphorylation of heptose, for example, d-*glycero*-d-*manno*-heptoside to d-*glycero*-d-*manno*-heptoside 7-phosphate (7-HMP) and d-*glycero*-β-d-*manno*-heptoside 1,7-bisphosphate (β-HBP), activating cellular NF-κB-dependent transcription [5]. The synthetic heptoses may have a potential as antimicrobial agents or artificial vaccines, though the chemical synthesis of heptose phosphates is particularly difficult and inefficient. d-Heptopyranoses are biosynthetically derived from d-sedoheptulose 7-phosphate (d-*altro*-heptulose 7-phosphate) as a key product of the pentose phosphate pathway (Figure 1). In contrast to the bacterial membrane synthesis, d-sedoheptulose 7-phosphate in the Calvin-Benson cycle is further rearranged with d-glyceraldehyde-3-phosphate by the transaldolase, and in some organisms there are more complex transformations into biologically active pseudosugars (derivatives of valiolone) under the action of dehydroquinate synthase (DHQS) family and the related sugar phosphate cyclase enzymes [6]. Extraction from the bacterial membrane, purification, subsequent hydrolysis of LPS, and separation into its constituent components allows milligram amounts of heptoses to be obtained [7]. A vast majority of works in the field of chemical synthesis were previously devoted to the study of *manno*-heptoses (in particular, 7-HMP, **2**)—the main components of the central oligosaccharide LPS of the majority of gram-negative bacteria that link endotoxic lipid A and *O*-antigen together [8,9,10,11].

However, *gluco*-heptoses play their own part in the structural diversity of C7 sugars. l-*Glycero*-β-l-*gluco*-heptose (GGH, the 2’-epimer of d-*manno*-heptose) is a precursor of destomic and epidestomic acids, which are components of aminoglycoside antibiotics hygromycin B and destomycins as well as septacidin, spicamycin, and anisemycin [12]. The last three compounds showed excellent antitumor properties, and septacidin showed itself as an immunogenic inducer of cell death and demonstrated antifungal activity. A semi-synthetic KRN5500 is currently in clinical trials as an anticancer drug candidate and has shown an unexpected bonus side effect: it provides neuropathic pain relief in patients and, additionally, it has been tested as an analgesic in Phase II clinical trials [13].

The sixth-position isomer of GGH, d-*glycero*-l-*gluco*-heptose, is described as a constituent of the peptidoglycan of *Campylobacter jejuni*, the main bacterium that causes toxic food poisoning [14].

d-*Glycero*-d-*gluco*-heptose **1** is scarcely described in terms of biological activity and synthesis. It can be obtained from the extract of primrose roots (yielding 200–250 mg from 20 kg of raw material), where it is a minor C7 sugar, compared with d-*manno*-heptulose and its analogous heptulol—volemitol [15]. A way to obtain d-*glycero*-d-*gluco*-heptosides was described in the total synthesis of desferrisalmicin B [16]. A more concise synthesis of 1-*O*-methyl d-*glycero*-d-*gluco*-heptoside derivatives as potent GmhA isomerase and HldE kinase inhibitors in bacterial regulation was proposed by Vincent and colleagues [17]. An alternative route of synthesis was described in the paper by the Aspinall’s group, who used TMSthiazole homologation reaction to form 1-methyl 2,3,4-tri-O-benzyl-d-glycero-α-d-gluco-heptopranoside in 1.1 g amount (as the Bn-protected derivative) in good yields, however, using older mercury salt-based protocols that are considered to be toxic [18].

d-*Glycero*-α-d-*gluco*-heptose is of interest as an understudied sugar with biological activity. The development and application of an effective method for synthesis of its derivatives in gram amounts is of great practical value, especially for further biological testing. In this paper we describe the optimization of Vincent’s group’s synthesis pathway to a gram-scale quantity of 1-*O*-methyl d-*glycero*-α-d-*gluco*-heptoside 7-phosphate.

## 2. Results and Discussion

### 2.1. General Synthetic Route

**Scheme 1:**
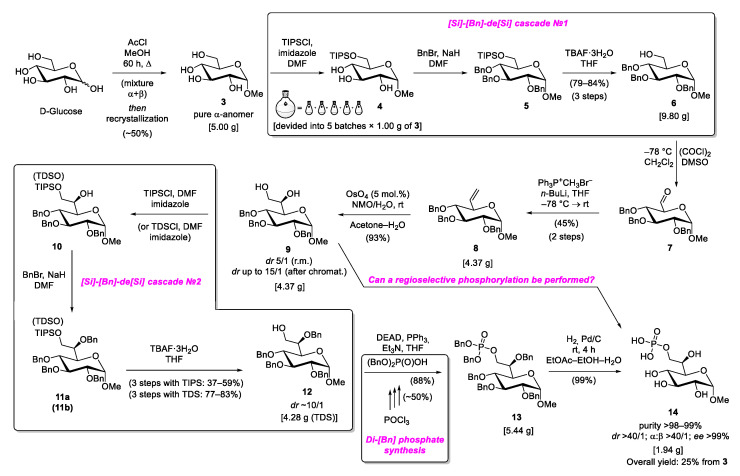
Synthetic route from d-glucose to methylated d-*glycero*-α-d-*gluco*-heptoside based on Vincent’s group route. Average yields of substances to reproduce synthetic scheme are shown in square brackets. Optimized procedures for upscale synthesis are highlighted in frames.

Currently, there are two modern routes for the synthesis of d-*glycero*-d-*gluco*-heptoside core: (1) from d-xylose—the sugar chain is extended from C5 to C7 using the Horner–Wadsworth–Emmons (HWE) reaction with (EtO)_2_P(O)–CH_2_–CO_2_Me (described for d-*glycero*-d-*gluco*-heptoside [19], but total synthesis from commercial reagents was reported only for the d-*manno*- analogue [20]), followed by a pentose-furanose rearrangement at the final step of deblocking the protective groups, and (2) from more easily accessible d-glucose without rearrangement. Despite the seemingly insignificant changes in the skeleton of the final product, both routes require a large number of stages and reduce the overall yield of the final product to several percents. The first option can reduce the number of stages and increase the efficiency of synthesis, but it is more risky and much more difficult to implement. Due to the availability of d-glucose and a great potential for optimizing synthesis conditions, we have chosen a 12-step synthesis proposed in the second route, which is more reliable (Scheme 1). The key extension of the sugar carbon chain from C6 to C7 is performed by the sixth step of the Wittig reaction using Ph_3_P=CH_2_ after all the necessary protective groups have been introduced, followed by the stereoselective hydroxylation with osmium tetroxide OsO_4_.

It should be noted that this synthetic sequence was first concisely described for several tens of mg of final heptoside **14** (32 mg in the article [15]) and was probably the only work describing the complete synthesis of d-*glycero*-d-*gluco*-heptose—all other studies mainly focused on derivatives of *glycero*-d-*manno*-heptose [11,17,21,22]. In addition, we also considered alternative synthetic routes for adaptation, those that were not revealed for d-*gluco*-heptose synthesis in the literature. To date, there are several new papers on d-*glycero*-d-*manno*-heptose synthesis with more sophisticated procedures, which can be modified to build d-*glycero*-d-*gluco*-heptoses [14,23].

Scrutinizing the literature, our main goal was the optimization of the Vincent et al. protocol for gram quantities of the final 1-methylated inhibitor while adopting new step-economy routes. The concise synthetic route is discussed below, and particular findings highlighted in frames in Scheme 1 will be described in separate paragraphs afterwards.

In our case, the synthesis of 2,3,4-tribenzylated glucose **6** was achieved in a 4-step protection/deprotection sequence on the basis of the described methods [17] and the protecting group’s performance was assessed. At the first step, d-glucose was methylated and the required pure α-anomer **3** was further crystallized. Dynamic resolution under thermodynamic control could gain more target anomer **3** by the repeated crystallization from the acidic mother liquor if needed, however, it was unnecessary with such a common and cheap starting material. Then, silylation at the 6-OH group, benzylation at the remaining three 2,3,4-OH groups, and removal of the silyl protection was carried out to obtain the target compound **6**, with a cumulative yield after optimization up to 84% in three steps out from **3**. The isolation of individual isomers could be carried out by obtaining the fully protected compound **5**, though not on previous steps. Further, the CH_2_OH group in 2,3,4-tribenzylated glucose **6** was oxidized to aldehyde **7** by the Swern oxidation with oxalyl chloride and DMSO, and subsequently converted the aldehyde to the vinyl group using the Wittig reaction, extending the carbon chain to the precursor of a C7-sugar (**8**), with the yield being 45% in two steps. Our next step was the well-established catalytic Upjohn hydroxylation of the formed double C=C bond with OsO_4_ (5 mol. %), carried out in the high yield (93%). The reaction outcome was in accordance with the Kishi empirical rule [24]: the major product of osmylation had the newly introduced adjacent hydroxy group *anti* to the resident alkoxy (or hydroxy) stereocenter. The stereoselectivity of hydroxylation into compound **9** was ~5/1 (d-*glycero* / l-*glycero*), which was consistent with the previous papers on the synthesis of the *manno*-heptose analogue [11]. The hydroxylation did not require further improvement, since after necessary chromatographical isolation procedures the *dr* automatically increased to >15/1. At the same time, the minor l-*glycero* isomer can also be isolated and used, if necessary. Next, the selective benzyl protection of the acyclic 6-OH group in sugar **9** was carried out in three steps using the sequence of silylation/benzylation/[Si]-removal. This cascade required significant optimization of synthetic procedures to obtain 2,3,4,6-tetrabenzylated d-*glycero*-d-*gluco*-heptoside **12**, that will be discussed below. At the last step, heptose **12** was phosphorylated at the 7-CH_2_OH group using the Mitsunobu reaction with dibenzyl phosphate. Finally, deprotection of all six benzyl groups in **13** by hydrogenolysis with hydrogen on a Pd/C catalyst in aqueous ethanol proceeded in a quantitative yield, giving a solution of the pure target 1-methylated d-*glycero*-d-*gluco*-heptoside 7-phosphate **14**, which was isolated as white powder by freeze-drying. The purity of the final **14** is >98.5%, the ratio of d-*glycero*/l-*glycero* diastereomers is >40/1, α/β anomers ratio is >40/1, and the product is optically pure (d-isomer, *ee* >99%).

The optimized scheme for the synthesis of 1-*O*-methyl d-*glycero*-d-*gluco*-heptoside 7-phosphate **14** proved to be quite efficient in terms of atom-economy with the overall yield of 25.3% over 11 steps (gram-scale), based on the commercially available cheap 1-*O*-methyl α-d-*gluco*-pyranoside **3** (<0.3–0.5 $/1 g) [25]. An overall yield can be further increased after optimization of the Wittig olefination. In addition, the synthesis scheme turned out to be perfectly adapted to produce significant gram-scale amounts of **14**. Thus, to obtain 1.94 g of the target compound **14**, we had to use only 5.00 g of the 1-*O*-methyl α-d-*gluco*-pyranoside **3**. Obtaining a few grams of heptoside **14** does not pose any problem either.

### 2.2. Structure Elucidation of the Sugar Configuration

**Figure 2:**
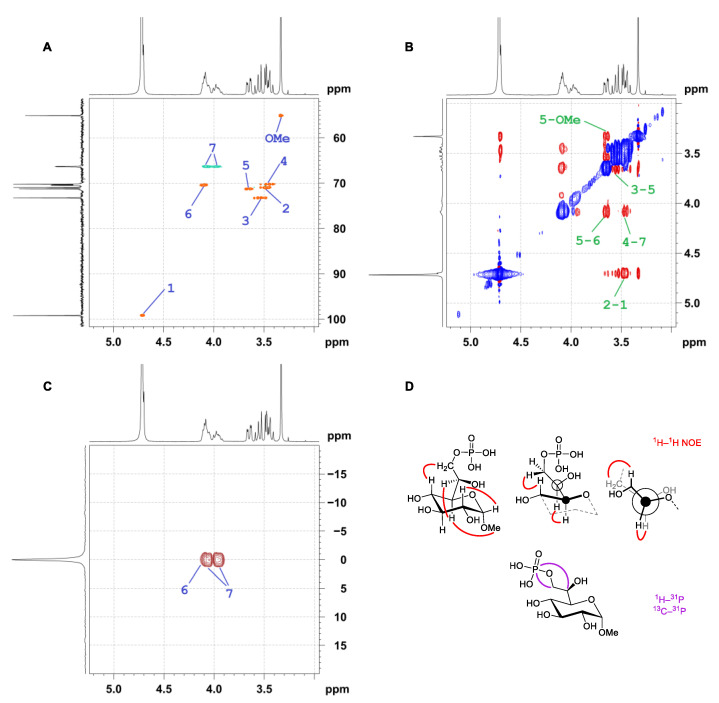
The representation of NMR spectra regions in D_2_O used for assignments: (**A**) 2D ^1^H–^13^C edited-HSQC, (**B**) ^1^H–^1^H NOESY and (**C**) ^1^H–^31^P HMBC. (**D**) Accurate configuration assignment of the final heptoside **14** (including acyclic stereocenter) using ^1^H–^1^H NOESY and ^1^H–^31^P HMBC spectra.

The structure of the resulting 1-methyl d-*glycero*-d-*gluco*-heptoside 7-phosphate **14** was carefully studied and confirmed by NMR spectroscopy (^1^H, ^13^C, ^31^P, 2D COSY, NOESY, edited-HSQC, HSQC-TOCSY, HMBC, ^31^P HMBC) (see Supplementary Materials), including consistency with the previous literature data [15]. Special emphasis was made on verification of the stereochemistry of the molecule. The ^1^H–^31^P and ^13^C–^31^P spin-spin coupling constants, including 2D ^1^H–^31^P HMBC experiments, firmly support the outcome of heptose **1** phosphorylation at position 7 (Figure 2). Analysis of the stereochemistry of the molecule using 2D ^1^H–^1^H NOESY NMR experiments (in D_2_O, optimal mixing time of 350 msec) verifies d-*glycero* and α-d-*gluco* configurations (configuration analysis of the acyclic stereocenter was performed in Newman projections, Figure 2D).

### 2.3. Silylation Group Challenges and Optimizations

**Scheme 2:**
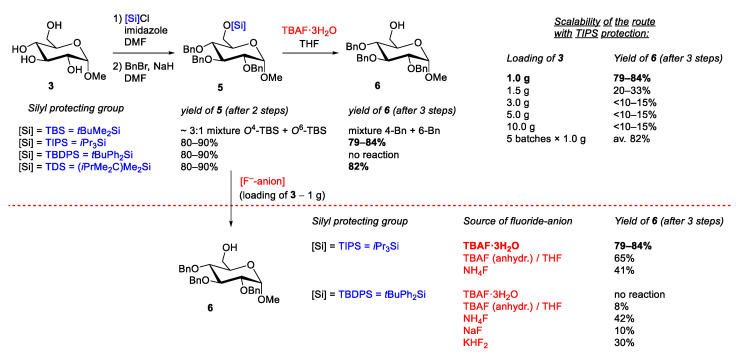
Optimization of the protection/deprotection steps for gram-scale quantities.

We applied some significant changes in synthetic procedures when upscaling to amounts greater than 10 g. Attention has been paid to several separate issues and steps that required our thorough analysis and development in relation to greater amounts of the substances. First, silylation/benzylation/desilylation sequence to transform 1-methyl α-d-glucopyranoside **3** into 2,3,4-benzylated sugar **6** appeared to be the most troublesome. As those were the initial stages of the complete synthesis, they required reagent loading of larger quantities (≥ 5 g), leading to the scalability issues (Scheme 2). It turned out that *tert*-butyldimethylsilyl protection (TBS) was inapplicable for the route, since the silyl group migration occurred from the 6-*O* position to the 4th position hydroxy group under the reaction conditions of the subsequent benzylation step. The most sterically bulky *tert*-butyldiphenylsilyl group (TBDPS) worked well but caused significant problems in the silyl deprotection step (details on that issue below). From that point of view, a balance had to be found between these two extremes, and the use of triisopropylsilyl (TIPS) or thexyldimethylsilyl (TDS, (*i*PrMe_2_C)Me_2_Si) protecting groups resulted in high yields of the target product **6**. We ended up using a more common TIPS protecting group.

The second challenge was determined by the unusual feature of that three-step synthesis (compound **3** → **6**) in its upscaling to more than 1 g of 1-*O*-methyl α-d-*gluco*-pyranoside **3** loading. Quite surprisingly, when the reagent loading had passed from 1 g limit to only 1.5 g, the yield of compound **6** (based on 3 steps) dropped from 79–84% to less than 33%, and further to 10–15% when upscaling to 2.0 g and more. That drop-in was firmly reproduced in a dozen replicate syntheses with the chemicals from different supplier batches. This was also observed with a further increase in the starting quantities of compound **3** to 3 g, 5 g, and 10 g. We did not succeed to understand this phenomenon, but it may be referred to the specialists in physical effects in chemical kinetics [26]. Since we aimed to upscale the synthesis to more than 10 g, we had to settle for running several reaction batches in a parallel setting with 1 g of **3** each.

Finally, the third deprotection step to **6** was additionally optimized on a gram-scale amount, and different fluoride-anion sources were tested (Scheme 2). The most selective but least active was TBAF trihydrate. It produced the highest yields of the product **6** in the case of TIPS and TDS deprotection. However, TBAF · 3H_2_O did not work well for TBDPS deprotection (a cheaper alternative for [Si]-protective group), which required more active fluoride sources such as anhydrous TBAF, NaF, NH_4_F, or KHF_2_. The last two fluorides were effective for TBDPS deprotection, but the yield of product **6** dropped significantly after purification. For TIPS deprotection, inorganic fluorides appeared to be too active; thus, the selectivity and yields were also reduced.

**Scheme 3:**
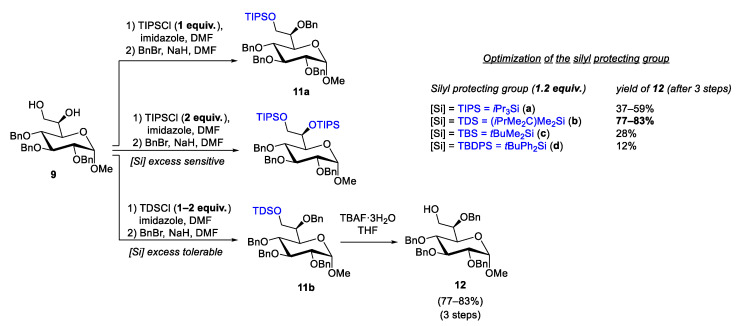
Optimization of the late-stage protection/deprotection steps.

Another block of silylation/benzylation/desilylation sequence in the synthesis route (compound **9** to compound **12**) caused major problems at the silylation step. Moreover, the reported technique [15] poorly reproduced the yields, if proceeded at all. The step required selective silylation at the 7-OH group. However, the use of TIPSCl in a slight molar excess led straight to the facilitated silylation at the acyclic 6-OH group, with the formation of the disilyl derivative (Scheme 3). The following benzylation/desilylation of the di-TIPS-protected derivative resulted in the starting sugar **9**. The selectivity problem of the silylation was partially resolved using a strict stoichiometric ratio of reagents **9**: TIPSCl as 1:1 (instead of a typical excess of silyl chloride in classical [Si] procedures), though the yield of the final product **12** was still low (37–59%). To solve this problem and to increase the yield, we performed a number of optimization experiments to replace TIPS protecting group with a more convenient silyl analogue (Scheme 3). The best solution was found when using the specific TDS protecting group, which possesses an optimal balance between its steric volume and the reactivity for the sugar substrate. TDS almost doubled the yield of **12**, up to 83%. At the same time, other common protecting groups TBS and TBDPS gave low yields.

### 2.4. Phosphorylation Using Dibenzyl Phosphate

**Scheme 4:**
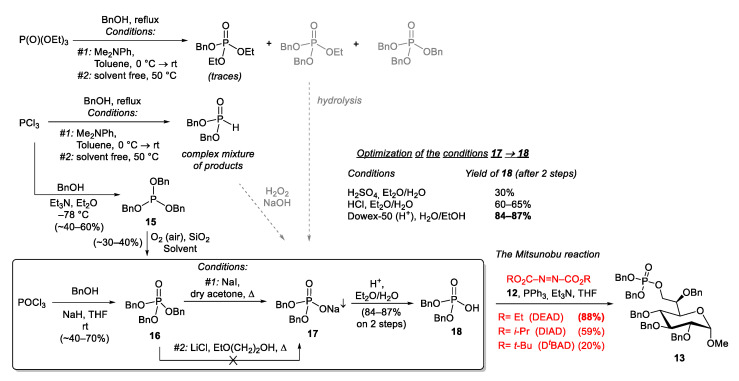
Synthesis of dibenzyl phosphate.

The final junction was originally performed by the Mitsunobu phosphorylation. It required benzyl-protected phosphate as a reagent, which, although commercially available, is expensive and rather rare, and shortly shelf-stable. Difficulties in procurement and transportation prompted us to conduct the synthesis of dibenzyl phosphate P(O)(OBn)_2_(OH). There are several methods to obtain P(O)(OBn)_2_(OH), most of which are reported in outdated papers [27,28,29,30]. These methods turned out to be rather poorly reproduced in modern practice. Initially, we tried three unsuccessful synthetic schemes based on phosphorus trichloride PCl_3_ and triethyl phosphate P(O)(OEt)_3_ (Scheme 4). Transesterification was inefficient at any conditions, even with strong bases. Selective benzylation of PCl_3_ resulted only in tribenzyl phosphite **15** with moderate yields, which could be further oxidized into phosphate **16** on air while performing column chromatography. However, it was possible to work out a more efficient base-catalysed synthesis of **16** from phosphorus oxychloride. The method is relevant to the reported protocol for synthesis of long-chain dialkyl phosphates, which could be isolated and purified in a current of steam through the heated toluene solution under reflux conditions [31]. In our case with benzyl esters, we had to use full esterification into tribenzyl phosphate, and at the next step one benzyl group was selectively hydrolyzed to form a precipitated sodium salt **17**. This approach appeared to be successful and did not require harsh conditions. The product **17** was filtered off, treated with either hydrochloric or sulfuric acid and extracted into ether solution in the acidic form, giving pure crystalline dibenzyl phosphate P(O)(OBn)_2_(OH) **18** with a total yield of up to 45%. Using Dowex-50 ion-exchange resin (H^+^ form) for the desalination step allowed us to increase the yield of the target dibenzylphosphate **18** to 87% and the total yield of the sequence to 61% (based on POCl_3_).

Finally, the obtained dibenzyl phosphate was involved in the Mitsunobu reaction with the protected heptoside to result 1-*O*-methyl perbenzylated heptoside **13** (Scheme 1) in a reasonable yield for these types of reactions. Hydrogenolysis resulted in the deprotected target product **14** in the overall yield of 25.3% over 11 steps (from 1-*O*-methyl α-d-glucopyranoside **3**).

### 2.5. Phosphorylation Using Dibenzyl Phosphoriodidate

In order to take a shortcut in the synthesis route into phosphate and to omit the second cascade of CH^6^-OH protection steps (**9** → **13** sequence), we tried the direct phosphorylation of **9**. Several known phosphorylating reagents for primary alcohols were considered, and yet, only few of them could be suitable for vicinal diols in semiprotected heptosides. Both sufficient reactivity and exceptionally mild conditions are necessary for regioselectivity towards the primary alcohol.

Phosphoramidites, phosphohalogenates and pyrophosphates with easily cleavable allyl or benzyl substituents were possible candidates for phosphorylating agents. Phosphoramidites can be used for initial phosphitylation and react with primary alcohols, with subsequent oxidation into phosphates by peroxides, in good yields (~65%) [32,33]. The limiting point is that dibenzyl phosphoramidite is expensive and still requires dibenzyl chlorophosphite to be synthesized first. It could not be obtained easily and in good yield. Dibenzyl chlorophosphite is also a common starting reagent to obtain anhydrides (pyrophosphate esters), which is why they were not tested as well.

A number of attempts were reported previously for the synthesis of dibenzyl phosphodichloridate via the direct chlorination [34]. According to Vincent’s group’s paper, sulfuryl chloride, trichloroisocyanuric acid, and carbon tetrachloride used for diallyl product never exceeded 50% yield, and dibenzyl phosphodichloridate posed even more problems in by-product formation [34]. The most accurate synthesis of dibenzyl phosphodichloridate required iodination and hydrolysis into phosphate and its chlorination with oxalyl chloride in the presence of DMF at room temperature to give approximately 81% yield [34]. We managed to find one example of heptose phosphorylation distinguishing primary and secondary hydroxyl by diphenyl phosphodichloridate and phosphoramidite (BnO)_2_PN(*i*Pr)_2_ [32].

**Scheme 5:**
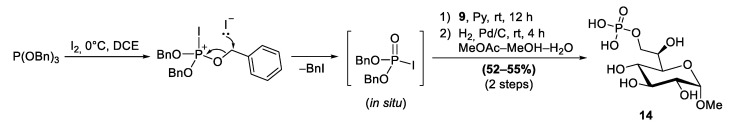
The direct phosphorylation of the primary hydroxy group via dibenzyl phosphoriodidate.

An oxidation promoted by iodine prompted us to use iodination for the direct synthesis of phosphoriodidate esters and apply them straightforward for phosphorylation, in order to circumvent several manipulations of purification (Scheme 5). It should be noted, that dibenzyl phosphoroiodidate synthesis was described a couple of times as a simple and non-time-consuming route for phosphorylation [35,36]. We applied this method, which proved to be appropriate for half-gram quantities of the sugar **9**, when performed in pyridine, and the hydrogenation of the [Bn]-groups was conducted in “one-pot.” Yields over these two steps were between 52 and 55% (independently several times). Considering the fact that it reduces three steps of synthesis with 34–73% yields (depending on the batch) and omits the necessity of intermediate chlorination/amidation, we can conclude that this is a handy protocol for the main route of **14** synthesis. The reagent (BnO)_2_PO-I can be synthesized in situ on demand and the whole method is easy to handle.

## 3. Conclusions

We have presented an optimized and convenient gram-scale synthesis of 1-*O*-methyl d-*glycero*-α-d-*gluco*-heptoside 7-phosphate—a prospective derivative of the understudied pathogen-associated molecular pattern. We have benchmarked protective groups in two different cascades and checked their suitable deprotection conditions and, as a result, we have reached high yields in the C6 → C7 sugar transformation. Several methods of dibenzyl phosphate building block linkage have been discussed and tested for the final steps of phosphorylation. Another important implication for step economy has been introduced by the direct phosphorylation of the *O*^6^, *O*^7^-unprotected heptose. The developed method allows synthesis of 1-*O*-methyl d-*glycero*-α-d-*gluco*-heptoside 7-phosphate in considerable amounts for biological studies. These findings may be further used for the synthesis of various heptose phosphates. Broadening the representative library of heptose compounds is expected in due course.

## Data Availability

Not applicable.

## Supplementary Materials

The following supporting information can be downloaded at: https://www.mdpi.com/article/10.3390/molecules27217534/s1, Pages S3–S11: Experimental procedures for synthesis and compounds characterization (**3**–**14**); pages S12–S52.: ^1^H, ^13^C, ^31^P and 2D NMR spectral data for compounds.
