# Supplementary material for: Synthetic Optimizations for Gram-Scale Preparation of 1-O-Methyl d-Glycero-α-d-gluco-heptoside 7-Phosphate from d-Glucose
Source: Molecules. 2022 Nov 3;27(21):7534. doi: 10.3390/molecules27217534 (PMC9654166; doi:10.3390/molecules27217534)

ELECTRONIC SUPPLEMENTARY MATERIAL

Synthetic Optimizations for Gram-Scale Preparation of 1-*O*-Methyl D-*Glycero*-α-D-*gluco*-heptoside 7-Phosphate from D-Glucose

Konstantin V. Potapov [1,2,†], Roman A. Novikov [1,2,3,†], Pavel N. Solyev [1,*,†], Sergey N. Kochetkov [1], Alexander A. Makarov [1] and Vladimir A. Mitkevich [1]

[1] Engelhardt Institute of Molecular Biology of the Russian Academy of Sciences, 32 Vavilov St., 119991 Moscow, Russia

[2] Zelinsky Institute of Organic Chemistry of the Russian Academy of Sciences, 47 Leninsky Avenue, 119991 Moscow, Russia

[3] Biotechnology Department, Sirius University of Science and Technology, 1 Olympic Avenue, 354349, Sirius, Russia

* Correspondence: solyev@gmail.com; Tel.: +7-499-135-60-65

† These authors contributed equally to this work.

Experimental section

Reagents and equipment -- S3
Procedures for synthesis and compounds characterization --------------------------------- S3
1-*O*-Methyl α-D-*gluco*-pyranoside (**3**) --- S3
1-*O*-Methyl 2,3,4-tri-O-benzyl-α-D-*gluco*-pyranoside (**6**) --------------------------------------- S4
1-*O*-Methyl 2,3,4-tri-*O*-benzyl-6-deoxy-α-D-*gluco*-hept-6-enopyranoside (**8**) --------------- S5
1-*O*-Methyl 2,3,4-tri-*O*-benzyl-D/L-*glycero*-α-D-*gluco*-heptopyranoside (**9**) ----------------- S6
1-*O*-Methyl 2,3,4,6-tetra-*O*-benzyl-D-*glycero*-α-D-*gluco*-heptopyranoside (**12**) ------------- S7
Tribenzyl phosphate (16) -- S8
Dibenzyl phosphate, sodium salt (**17**)-- S8
Dibenzyl phosphate (**18**) -- S8
1-*O*-Methyl 2,3,4,6-tetra-*O*-benzyl-D-*glycero*-α-D-*gluco*-heptopyranoside 7-(dibenzyl)phosphate (**13**) -- S9
1-*O*-Methyl D-*glycero*-α-D-*gluco*-heptopyranoside 7-phosphate (**14**) ------------------------ S10
Procedure for direct phosphorylation and deprotection of 1-*O*-methyl 2,3,4-tri-*O*-benzyl- D/L-*glycero*-α-D-*gluco*-heptopyranoside (**9**) into 1-*O*-methyl D-*glycero*-α-D-*gluco*-heptopyranoside 7-phosphate (**14**) ------------------------- S11

NMR spectral data for compounds

^{1}H NMR spectrum of **6** -- S12
^{13}C NMR spectrum of **6** --- S13
DEPT NMR spectrum of **6** --- S14
COSY NMR spectrum of **6** --- S15
NOESY NMR spectrum of **6** -- S16
HSQC NMR spectrum of **6** --- S17

^{1}H NMR spectrum of **8** -- S18
^{13}C NMR spectrum of **8** --- S19

DEPT NMR spectrum of **8** ---------- S20
COSY NMR spectrum of **8** ---------- S21
NOESY NMR spectrum of **8** ---------- S22
HSQC NMR spectrum of **8** ---------- S23

^{1}H NMR spectrum of **9** ---------- S24
^{13}C NMR spectrum of **9** ---------- S25
DEPT NMR spectrum of **9** ---------- S26
COSY NMR spectrum of **9** ---------- S27
NOESY NMR spectrum of **9** ---------- S28
HSQC NMR spectrum of **9** ---------- S29

^{1}H NMR spectrum of **12** ---------- S30
^{13}C NMR spectrum of **12** ---------- S31
DEPT NMR spectrum of **12** ---------- S32
COSY NMR spectrum of **12** ---------- S33
HSQC NMR spectrum of **12** ---------- S34

^{1}H NMR spectrum of **13** ---------- S35
^{13}C NMR spectrum of **13** ---------- S36
DEPT NMR spectrum of **13** ---------- S37
^{31}P NMR spectrum of **13** ---------- S38
COSY NMR spectrum of **13** ---------- S39
NOESY NMR spectrum of **13** ---------- S40
HSQC NMR spectrum of **13** ---------- S41
^{31}P-HMBC NMR spectrum of **13** ---------- S42

^{1}H NMR spectrum of **14** ---------- S43
1H DOSY-filtered NMR spectrum of **14** ---------- S44
^{13}C NMR spectrum of **14** ---------- S45
DEPT NMR spectrum of **14** ---------- S46
^{31}P NMR spectrum of **14** ---------- S47
COSY NMR spectrum of **14** ---------- S48
NOESY NMR spectrum of **14** ---------- S49
HSQC NMR spectrum of **14** ---------- S50
HSQC-TOCSY NMR spectrum of **14** ---------- S51
^{1}H,^{31}P-HMBC NMR spectrum of **14** ---------- S52

Reagents and equipment

All reactions were carried out under a nitrogen atmosphere using oven-dried glassware and using standard Schlenk techniques unless otherwise mentioned. Reaction temperature refers to the temperature of the oil bath. All reagents and catalysts were purchased from Sigma-Aldrich, Acros, J&K Scientific and TCI Europe and used without further purification unless otherwise mentioned. Column chromatography was performed on silica gel 60, 0.040–0.063 mm (Merck, Germany), TLC analysis was performed on Merck silica gel 60/Kieselguhr F254, 0.25 mm. ^{1}H and ^{13}C NMR spectra were recorded on a 300 MHz (300.1, 75.5 and 30.4 MHz, respectively) and 400 MHz (400.1, 100.6 and 40.6 MHz, respectively) spectrometers (Bruker BioSpin GmbH, Germany) in $CDCl_3$, DMSO-d_6, and methanol-d_4 solutions using 0.05% Me_4Si as the external or internal standard. Determinations of structures and stereochemistry of obtained compounds and assignments of ^{1}H and ^{13}C signals were made with the aid of 2D COSY, TOCSY, NOESY, edited-HSQC, HSQC-TOCSY, and HMBC spectra where necessary. High-resolution mass spectra were recorded on a Bruker Daltonics micrOTOF-Q II device by electrospray ionization mass spectrometry (ESI-MS). Measurements were carried out in positive ion mode via syringe injection from in acetonitrile-water solutions.

Procedures for synthesis and compounds characterization

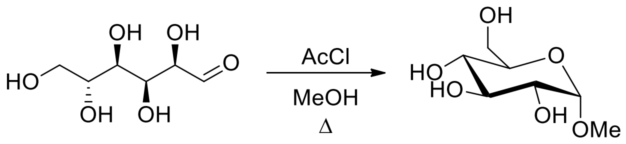

1-*O*-Methyl α-D-*gluco*-pyranoside (4)

A mixture formed by mixing acetyl chloride (3.27 g, 41.6 mmol) and 18 mL of methanol was added dropwise to a suspension of D-glucose (10.00 g, 55.5 mmol) in 75 mL of methanol at 0°C. The reaction mixture was refluxed for 50 h and cooled to -20°C. The formed precipitate was filtered off and washed with cold methanol. The product was further purified by recrystallization from ethanol. Yield: 5.20 g, 48%, single α-isomer.

^{1}H NMR (300 MHz, DMSO-d6) δ 4.83 (d, *J* = 5.6 Hz, 1H), 4.72 (d, *J* = 4.8 Hz, 1H), 4.67 (d, *J* = 6.4 Hz, 1H), 4.52 (d, *J* = 3.6 Hz, 1H), 4.44 (t, *J* = 5.9 Hz, 1H), 3.63 (ddd, *J* = 11.6, 5.7, 2.1 Hz, 1H), 3.52 – 3.28 (m, 3H), 3.26 (s, 3H), 3.18 (ddd, *J* = 9.8, 6.4, 3.7 Hz, 1H), 3.04 (ddd, *J* = 9.8, 8.7, 5.6 Hz, 1H).

HRMS (ESI) of $C_7H_{14}O_6$, *m/z*: calcd $[M+Na]^+$ 217.0683, found 217.0686; calcd $[M+H]^+$ 195.0863, found 195.0867.

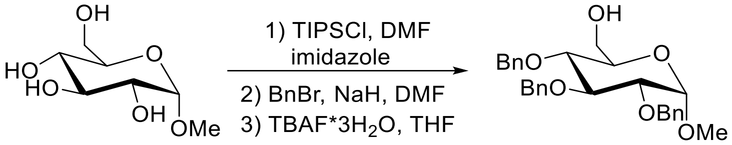

1-*O*-Methyl 2,3,4-tri-*O*-benzyl-α-D-*gluco*-pyranoside (6)

A solution of methyl α-D-glucopyranoside (1.00 g, 5.15 mmol) in 4 ml of absolute DMF at 0°C was treated with triisopropylsilyl chloride dropwise under argon. The mixture was heated to room temperature and kept stirring for 12 h. Water (20 ml) was added to the reaction mixture, the mixture was partitioned between water and CH_2Cl_2 (3×20 ml), organic layer was collected, dried over Na_2SO_4, and solvent evaporated on a rotary evaporator. The intermediate product was dried in high vacuum, dissolved in 5 ml of absolute DMF and added dropwise to a suspension of NaH (60% in oil, 1.03 g, 25.75 mmol, pre-washed with petroleum ether) in 20 ml of absolute DMF and benzyl bromide (4.4 g, 25.75 mmol) at 0°C. The mixture was kept under vigorous stirring for 12 h at room temperature. Water (50 ml) was added to the reaction mixture, and the resulting mixture was extracted with Et_2O (3×50 ml). The combined organic layers were washed with water and saturated NaCl solution, dried over Na_2SO_4, and solvents were evaporated. The residue was dissolved in THF (5 ml) and tetrabutylammonium fluoride trihydrate (3.25 g, 10.30 mmol) was added in portions. The mixture was stirred for 12 h, treated with 20 ml of a saturated solution of NH_4Cl, and partitioned between water/Et_2O (3 organic washes × 20 ml). The combined organic layers were dried over Na_2SO_4 and solvents were evaporated. The product was purified by column chromatography on silica gel using petroleum ether/ethyl acetate (4:3) eluting system. Yield: 1.9 g, 79% in 3 steps.

^{1}H NMR (400 MHz, $CDCl_3$) δ 7.40 – 7.23 (m, 15H, 3×Ph), 4.98 (d, J = 10.9 Hz, 1H, H_a from CH_2), 4.88 (d, J = 11.1 Hz, 1H, H_a from CH_2), 4.83 (d, J = 10.9 Hz, 1H, H_b from CH_2), 4.79 (d, J = 12.2 Hz, 1H, H_a from CH_2), 4.65 (d, J = 12.0 Hz, 1H, H_b from CH_2), 4.64 (d, J = 11.1 Hz, 1H, H_b from CH_2), 4.57 (d, J = 3.6 Hz, 1H, CH(1)), 4.00 (t, J = 9.2 Hz, 1H, CH(3)), 3.83 – 3.65 (m, 2H, $CH_{2,}$ H_b from CH_2), 3.68 – 3.61 (m, 1H, CH(5)), 3.56 – 3.47 (m, 2H, CH(2) and CH(4)), 3.35 (s, 3H, CH_3), 1.77 (br.s, 1H, OH).

^{13}C NMR (101 MHz, $CDCl_3$) δ 138.80, 138.21, 138.17, 128.51, 128.44, 128.15, 128.06, 128.00, 127.97, 127.87, 127.65 (Ph), 98.22 (C(1)), 82.00 (C(3)), 80.04 (C(4)), 77.47 (C(2)), 75.77 (C(Bn)), 75.06 (C(Bn)), 73.45 (C(Bn)), 70.75 (C(5)), 61.87 (C(Bn)), 55.22 (C(Me)).

HRMS (ESI) of $C_{28}H_{32}O_6$, *m*/*z*: calcd $[M+Na]^+$ 487.2091, found 487.2092; calcd $[M+H]^+$ 465.2272, found 465.2275.

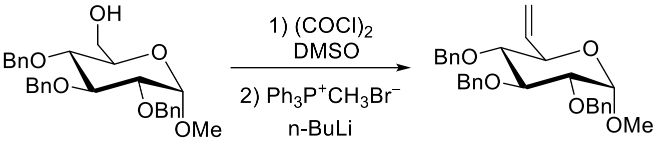

1-*O*-Methyl 2,3,4-tri-*O*-benzyl-6-deoxy-α-D-*gluco*-hept-6-enopyranoside (8)

Dimethylsulfoxide (0.51 g, 6.54 mmol) was added dropwise to a solution of oxalyl chloride (0.73 g, 4.09 mmol) in absolute CH_2Cl_2 (32 ml) at –78°C. After 1 h, a solution of methyl 2,3,4-tri-*O*-benzyl-α-glucopyranoside (1.90 g, 4.09 mmol) in CH_2Cl_2 (13 ml) was added dropwise at –78°C to the reaction mixture, and the solution was stirred for another 1 h. Then triethylamine (1.24 g, 12.27 mmol) was added, the reaction mixture was left to warm up to room temperature and kept for 1 h. Saturated $NaHCO_3$ aqueous solution (40 ml) was added, the organic layer was separated, the aqueous layer was extracted with CH_2Cl_2 (3 × 40 ml). The combined organic layers were washed with saturated NaCl solution, dried over Na_2SO_4 and the solvents were evaporated *in vacuo*. The aldehyde product was used in the further Horner–Wadsworth–Emmons reaction without purification.

n-Butyllithium (2.5 M in hexane, 2.5 ml, 6.13 mmol) was added dropwise to a suspension of methyltriphenylphosphonium bromide (2.20 g, 6.13 mmol) in absolute THF (25 ml) at 0°C and the reaction mixture was kept under vigorous stirring at 0°C for 10 min. Then the mixture was cooled to –78°C and a solution of the aldehyde product in 10 ml of absolute THF was added dropwise. The mixture was left to warm up to room temperature and kept for 12 hours. The reaction mixture was quenched with 60 ml of a saturated NH_4Cl aqueous solution. The product was partitioned between water and Et_2O (3×60 ml). The combined organic layers were dried over Na_2SO_4, then 2/3 volume of Et_2O was evaporated *in vacuo*, the residue was re-solubilized in petroleum ether, and the mixture was cooled to -20°C. The precipitate of triphenylphosphine oxide was filtered off, washed with petroleum ether, and the solvent from the filtrate was evaporated. The product was purified on a silica gel column using petroleum ether/ethyl acetate (10:1) eluting system. Yield: 0.85 g, 45% in 2 steps.

^{1}H NMR (400 MHz, $CDCl_3$) δ 7.46 – 7.23 (m, 15H, 3×Ph), 5.90 (ddd, J = 17.1, 10.4, 6.5 Hz, 1H, CH=), 5.41 (dt, J = 17.2, 1.5 Hz, 1H, =CH_2), 5.25 (ddd, J = 10.5, 1.8, 1.0 Hz, 1H, =CH_2), 4.96 (d, J = 10.9 Hz, 1H, H_a from CH_2), 4.83 (d, J = 10.9 Hz, 1H, H_b from CH_2), 4.82 – 4.74 (m, 1H, H_a from CH_2), 4.78 (d, J = 10.8 Hz, 1H, H_a from CH_2), 4.67 (d, J = 12.1 Hz, 1H, H_b from CH_2), 4.64 – 4.57 (m, 1H, CH(1)), 4.71 – 4.61 (m, 1H, H_b from CH_2), 4.14 – 4.04 (m, 1H, CH(5)), 3.99 (t, J = 9.3 Hz, 1H, CH(3)), 3.53 (dd, J = 9.7, 3.6 Hz, 1H, CH(2)). 3.37 (s, 3H, Me), 3.25 (dd, J = 9.8, 8.9 Hz, 1H, CH(4)).

^{13}C NMR (101 MHz, $CDCl_3$) δ 138.87, 138.25, 135.32, 128.52, 128.45, 128.41, 128.16, 128.06, 128.02, 127.96, 127.78, 127.72, 127.66 (Ph), 118.16

(=CH_2), 98.14 (C(1)), 82.33 (C(4)), 81.76 (C(3)), 79.94 (C(2)), 75.91 (CH_2), 75.18 (CH_2), 73.45 (CH_2), 71.45 (C(5)), 55.24 (CH_3).

HRMS (ESI) of $C_{28}H_{32}O_6$, *m*/*z*: calcd $[M+H]^+$ 461.2323, found 461.2325.

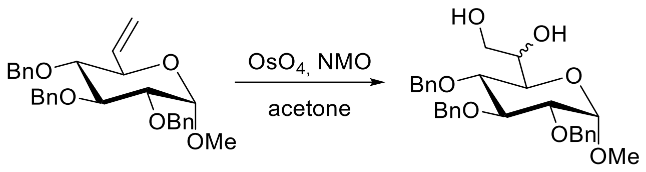

1-*O*-Methyl 2,3,4-tri-*O*-benzyl-D/L-*glycero*-α-D-*gluco*-heptopyranoside (9)

A solution of OsO_4 (49.7 mg, 0.19 mmol) in 2.5 ml of *tert*-butanol, NMO (50% aqueous solution, 1.65 ml, 7.82 mmol) and 0.9 ml of H_2O were added to a solution of 1-*O*-methyl 2,3,4-tri-*O*-benzyl-6-deoxy-α-D-*gluco*-hept-6-enopyranoside (1.80 g, 3.91 mmol) in 22 ml of acetone and the reaction mixture was kept stirring for 12 hours at room temperature. Then 30 ml of 5% $Na_2S_2O_3$ aqueous solution were added to the reaction mixture and left for 30 min. The resulting mixture was partitioned between water and CH_2Cl_2 (3×30 ml). The combined organic layers were washed with saturated $NaHCO_3$ and NaCl aqueous solutions, dried over Na_2SO_4 and solvents were evaporated *in vacuo*. The product was purified on a silica gel column using petroleum ether/ethyl acetate (1:2) eluting system. Yield: 1.8 g, 93%, a mixture of isomers with *dr* = 5:1 (D-*glycero* : L-*glycero*).

Major D-glycero isomer:

^1H NMR (400 MHz, $CDCl_3$) δ 7.41 – 7.24 (m, 15H, Ph), 5.03 (d, *J* = 10.9 Hz, 1H, H_a from CH_2), 4.81 – 4.77 (m, 1H, H_b from CH_2), 4.79 (d, *J* = 11.0 Hz, 1H, H_a from CH_2), 4.77 – 4.74 (m, 1H, H_a from CH_2), 4.65 (d, *J* = 11.0 Hz, 1H, H_b from CH_2), 4.64 (d, *J* = 12.0 Hz, 1H, H_b from CH_2), 4.54 (d, *J* = 3.6 Hz, 1H, CH(1)), 4.05 – 3.98 (m, 1H, CH(3)), 3.85 – 3.77 (m, 1H, CH(5)), 3.80 – 3.71 (m, 1H, CH-OH), 3.66 – 3.55 (m, 2H, CH_2), 3.64 – 3.59 (m, 1H, CH(4)), 3.56 – 3.45 (m, 3H, CH(2)), 3.37 (s, 3H, OCH_3).

^{13}C NMR (101 MHz, $CDCl_3$) δ 138.51, 137.99, 137.44, 128.65, 128.55, 128.53, 128.50, 128.44, 128.17, 128.14, 128.07, 127.95, 127.87, 127.75, 127.65 (Ph), 97.79 (C(1)), 82.24 (C(3)), 80.15 (C(4)), 79.75 (C(2)), 75.70 (CH_2), 74.91 (CH_2), 73.37 (CH_2), 72.68 (C(5)), 69.84 (CH-OH), 62.91 (CH_2-OH), 55.37 (CH_3).

HRMS (ESI) of $C_{29}H_{34}O_7$, *m*/*z*: calcd $[M+Na]^+$ 517.2196, found 517.2198; calcd $[M+H]^+$ 495.5772, found 495.5772.

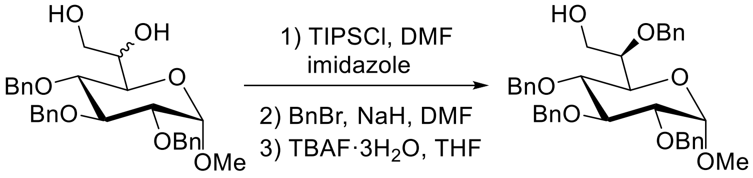

1-*O*-Methyl 2,3,4,6-tetra-*O*-benzyl-D-*glycero*-α-D-*gluco*-heptopyranoside (12)

A mixture of isomers of 1-methyl 2,3,4-tri-O-benzyl-D/L-*glycero*-α-D-*gluco*-heptopyranoside (1.0 g, 5.15 mmol) was dissolved in absolute DMF (4 ml) at 0°C and triisopropylsilyl chloride (1.04 g, 5.40 mmol) was added dropwise under argon atmosphere. The mixture was left to warm up to room temperature and kept stirring for 12 h. Then the solvent was partially evaporated, 20 ml of H_2O were added to the reaction mixture and it was partitioned between water/CH_2Cl_2 (3×20 ml of organic wash). The combined organic layers were dried over Na_2SO_4, and the solvents were evaporated *in vacuo*. The resulting intermediate product was additionally dried in high vacuum, dissolved in absolute DMF (5 ml), and added dropwise at 0°C to a suspension of NaH (60% in oil, 1.03 g, 25.75 mmol, pre-washed with petroleum ether) in absolute DMF (20 ml), and then, after 5 min. the mixture was treated with benzyl bromide (4.4 g, 25.75 mmol). The mixture was left to warm up to room temperature and was kept vigorously stirring for 12 hours. Then H_2O (50 ml) was added, and the mixture was partitioned between water/Et_2O (3×50 ml of organic wash). The combined organic layers were washed with water and saturated NaCl solution, dried over Na_2SO_4 and solvents were evaporated *in vacuo*. The residue was dissolved in THF (11 ml) and TBAF·3H_2O (1.22 g, 3.86 mmol) was added in portions. The reaction mixture was stirred for 12 h, and 20 ml of a saturated aqueous NH_4Cl solution was added. The mixture was extracted between water/Et_2O (3×20 ml of organic wash), dried over Na_2SO_4 and solvents were evaporated *in vacuo*. The product was purified on a silica gel column using petroleum ether/ethyl acetate (2:1) eluting system. Yield: 0.88 g, 37% in 3 steps, dr = 10:1 (D-*glycero* : L-*glycero*).

^{1}H NMR (400 MHz, $CDCl_3$) δ 7.50 – 7.11 (m, 20H, Ph), 5.00 (d, *J* = 10.8 Hz, 1H, H_a from CH_2), 4.90 (d, *J* = 10.9 Hz, 1H, H_b from CH_2), 4.80 (d, *J* = 10.9 Hz, 1H, H_b from CH_2), 4.77 (d, *J* = 10.1 Hz, 1H, H_a from CH_2), 4.70 (d, *J* = 11.8 Hz, 1H, H_a from CH_2), 4.61 (d, *J* = 10.9 Hz, 1H, H_a from CH_2), 4.61 – 4.52 (m, 1H, CH(1)). 4.56 (d, *J* = 11.8 Hz, 1H, H_b from CH_2), 4.69 – 4.61 (m, 1H, H_b from CH_2), 4.01 (t, *J* = 9.2 Hz, 1H, CH(3)), 3.78 – 3.63 (m, 1H, H_a from CH_2), 3.63 – 3.49 (m, 1H, H_b from CH_2), 3.55 – 3.45 (m, 1H, CH(2)), 3.52 – 3.48 (m, 2H, CH(4,5)), 3.38 (s, 3H, OCH_3).

^{13}C NMR (101 MHz, $CDCl_3$) δ 138.65, 138.27, 138.15, 137.93, 128.55, 128.50, 128.09, 128.05, 128.02, 128.00, 127.98, 127.88, 127.86, 127.84, 127.80, 127.72 (Ph), 97.82 (C(1)), 82.57 (C(3)), 80.16 (C(2)), 78.68 (C(6)), 77.69 (C(4)),

75.88 (CH_2), 74.82 (CH_2), 73.38 (CH_2), 72.23 (CH_2), 70.68 (C(5)), 61.68 (C(7)), 55.26 (CH_3).

HRMS (ESI) of $C_{36}H_{40}O_7$, *m/z*: calcd $[M+Na]^+$ 607.2666, found 607.2671; calcd $[M+H]^+$ 585.2847, found 585.2850.

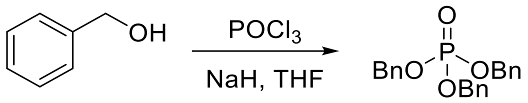

Tribenzyl phosphate (16)

Benzyl alcohol (3.48 g, 32.19 mmol) was added dropwise to a suspension of NaH (60% in oil, 1.37 g, 34.33 mmol, pre-washed with petroleum ether) in THF (150 mL) at 0°C. The mixture was left to warm up to room temperature and kept stirring for 2 h. Then $POCl_3$ (3.65 g, 10.73 mmol) was added dropwise and the reaction mixture was kept stirring for 24 h. The reaction was quenched with a saturated aqueous NaCl solution (100 ml) and partitioned between water/EtOAc (3×100 ml of organic wash). The combined organic layers were dried over Na_2SO_4 and solvents were evaporated *in vacuo*. The product was purified on a silica gel column using petroleum ether/ethyl acetate (2:1) eluting system. Yield: 1.94 g, 49%.

^{1}H NMR (300 MHz, $CDCl_3$) δ 7.53 – 7.19 (m, 15H, Ph), 5.00 (d, *J* = 8.1 Hz, 6H, CH_2).

^{31}P NMR (122 MHz, $CDCl_3$) δ -0.91 (hept, *J* = 8.1 Hz).

HRMS (ESI) of $C_{21}H_{21}O_4P$, *m/z*: calcd $[M+Na]^+$ 391.1070, found 391.1073; calcd $[M+H]^+$ 369.1250, found 369.1251.

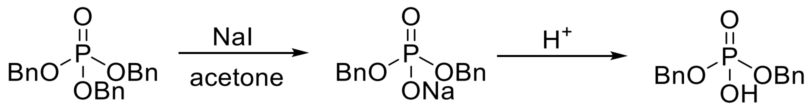

Dibenzyl phosphate, sodium salt (17)

A 1.1 equiv. excess of NaI (215 mg, 1.43 mmol) was added to a solution of tribenzyl phosphate (480 mg, 1.30 mmol) in absolute acetone. The mixture was heated to boiling and kept for 30 min, and then cooled down to room temperature. Acetone was evaporated under reduced pressure, the dry residue was dissolved in water (5 ml) and washed with Et_2O. The aqueous layer was collected.

Dibenzyl phosphate (18)

Acidification of **17** was further performed by either of the procedures:

1) The aqueous layer was acidified with 10% HCl and partitioned between water/Et_2O (3×5 ml of organic wash). The combined organic layers were dried over Na_2SO_4 and and solvents were evaporated *in vacuo*. The product was purified by recrystallization from petroleum ether/CH_2Cl_2 (1:1). Yield: 160 mg, 44%.
2) New commercially available cation-exchange Dowex resin was activated and pre-washed subsequently: with 100 ml of 1M NaOH solution, with distilled water till neutral pH was reached, with 100 ml of 1M HCl solution + 20% isopropanol additive to wash the degraded monomeric residues, with distilled water till neutral pH was reached. The aqueous layer with **17** was passed through a column with the Dowex resin and eluted with 10% EtOH in water, the target fraction was monitored by the pH change of the eluate. Solvents were evaporated *in vacuo* to give pure **18**. Yield: 316 mg, 87%.

^{1}H NMR (300 MHz, $CDCl_3$) δ 9.63 (br.s, 1H, OH), 7.39 – 7.31 (m, 15H, Ph), 5.04 (d, *J* = 7.6 Hz, 1H, CH_2).

HRMS (ESI) of $C_{14}H_{15}O_4P$, *m*/*z*: calcd $[M+Na]^+$ 301.0600, found 301.0602; calcd $[M+H]^+$ 279.0781, found 279.0783.

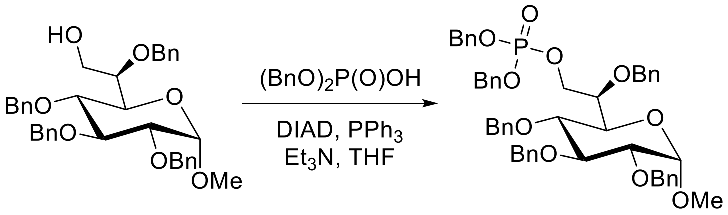

1-*O*-Methyl 2,3,4,6-tetra-*O*-benzyl-D-*glycero*-α-D-*gluco*-heptopyranoside 7-phosphate (13)

Diethyl azodicarboxylate (105 mg, 0.52 mmol) was added dropwise to a pre-cooled solution of 1-*O*-methyl 2,3,4,6-tetra-*O*-benzyl-D-*glycero*-α-D-*gluco*-heptopyranoside (104 mg, 0.18 mmol), dibenzyl phosphate (149 mg, 0.54 mmol), triphenylphosphine (140 mg, 0.54 mmol) and triethylamine (108 mg, 1.07 mmol) in absolute THF (2 ml). The reaction was kept for 12 h and evaporated to dryness. The product was purified on a silica gel using petroleum ether/ethyl acetate (5:3) eluting system. Yield: 88 mg, 59%.

^{1}H NMR (400 MHz, $CDCl_3$) δ 7.50 – 7.19 (m, 30H, Ph), 5.16-4.96 (m, 2H, 2×H_b from CH_2 of $(BnO)_2P$), 5.05 (d, *J* = 10.6 Hz, 1H, H_b from CH_2), 5.02 (d, *J* = 8.0 Hz, 1H, H_a from CH_2 of $(BnO)_2P$), 5.01 (d, *J* = 7.9 Hz, 1H, H_a from CH_2 of $(BnO)_2P$), 4.93 (d, *J* = 10.9 Hz, 1H, H_b from CH_2), 4.85 (d, *J* = 10.8 Hz, 1H, H_b from CH_2), 4.87 (d, *J* = 12.1 Hz, 1H, H_a from CH_2), 4.72 (d, *J* = 11.8 Hz, 1H, H_a from CH_2), 4.71 (d, *J* = 12.1 Hz, 1H, H_b from CH_2), 4.66 (d, *J* = 10.8 Hz, 1H, H_a

from CH_2), 4.66 (d, J = 10.8 Hz, 1H, H_b from CH_2), 4.65 (d, J = 11.9 Hz, 1H, CH(1)), 4.30 – 4.15 (m, 2H, CH_2(7)), 4.10 – 3.90 (m, 3H, CH(6) + CH(3)+ CH(5)), 3.60 (dd, 1H, J = 10.3 Hz, J = 10.3 Hz, CH(4)), 3.52 (dd, 1H, J = 9.6 Hz, J = 9.7 Hz CH(2)), 3.42 (s, 3H, OCH_3).

^{13}C NMR (101 MHz, $CDCl_3$) δ 138.71, 138.24, 138.15, 138.12, 137.90, 128.61, 128.58, 128.53, 128.50, 128.44, 128.42, 128.33, 128.09, 127.99, 127.95, 127.90, 127.77, 127.70, 127.66, 127.60 (Ph), 97.86 (C(1)), 82.43 (C(3)), 80.04 (C(2)), 78.10 (d, $J_{C, P}$ = 7.9 Hz, C(6)), 77.88 (C(4), 75.79 (CH_2), 74.80 (CH_2), 73.37 (CH_2), 72.86 (CH_2), 70.52 (C(5)), 69.27 (d, $J_{C, P}$ = 3.7 Hz, CH_2 of $(BnO)_2P$)), 69.20 (d, $J_{C, P}$ = 3.7 Hz, CH_2 of $(BnO)_2P$)), 67.52 (d, $J_{C, P}$ = 5.8 Hz, C(7)), 55.21 (CH_3).

^{31}P NMR (162 MHz, D_2O) δ 0.85.

HRMS (ESI) of $C_{50}H_{53}O_{10}P$, *m/z*: calcd $[M+H]^+$ 845.3449, found 845.3455.

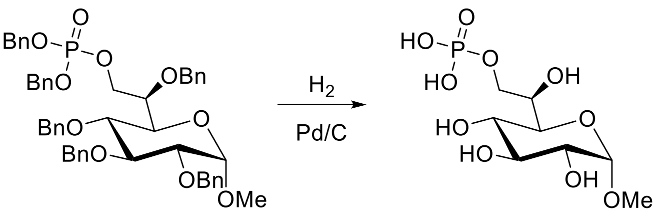

1-*O*-Methyl D-*glycero*-α-D-*gluco*-heptopyranoside 7-phosphate (14)

Palladium on carbon (10%, 283 mg, 0.27 mmol) powder was added to a solution of heptose phosphate (300 mg, 0.35 mmol) in a mixture of ethyl acetate (4 ml), ethanol (7 ml) and water (3 ml). The reaction mixture was stirred under a hydrogen atmosphere for 3 hours. The reaction mixture was filtered through a zeolite filter cake and the solvent was evaporated in vacuo and additionally freeze dryed to obtaine white amorophous poweder. Yield: 101 mg, 99%.

^{1}H NMR (400 MHz, D_2O) δ 4.67 (d, J = 3.1 Hz, 1H, CH(1)), 4.09-4.02 (m, 1H, CH(6)), 4.06-3.97 (m, 1H, H_a from CH_2(7)), 3.95-3.88 (m, 1H, H_b from CH_2(7)), 3.64-3.57 (dd, J = 9.9 Hz, J = 2.9 Hz, 1H, CH(4)), 3.55-3.47 (dd, J = 9.5 Hz, J = 9.7 Hz, 1H, CH(3)), 3.46-3.36 (m, 2H, CH(2) and CH(5)) 3.29 (s, 3H, CH_3).

^{13}C NMR (101 MHz, D_2O) δ 99.20 (C(1)), 73.25 (C(4)), 71.26 (C(3)), 70.96 (C(2)), 70.42 (d, J = 7.9 Hz, C(6)), 70.23 (C(5)), 66.30 (d, J = 4.8 Hz, C(7)), 55.08 (CH_3).

^{31}P NMR (162 MHz, D_2O) δ 0.43.

HRMS (ESI) of $C_8H_{17}O_{10}P$, *m/z*: calcd $[M+Na]^+$ 327.0452, found 327.0450; calcd $[M+H]^+$ 305.0632, found 305.0628.

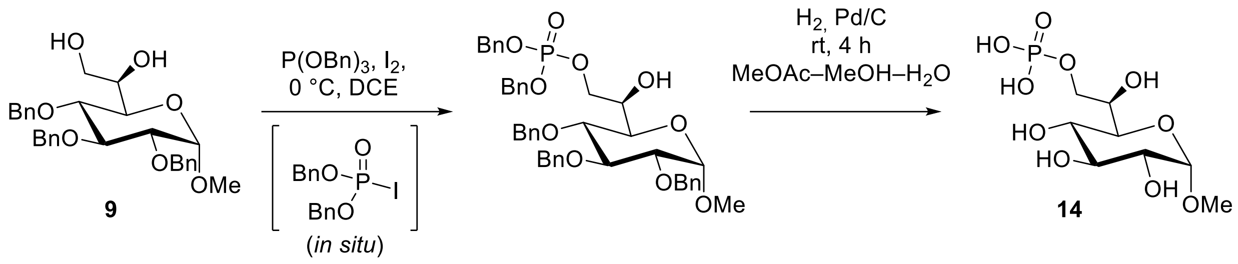

Procedure for direct phosphorylation and deprotection of 1-*O*-methyl 2,3,4-tri-*O*-benzyl-D/L-*glycero*-α-D-*gluco*-heptopyranoside (9) into 1-*O*-methyl D-*glycero*-α-D-*gluco*-heptopyranoside 7-phosphate (14)

A solution of crystalline sublimated iodine (275 mg, 1.09 mmol) in dry 1,2-dichloroethane (DCE) (10 mL) was added to a pre-cooled (0°C) solution of purified $P(OBn)_3$ (412 mg, 1.12 mmol) in dry DCE (20 mL) under an argon atmosphere. The reaction mixture was stirred 15 min at 0°C, and then 10 min at 20°C. Then the solution was added dropwise under an argon atmosphere at 20°C to a solution of diol **9** (540 mg, 1.09 mmol) in anhydrous DCE (15 mL) and pyridine (95 mL, 1.18 mmol). After stirring for 45 min at 20°C, the pyridinium salts were filtered off and the organic mixture was washed with water (2×25 mL) and dilute sodium bicarbonate solution (2×25 mL), and then dried using anhydrous $MgSO_4$ and filtered. The solvent was evaporated *in vacuo*. The intermediate product – 1-*O*-methyl 2,3,4-tri-*O*-benzyl-D-*glycero*-α-D-*gluco*-heptopyranoside 7-(dibenzyl)-phosphate – was purified by flash chromatography on a silica gel column in petroleum ether/ethyl acetate (5:3) eluting system to obtain a crude product, which was immediately used in the next synthetic step. The solvent was evaporated *in vacuo*, and the obtained crude benzylated heptose phosphate was dissolved in a mixture of methyl acetate (7.5 ml), methanol (12.5 ml) and water (5.5 ml), and then palladium on carbon powder (10%, 495 mg, 0.47 mmol) was added. The reaction mixture was stirred under hydrogen atmosphere for 4 hours. The reaction mixture was filtered through a zeolite filter cake and the solvent was evaporated *in vacuo* and additionally freeze dried to obtain pure desired product **14** in the form of white amorphous powder (spectral data identical to the above described). Yield: 175 mg, 53% (on 2 steps).

^{1}H NMR spectrum of 1-*O*-methyl 2,3,4-tri-O-benzyl-α-D-*gluco*-pyranoside (**6**)

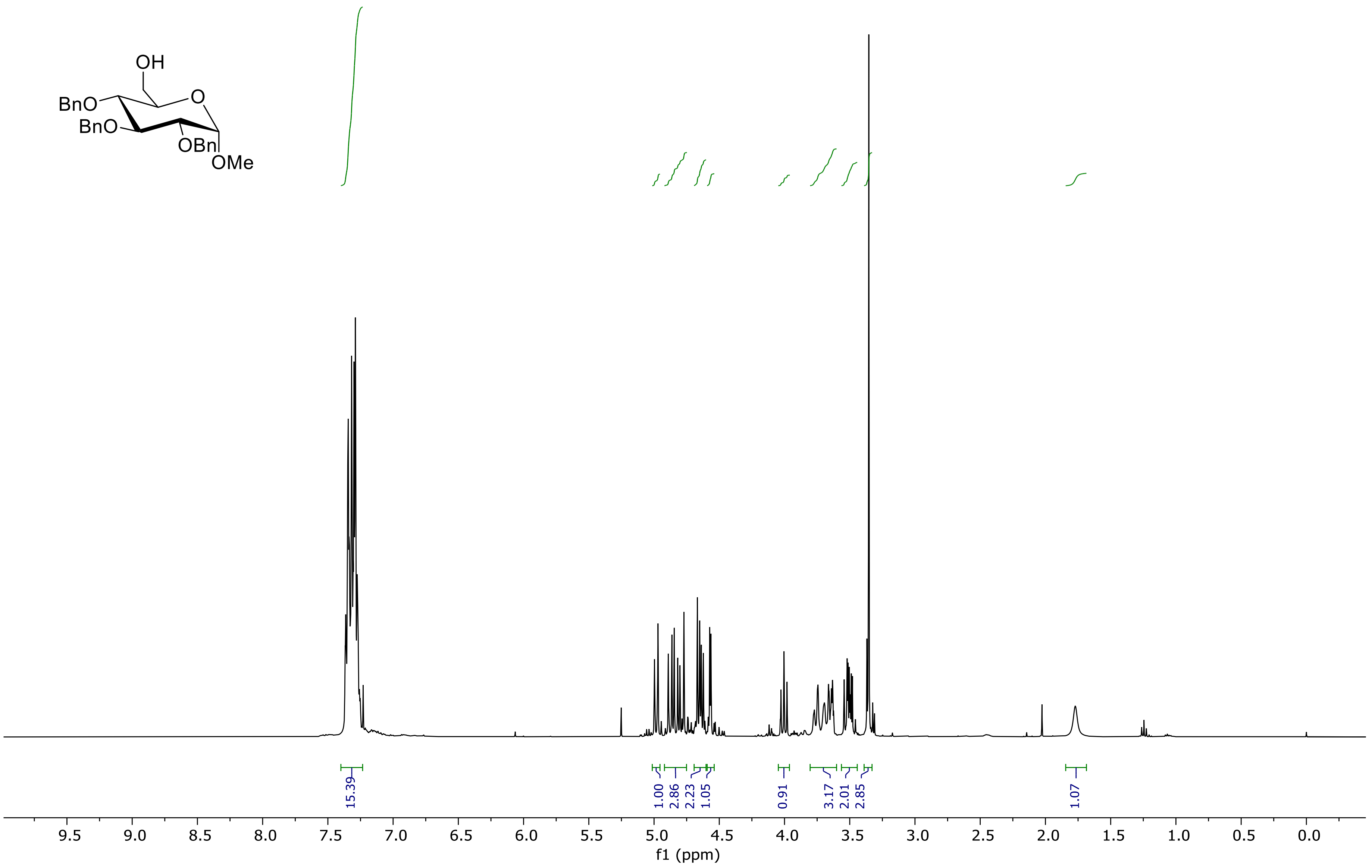

^{13}C NMR spectrum of 1-*O*-methyl 2,3,4-tri-O-benzyl-α-D-*gluco*-pyranoside (**6**)

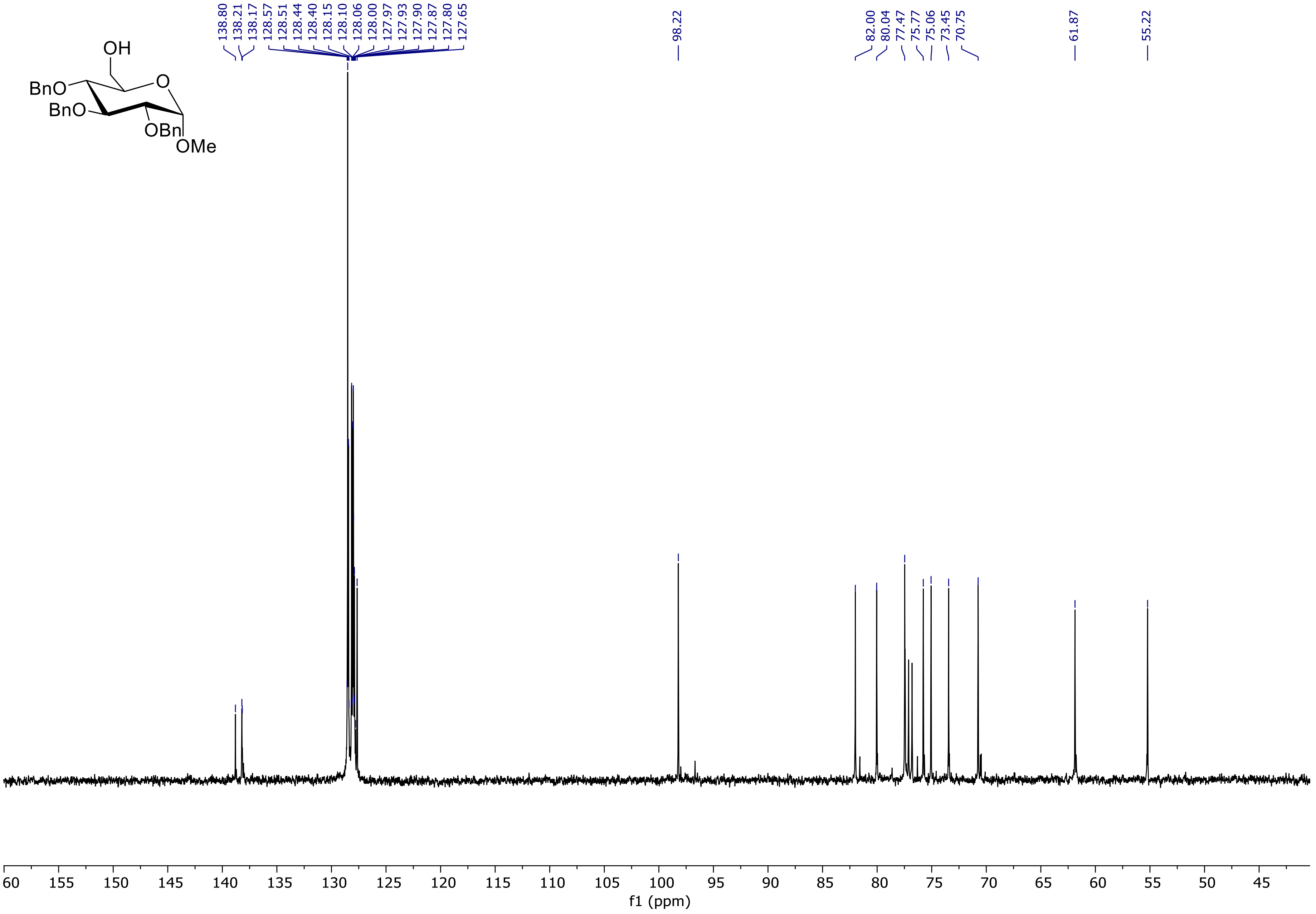

DEPT ^{13}C NMR spectrum of 1-*O*-methyl 2,3,4-tri-O-benzyl-α-D-*gluco*-pyranoside (**6**)

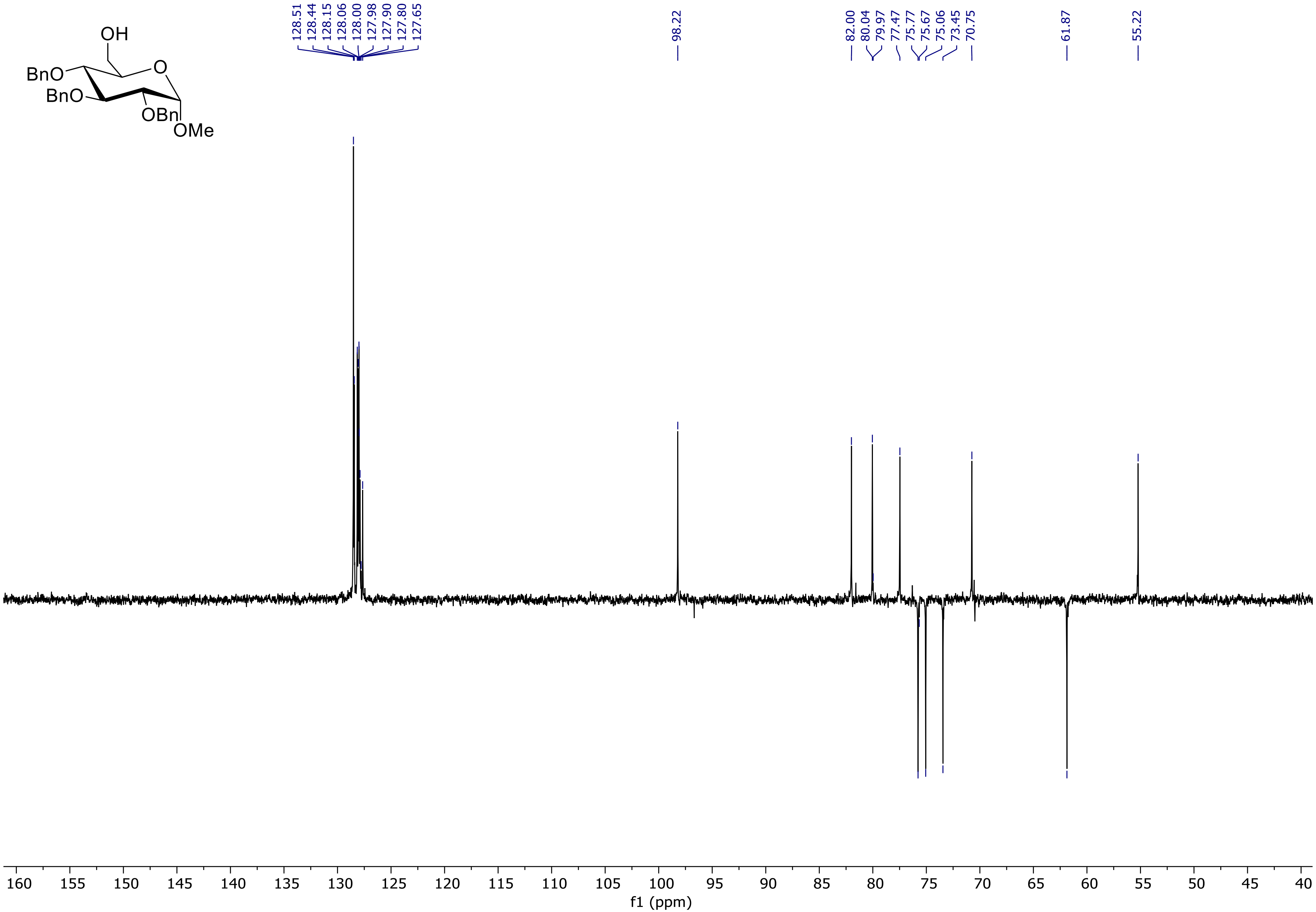

COSY NMR spectrum of 1-*O*-methyl 2,3,4-tri-O-benzyl-α-D-*gluco*-pyranoside (**6**)

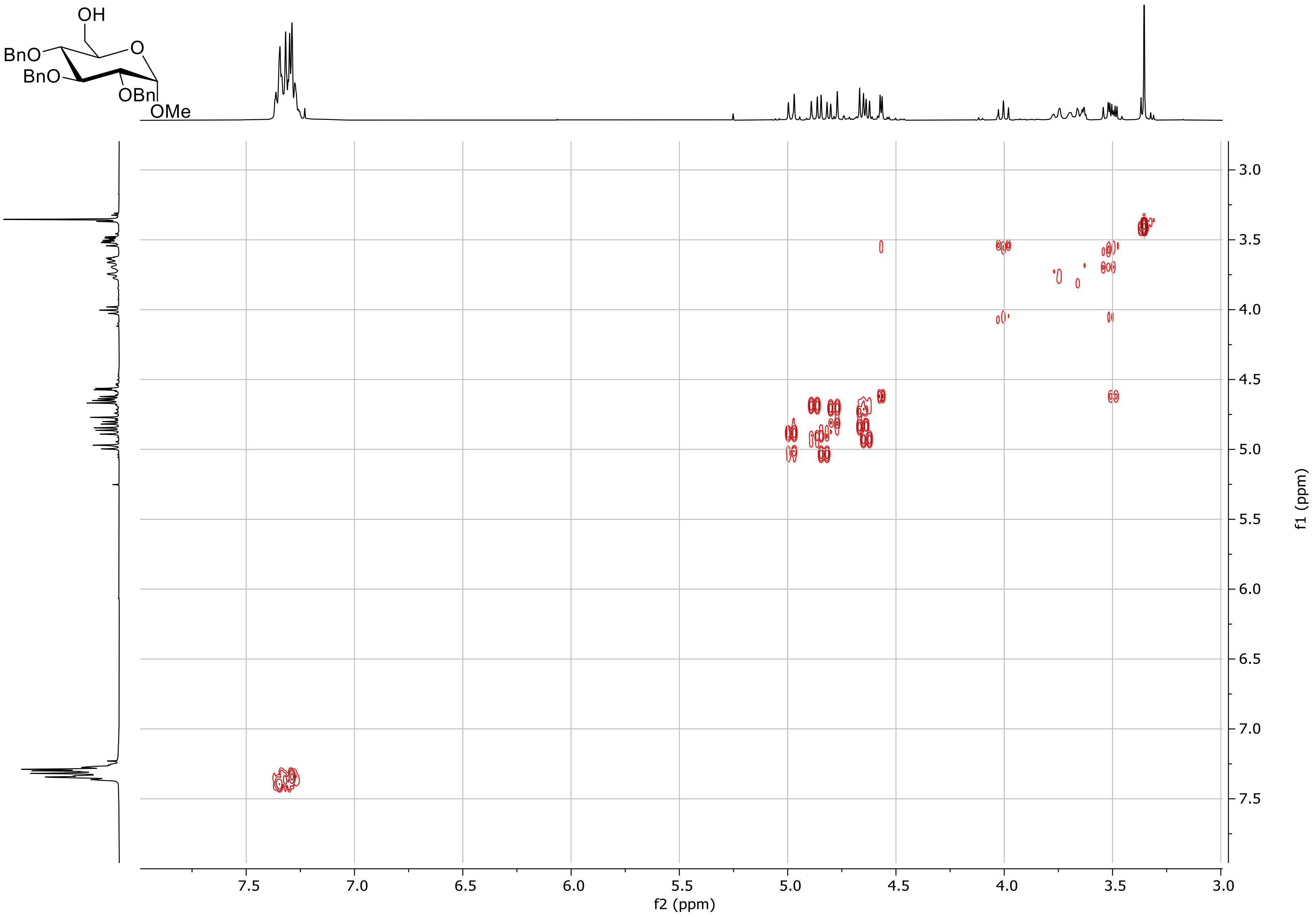

NOESY NMR spectrum of 1-*O*-methyl 2,3,4-tri-O-benzyl-α-D-*gluco*-pyranoside (**6**)

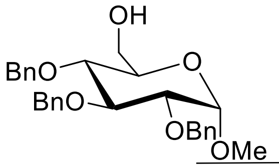

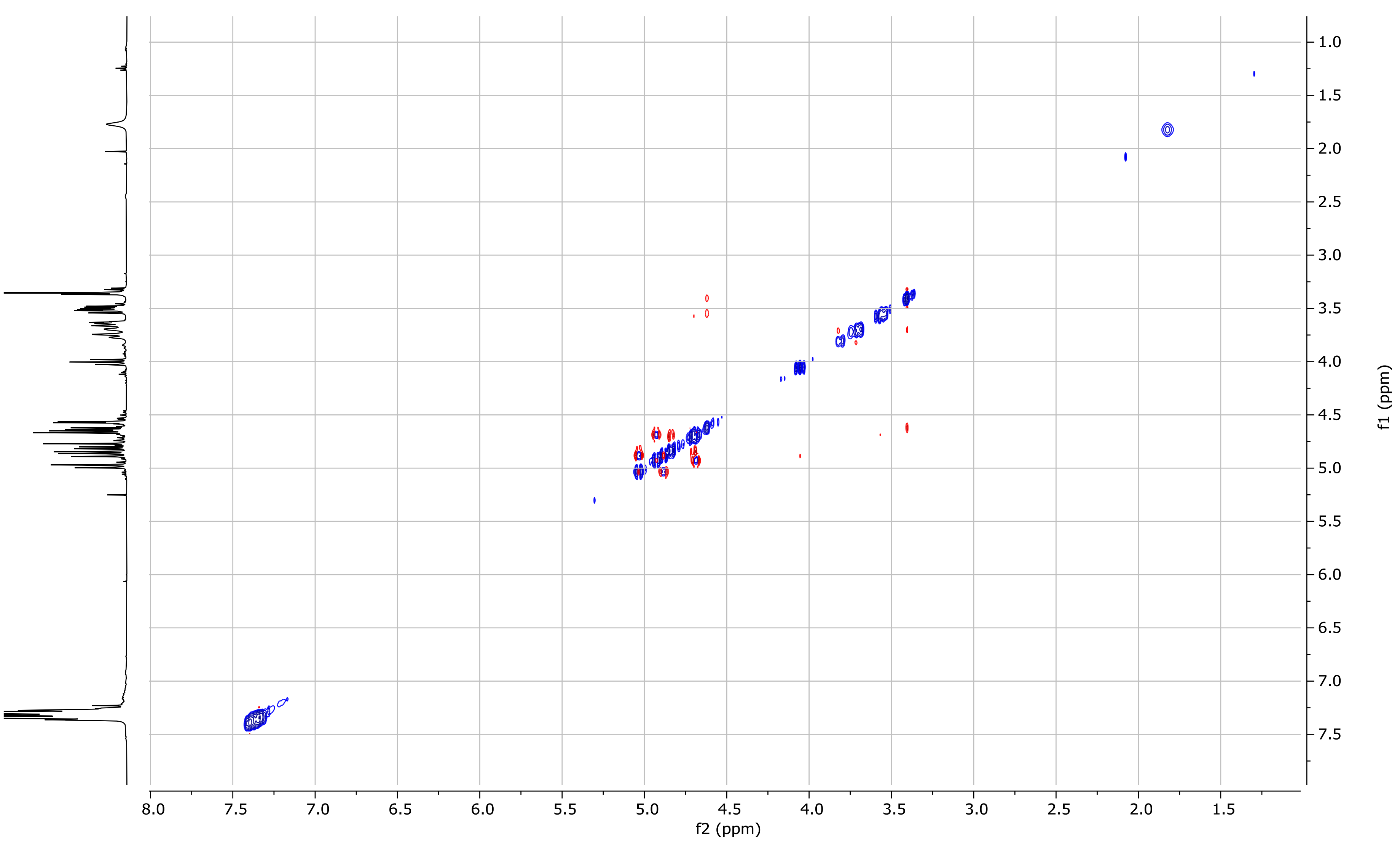

HSQC NMR spectrum of 1-*O*-methyl 2,3,4-tri-O-benzyl-α-D-*gluco*-pyranoside (**6**)

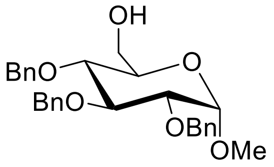

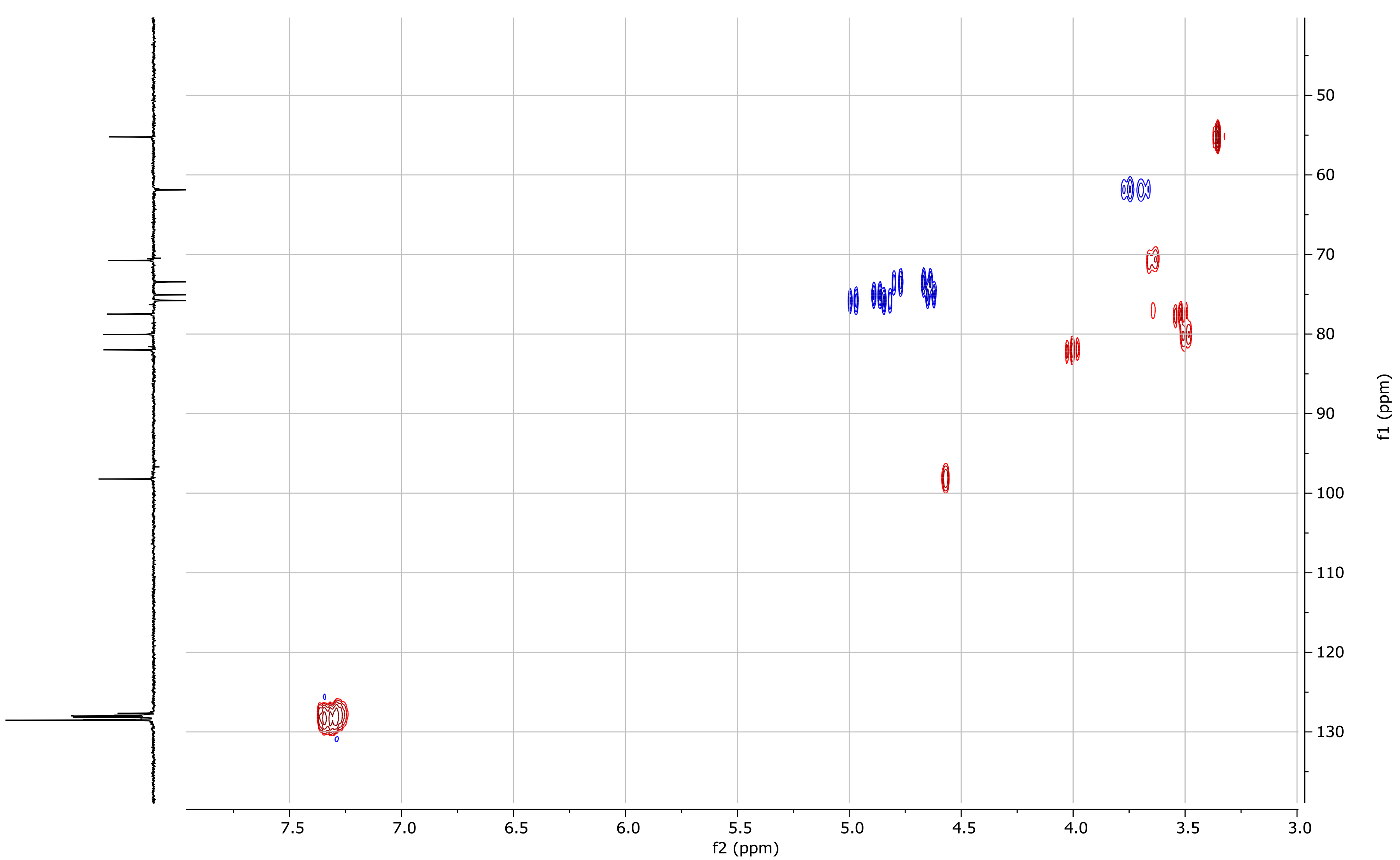

^{1}H NMR spectrum of 1-*O*-methyl 2,3,4-tri-*O*-benzyl-6-deoxy-α-D-*gluco*-hept-6-enopyranoside (**8**)

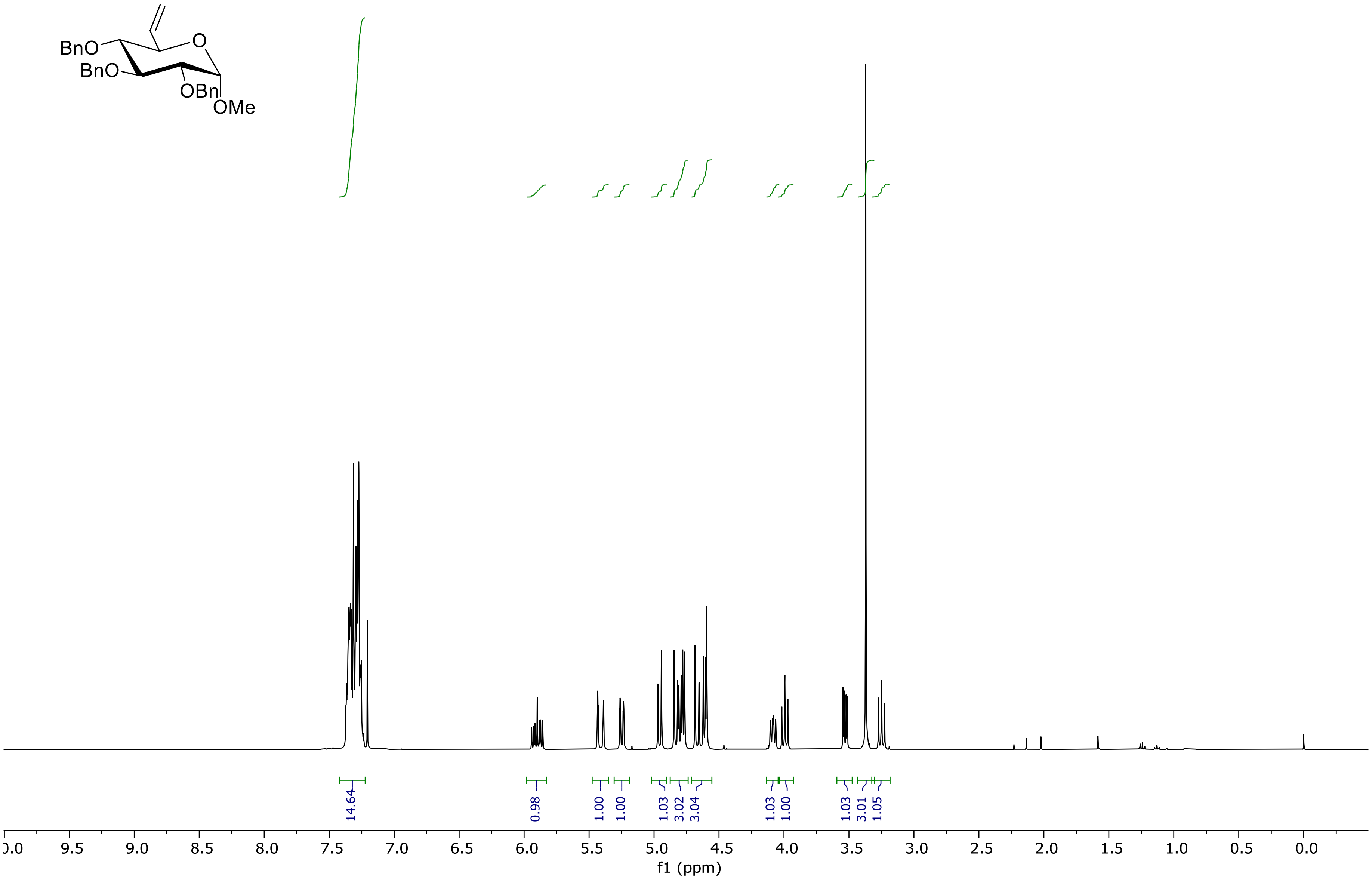

^{13}C NMR spectrum of 1-*O*-methyl 2,3,4-tri-*O*-benzyl-6-deoxy-α-D-*gluco*-hept-6-enopyranoside (**8**)

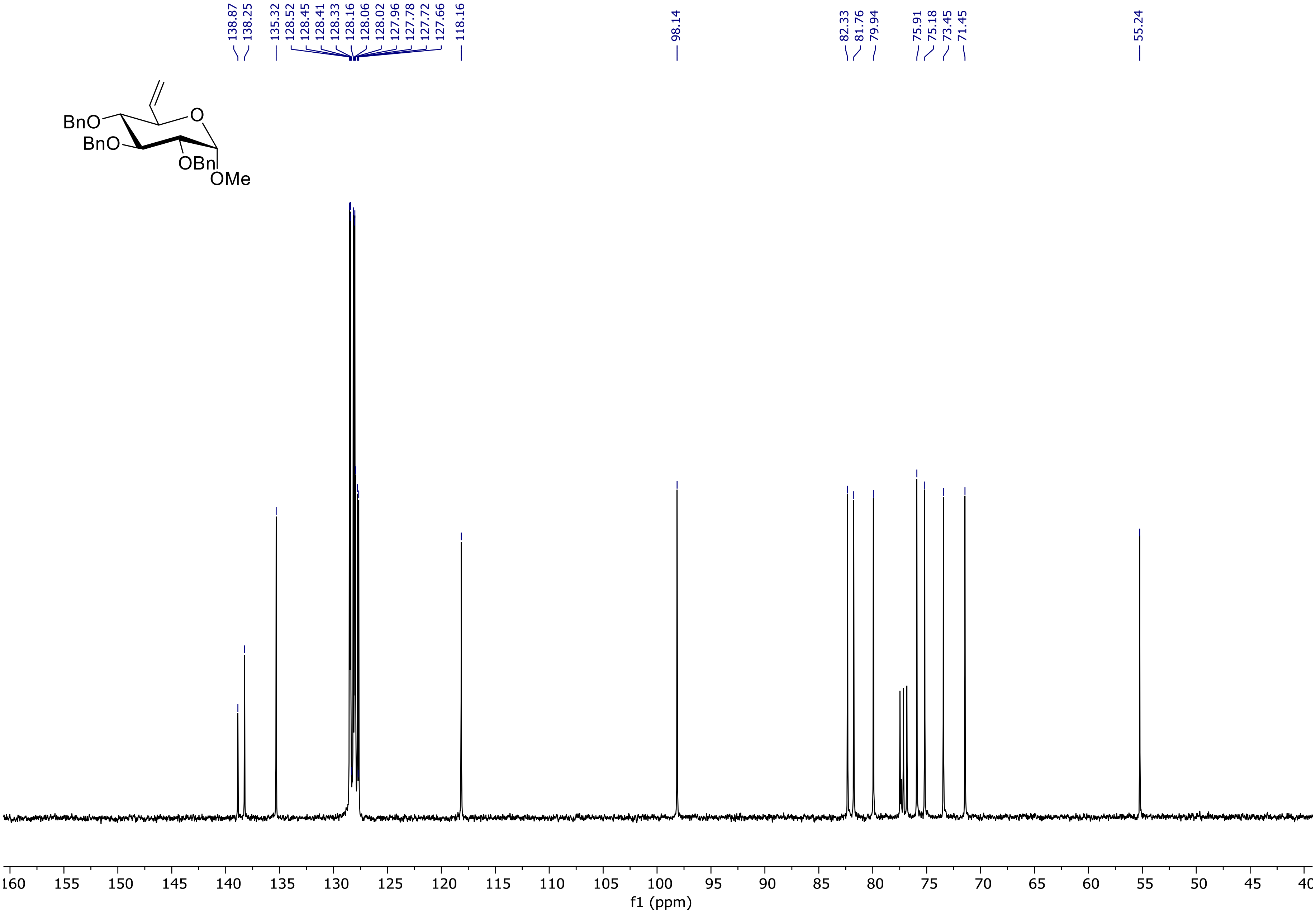

DEPT NMR spectrum of 1-*O*-methyl 2,3,4-tri-*O*-benzyl-6-deoxy-α-D-*gluco*-hept-6-enopyranoside (**8**)

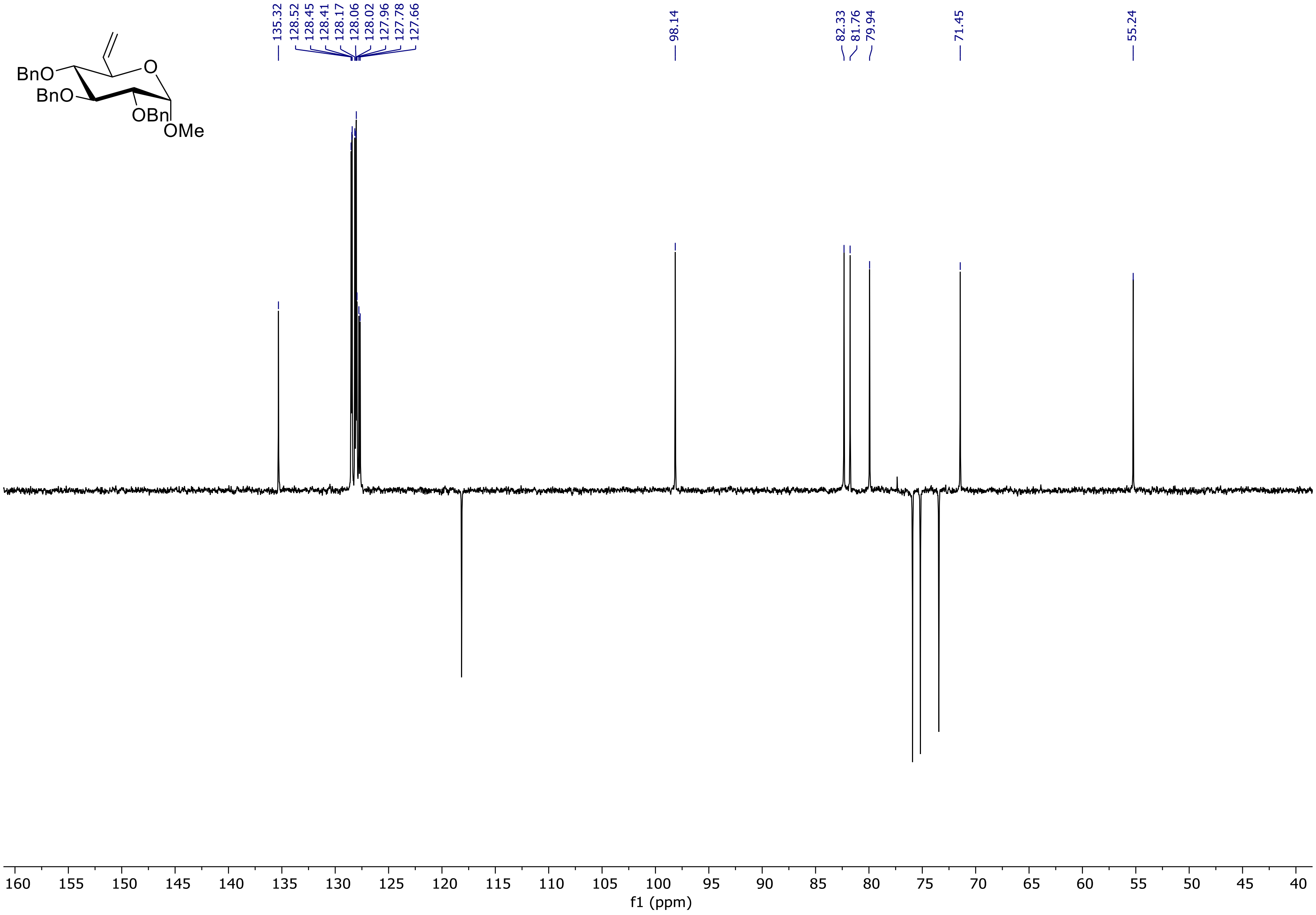

COSY NMR spectrum of 1-*O*-methyl 2,3,4-tri-*O*-benzyl-6-deoxy-α-D-*gluco*-hept-6-enopyranoside (**8**)

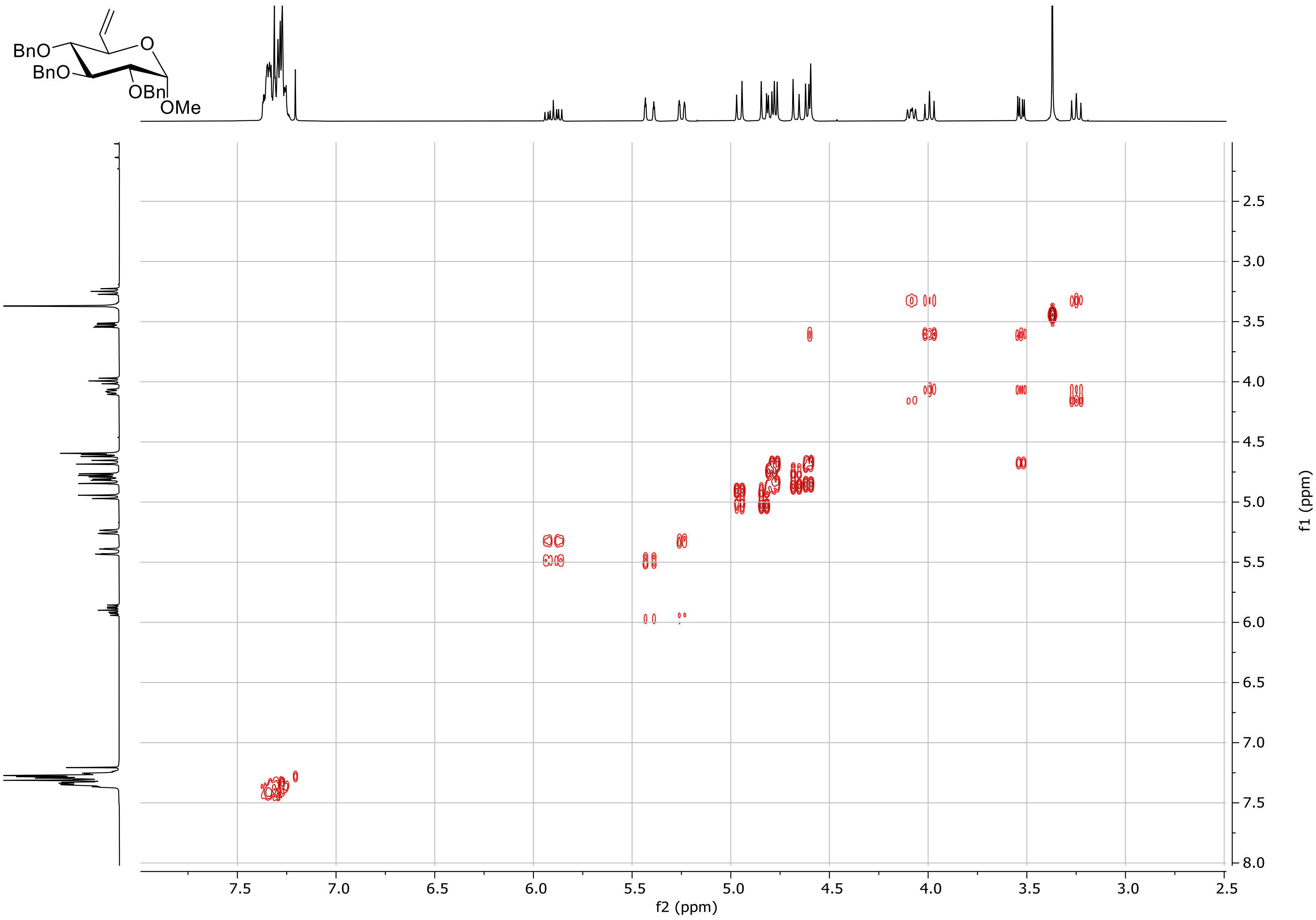

NOESY NMR spectrum of 1-*O*-methyl 2,3,4-tri-*O*-benzyl-6-deoxy-α-D-*gluco*-hept-6-enopyranoside (**8**)

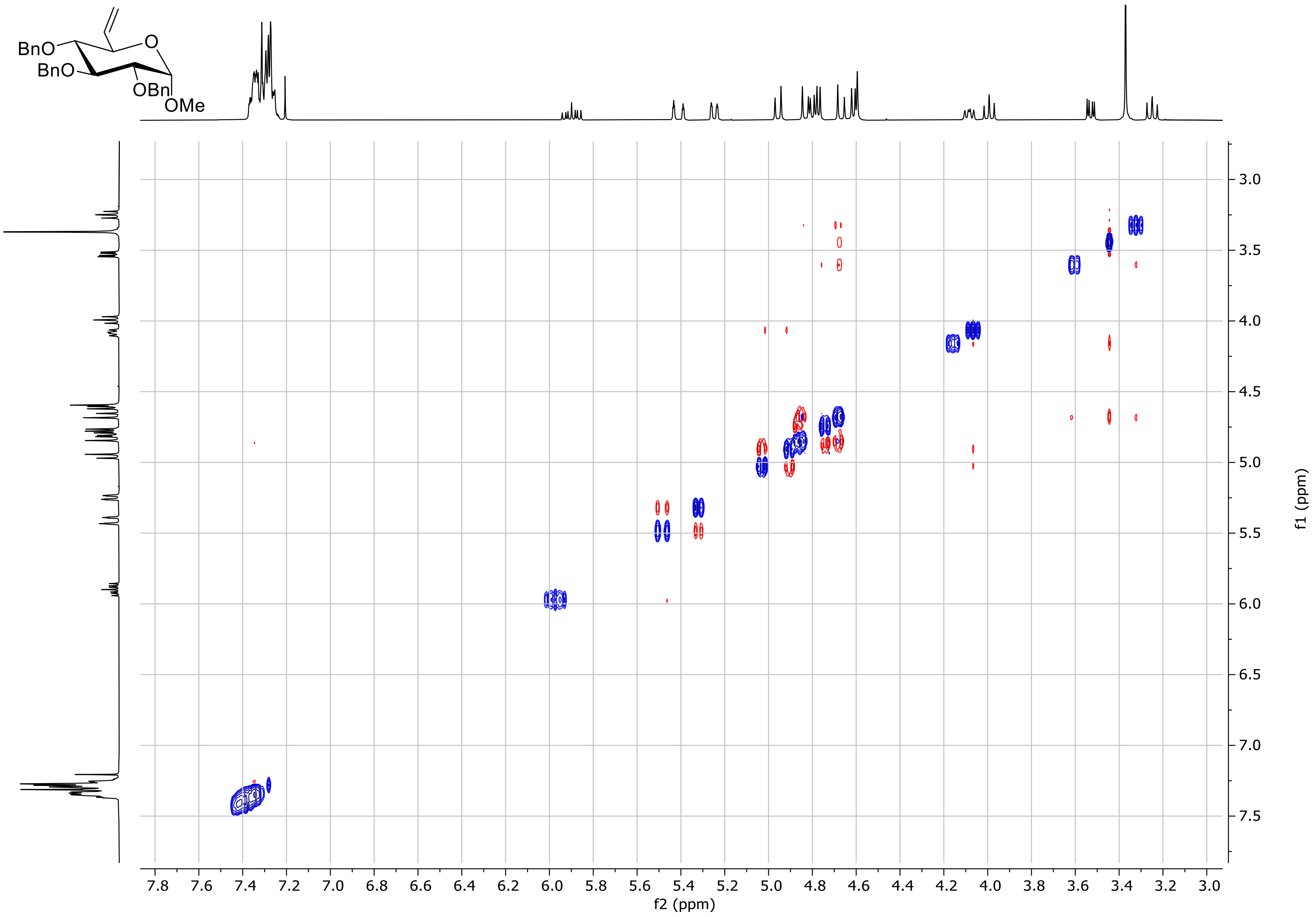

HSQC NMR spectrum of 1-*O*-methyl 2,3,4-tri-*O*-benzyl-6-deoxy-α-D-*gluco*-hept-6-enopyranoside (**8**)

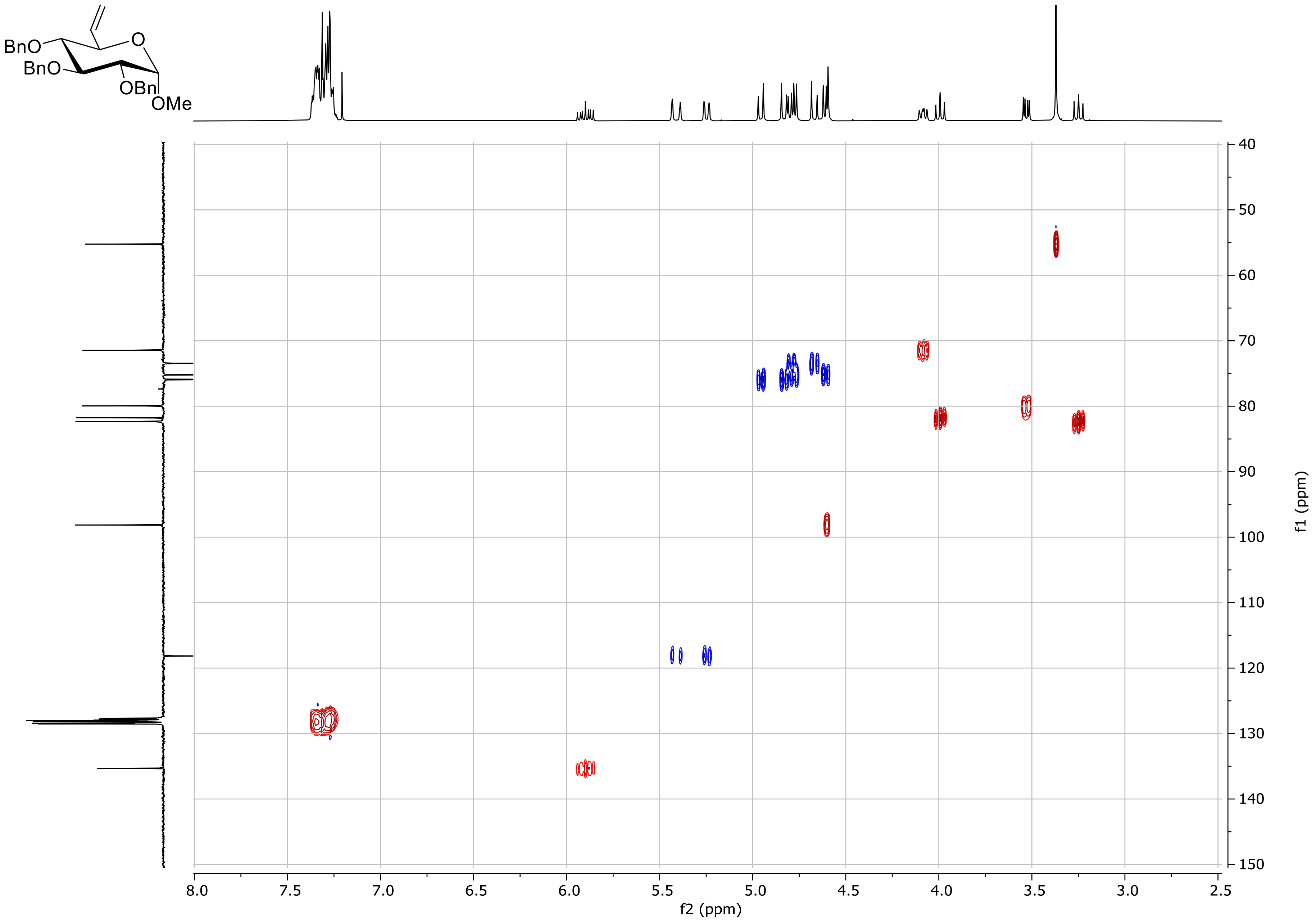

^{1}H NMR spectrum of 1-*O*-methyl2,3,4-tri-*O*-benzyl-D/L-*glycero*-α-D-*gluco*-heptopyranoside (**9**)

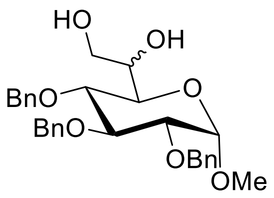

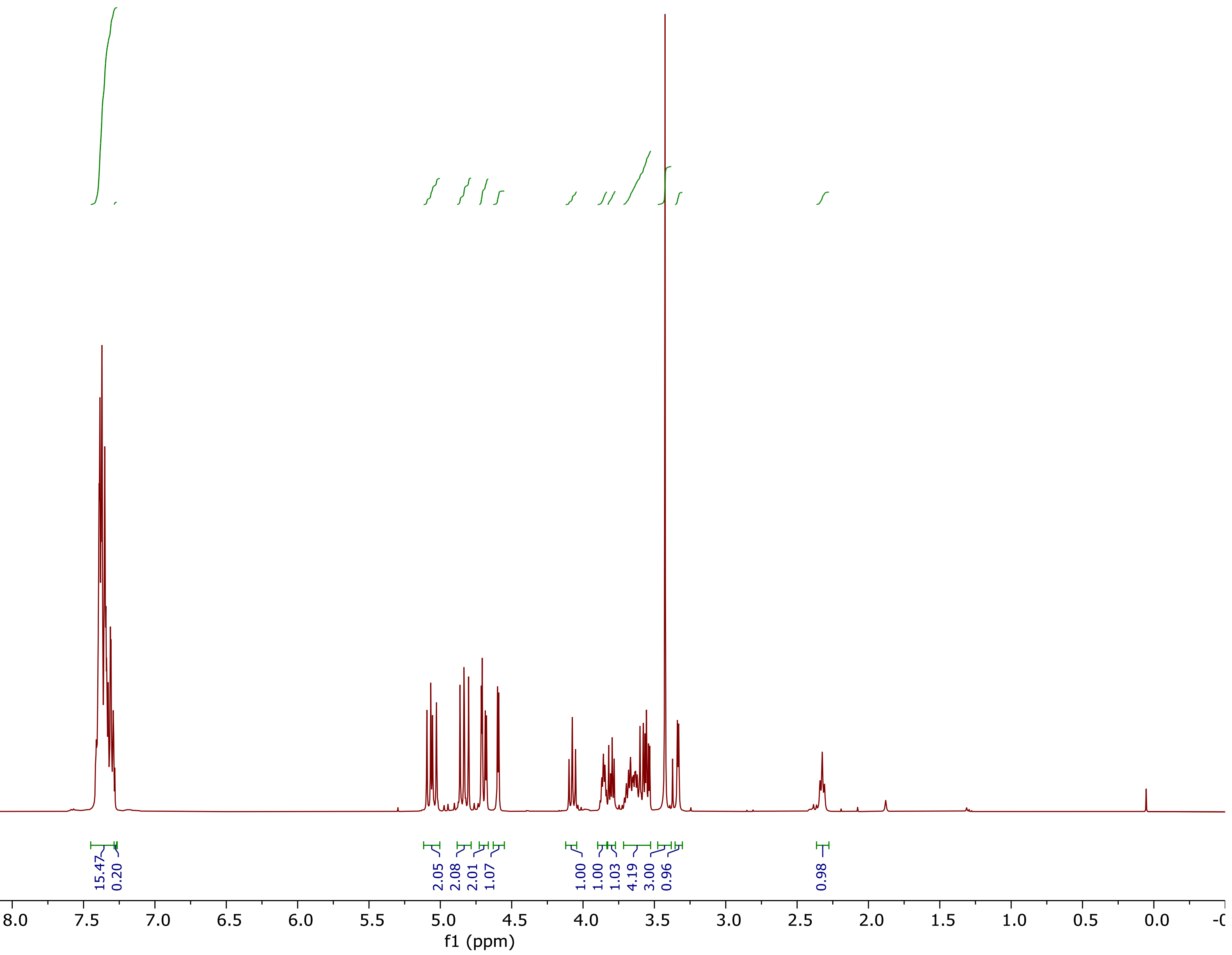

^{13}C NMR spectrum of 1-*O*-methyl 2,3,4-tri-*O*-benzyl-D/L-*glycero*-α-D-*gluco*-heptopyranoside (**9**)

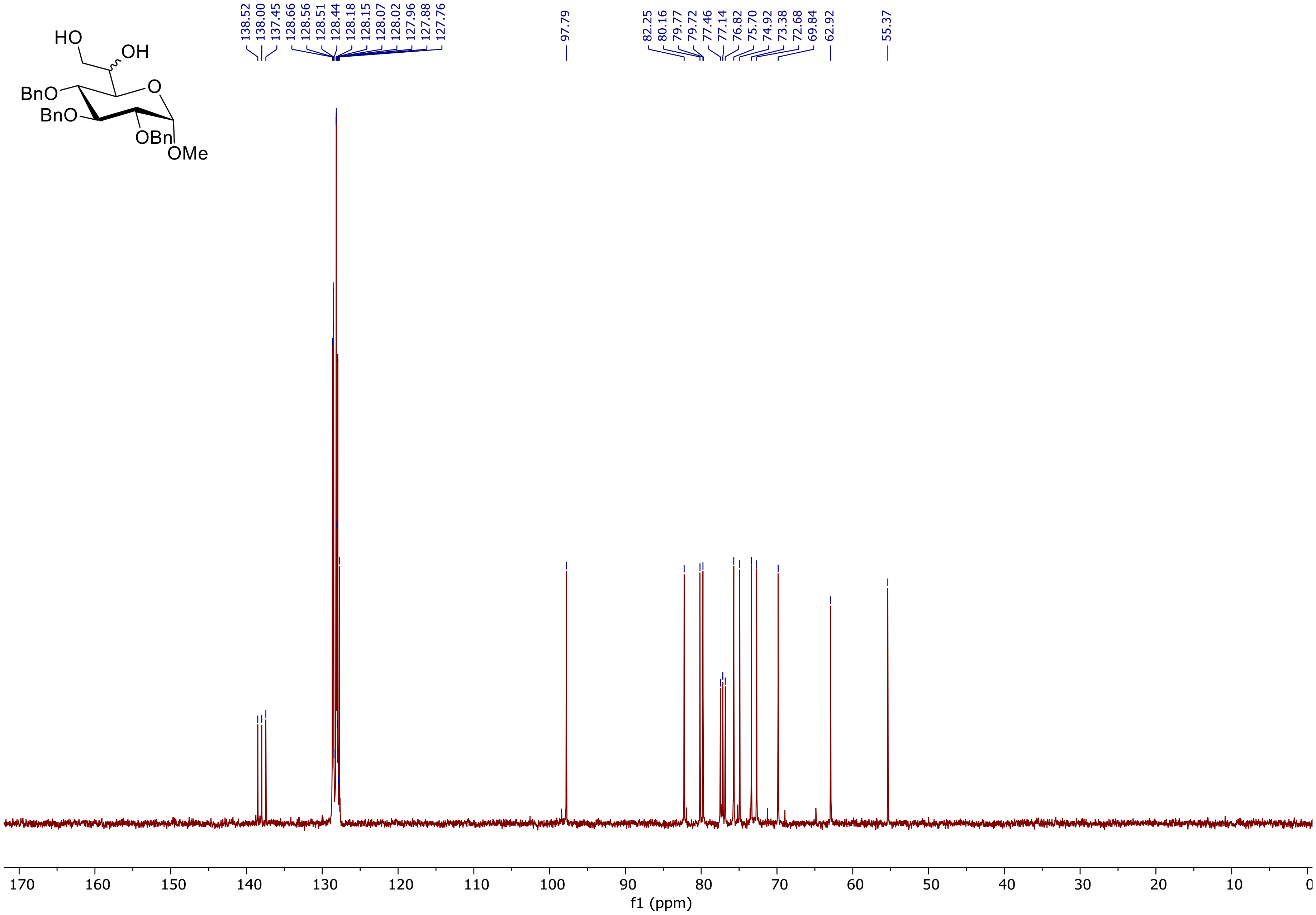

DEPT NMR spectrum of 1-*O*-methyl 2,3,4-tri-*O*-benzyl-D/L-*glycero*-α-D-*gluco*-heptopyranoside (**9**)

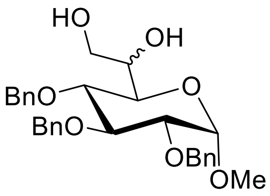

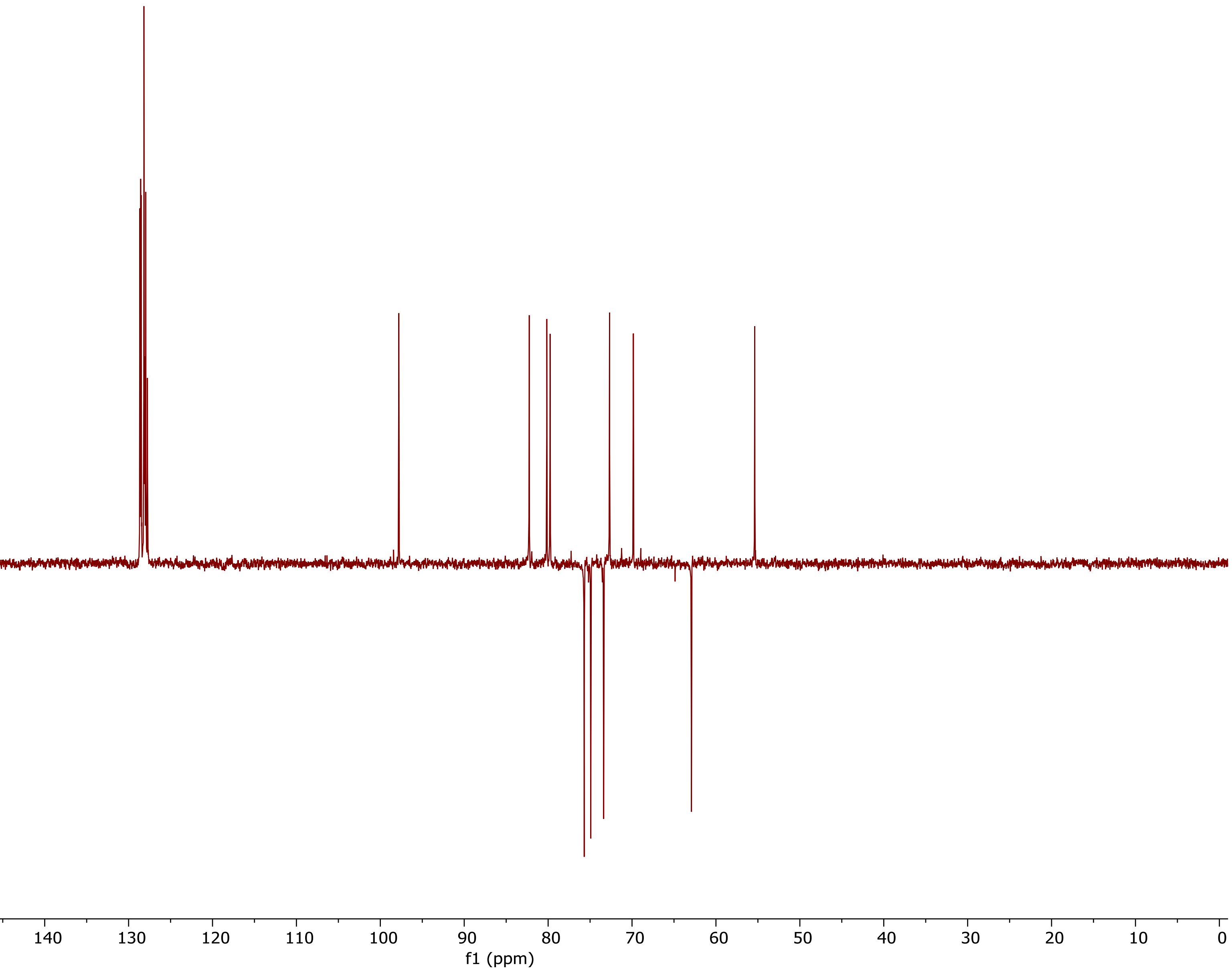

COSY NMR spectrum of 1-*O*-methyl 2,3,4-tri-*O*-benzyl-D/L-*glycero*-α-D-*gluco*-heptopyranoside (**9**)

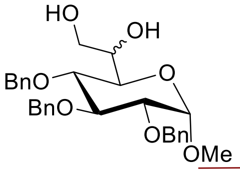

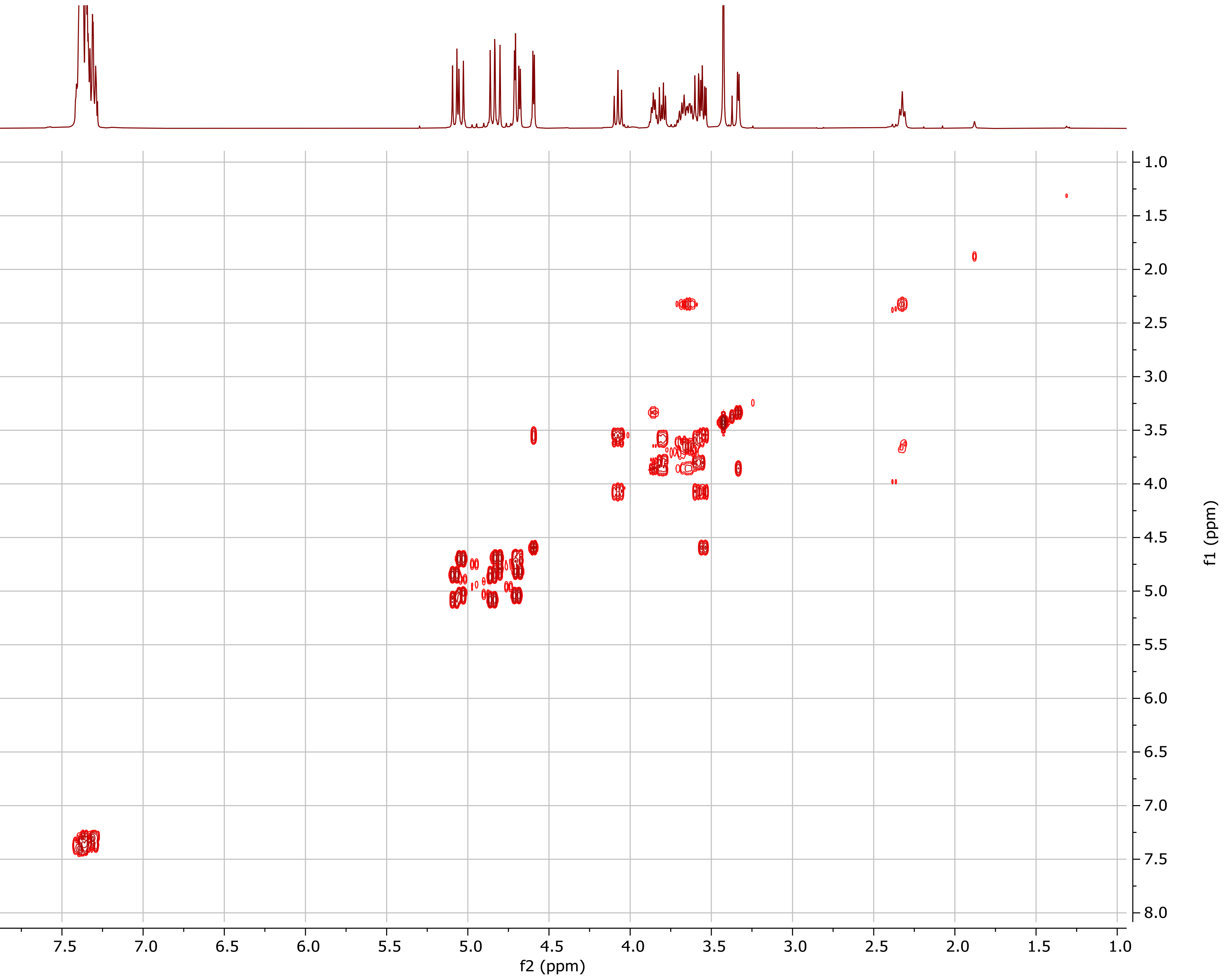

NOESY NMR spectrum of 1-*O*-methyl 2,3,4-tri-*O*-benzyl-D/L-*glycero*-α-D-*gluco*-heptopyranoside (**9**)

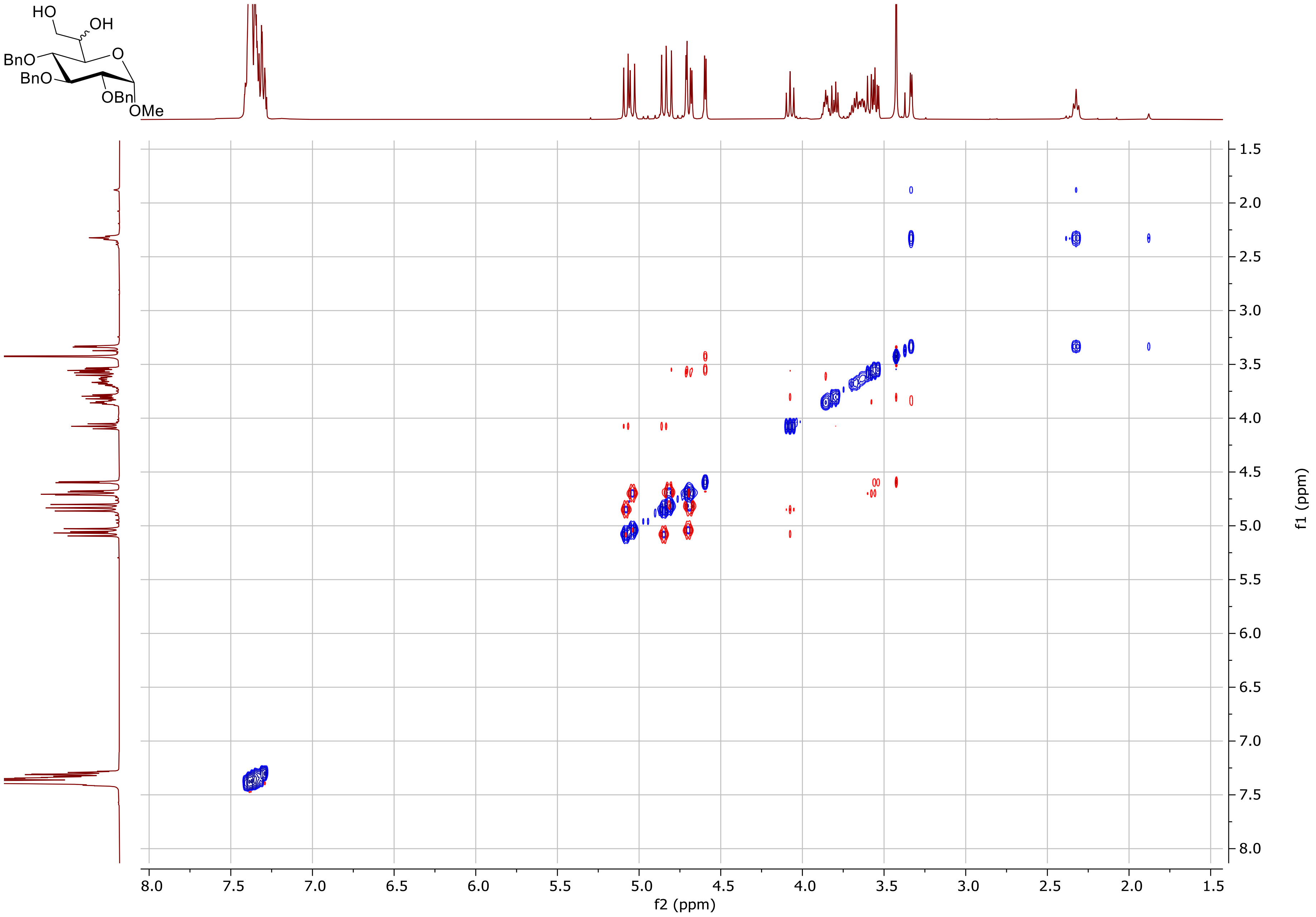

HSQC NMR spectrum of 1-*O*-methyl 2,3,4-tri-*O*-benzyl-D/L-*glycero*-α-D-*gluco*-heptopyranoside (**9**)

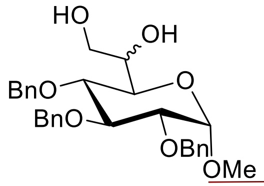

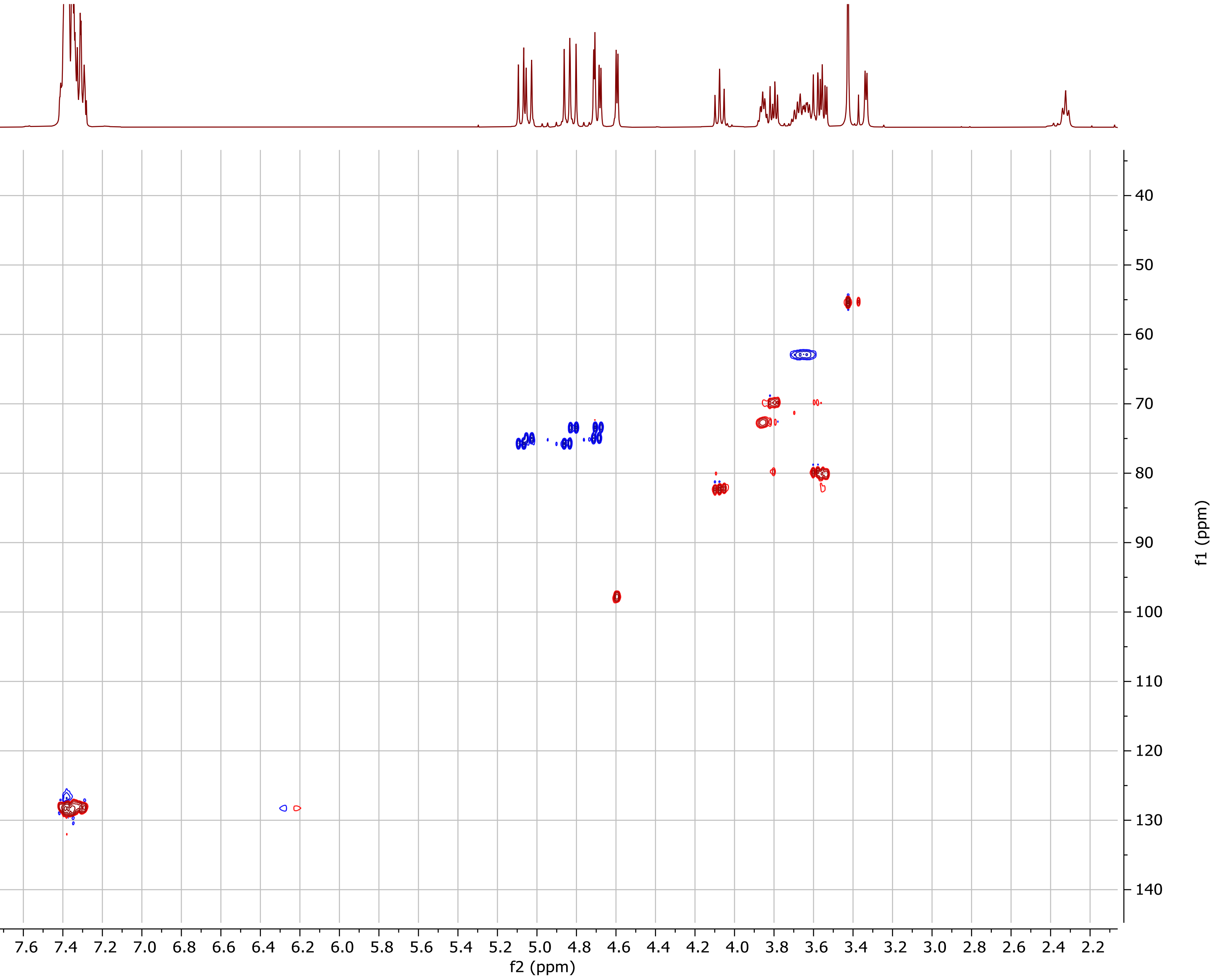

^{1}H NMR spectrum of 1-*O*-methyl 2,3,4,6-tetra-*O*-benzyl-D-*glycero*-α-D-*gluco*-heptopyranoside (**12**)

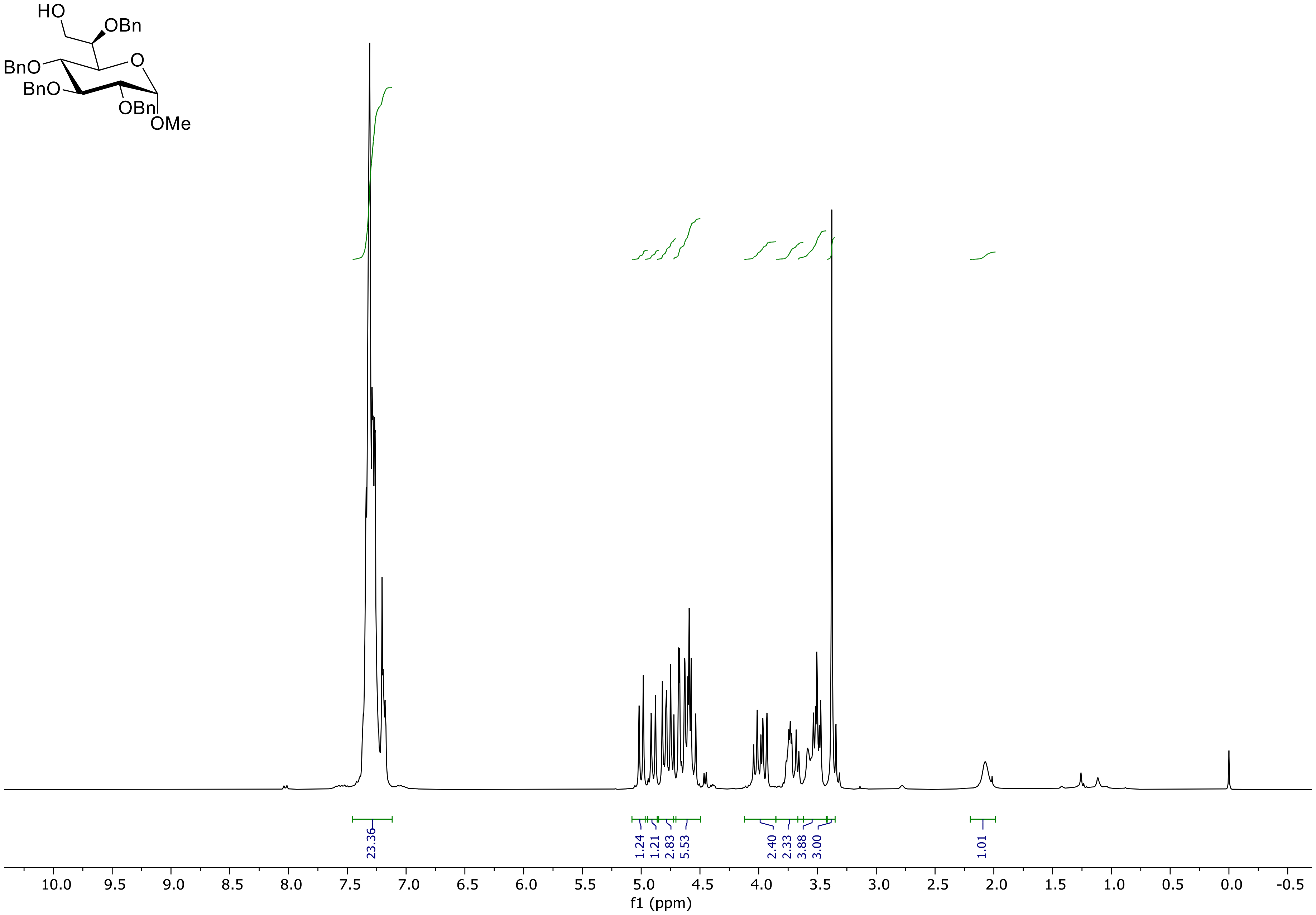

^{13}C NMR spectrum of 1-*O*-methyl 2,3,4,6-tetra-*O*-benzyl-D-*glycero*-α-D-*gluco*-heptopyranoside (**12**)

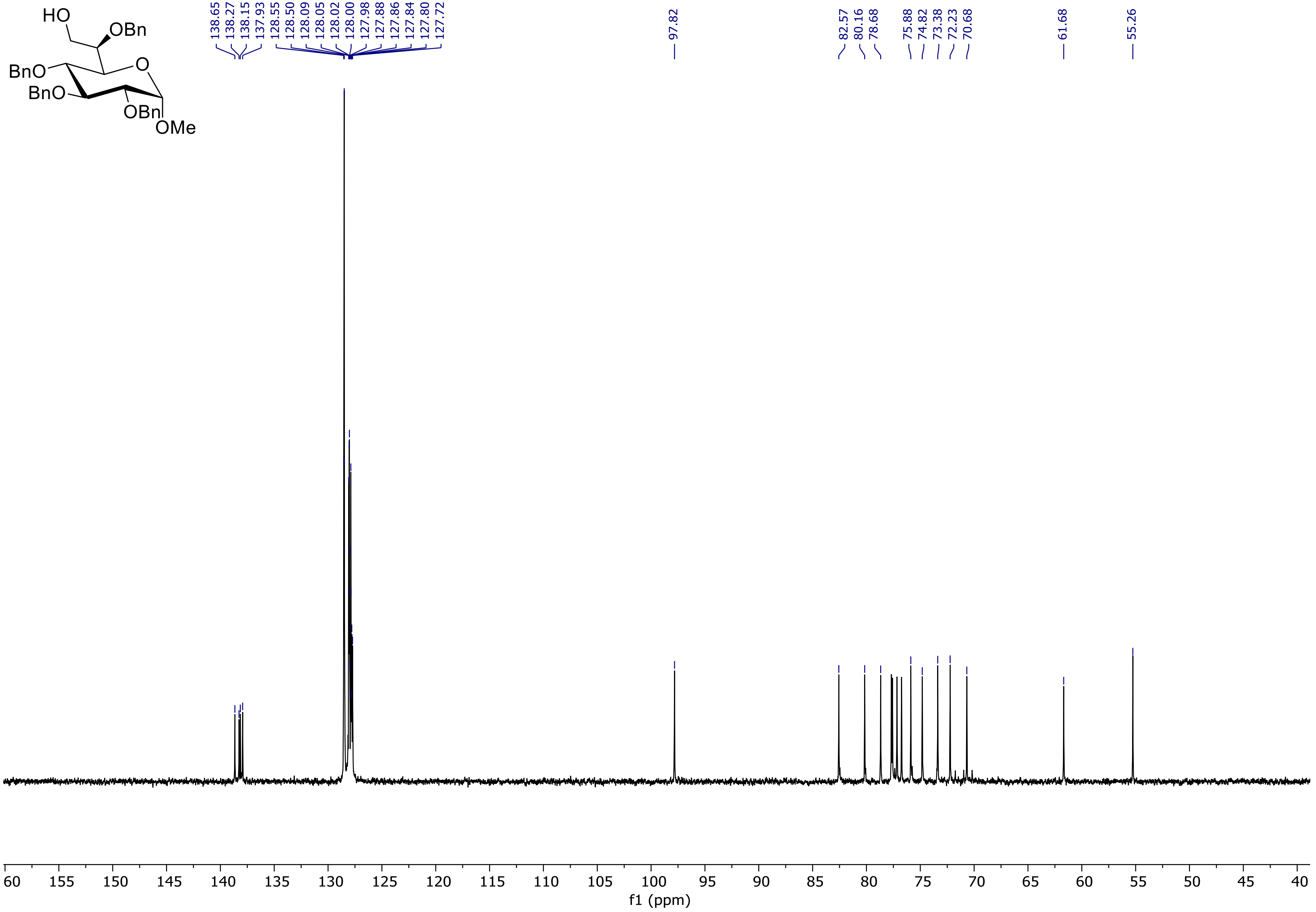

DEPT NMR spectrum of 1-*O*-methyl 2,3,4,6-tetra-*O*-benzyl-D-*glycero*-α-D-*gluco*-heptopyranoside (**12**)

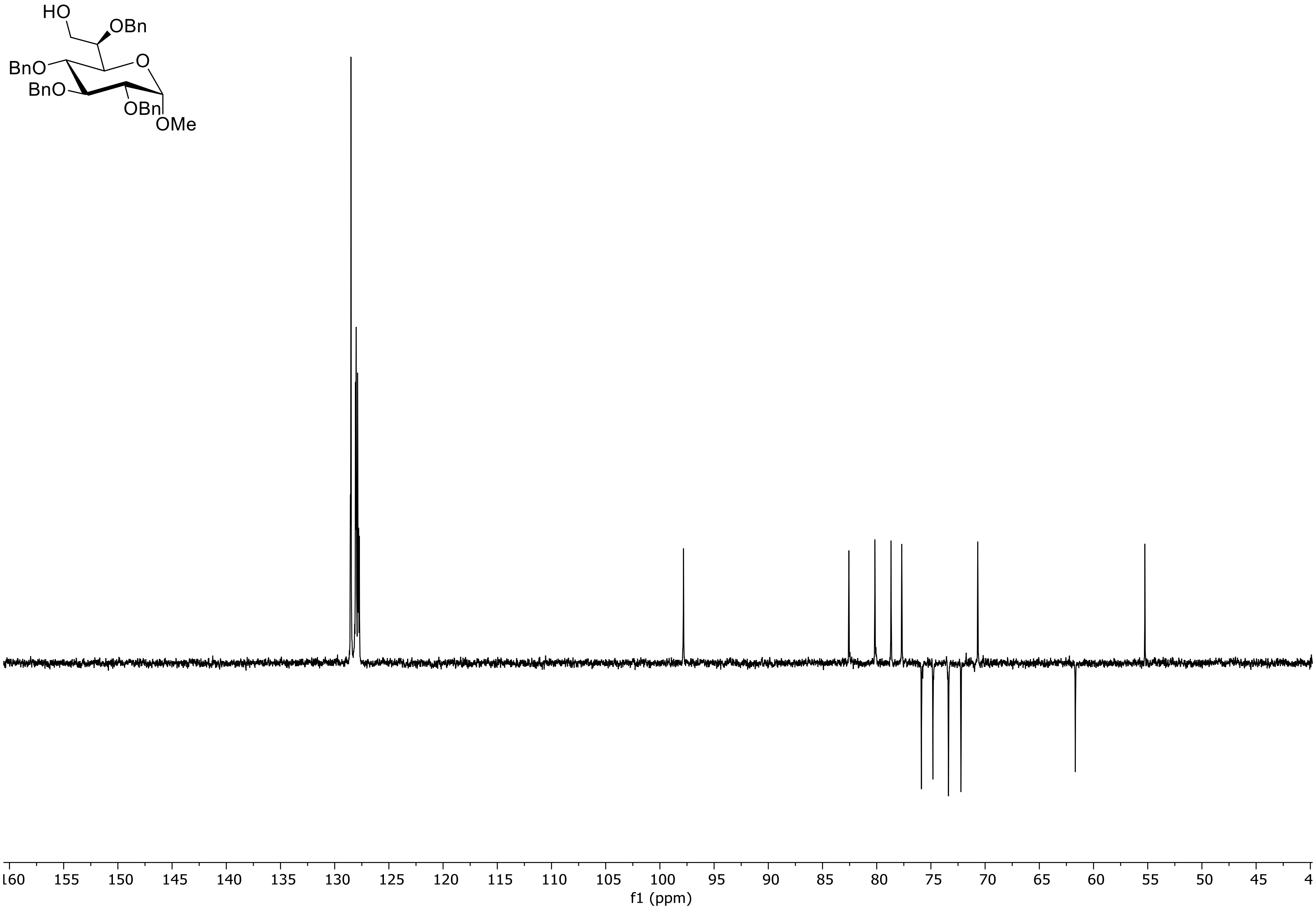

COSY NMR spectrum of 1-*O*-methyl 2,3,4,6-tetra-*O*-benzyl-D-*glycero*-α-D-*gluco*-heptopyranoside (**12**)

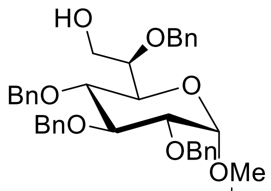

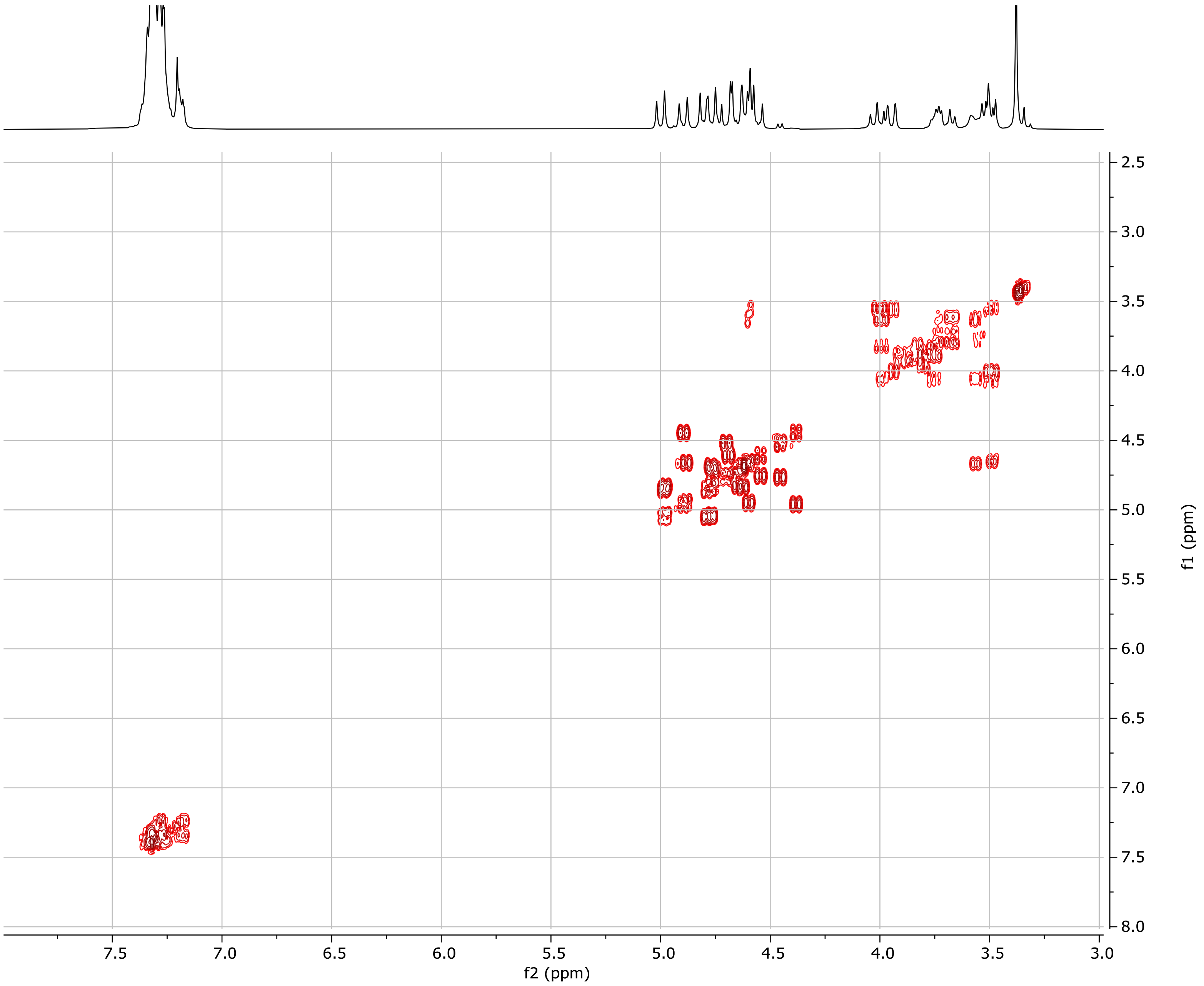

HSQC NMR spectrum of 1-*O*-methyl 2,3,4,6-tetra-*O*-benzyl-D-*glycero*-α-D-*gluco*-heptopyranoside (**12**)

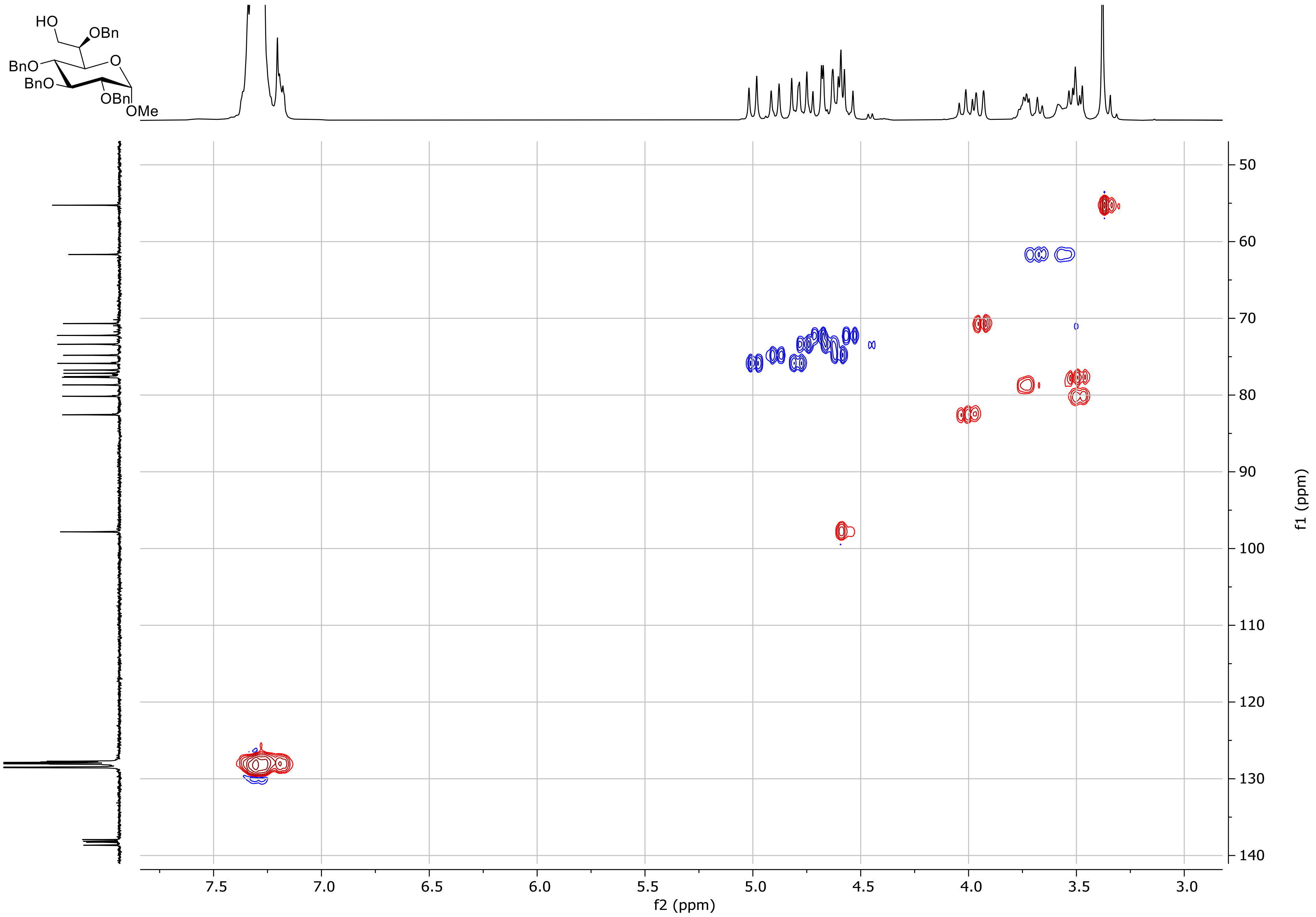

^{1}H NMR spectrum of 1-*O*-methyl 2,3,4,6-tetra-*O*-benzyl-D-*glycero*-α-D-*gluco*-heptopyranoside 7-(dibenzyl)phosphate (**13**)

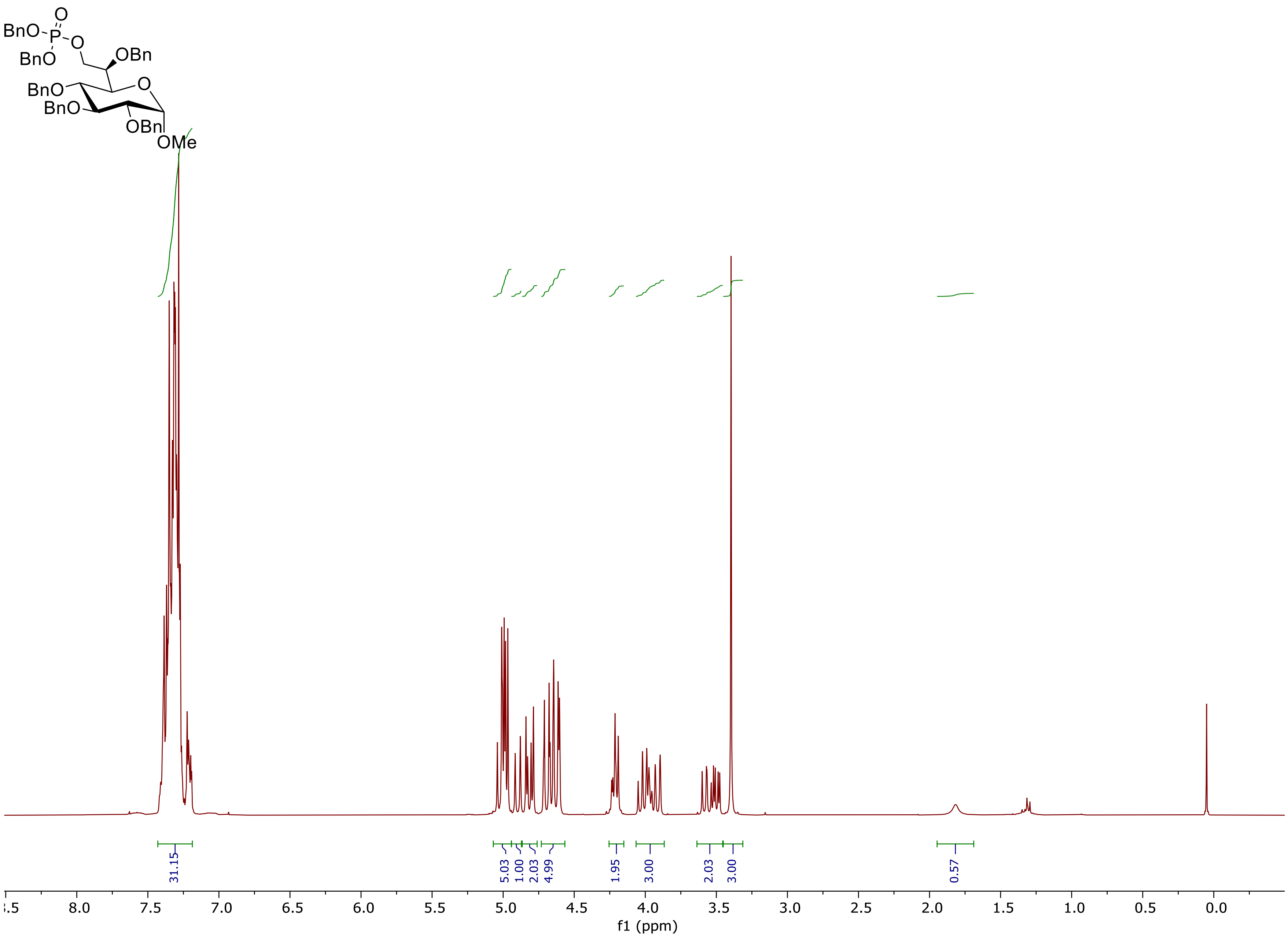

^{13}C NMR spectrum of 1-*O*-methyl 2,3,4,6-tetra-*O*-benzyl-D-*glycero*-α-D-*gluco*-heptopyranoside 7-(dibenzyl)phosphate (**13**)

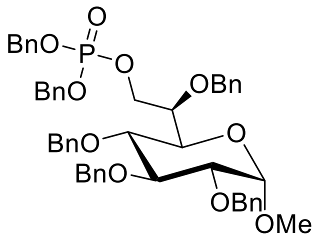

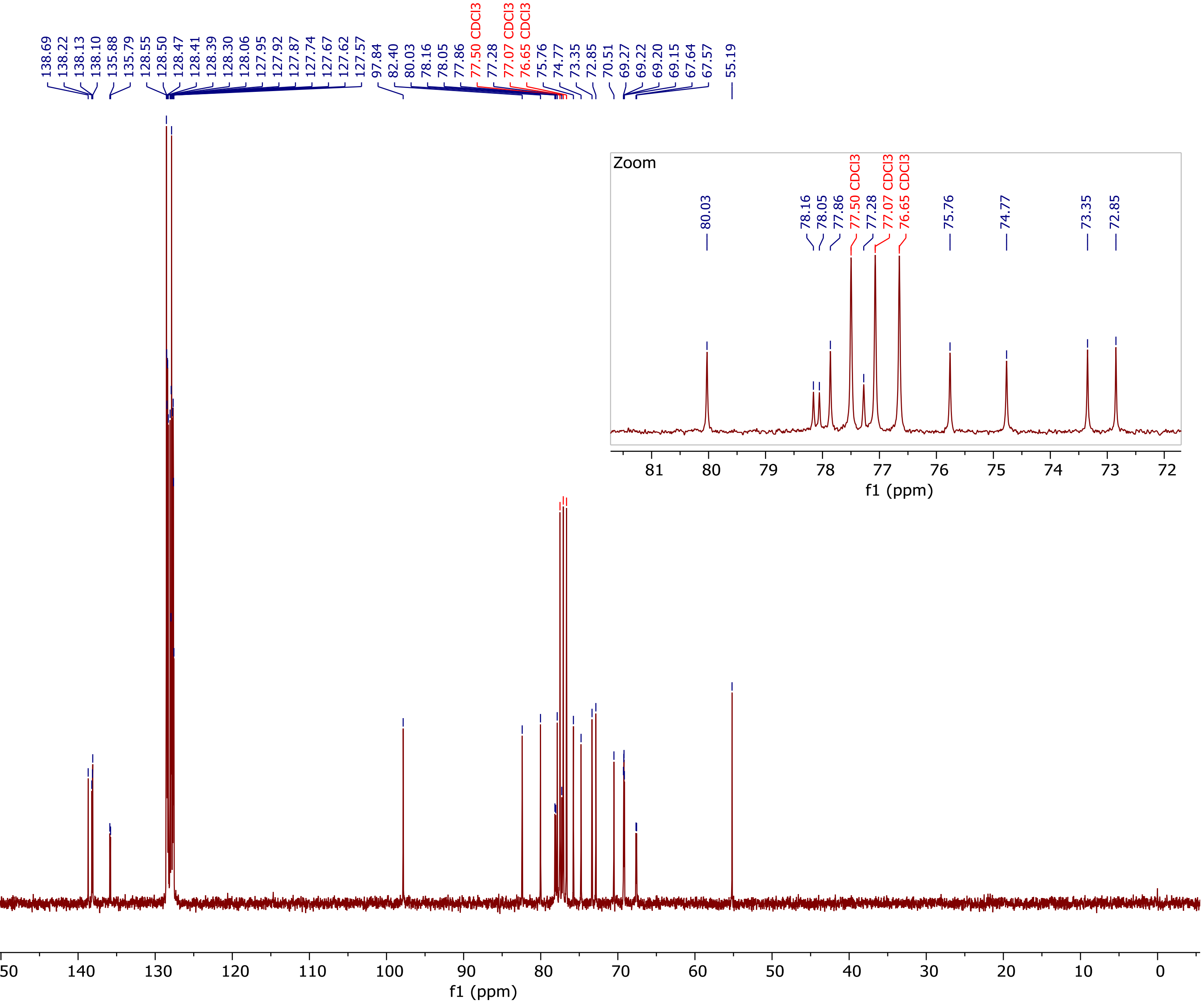

DEPT NMR spectrum of 1-*O*-methyl 2,3,4,6-tetra-*O*-benzyl-D-*glycero*-α-D-*gluco*-heptopyranoside 7-(dibenzyl)phosphate (**13**)

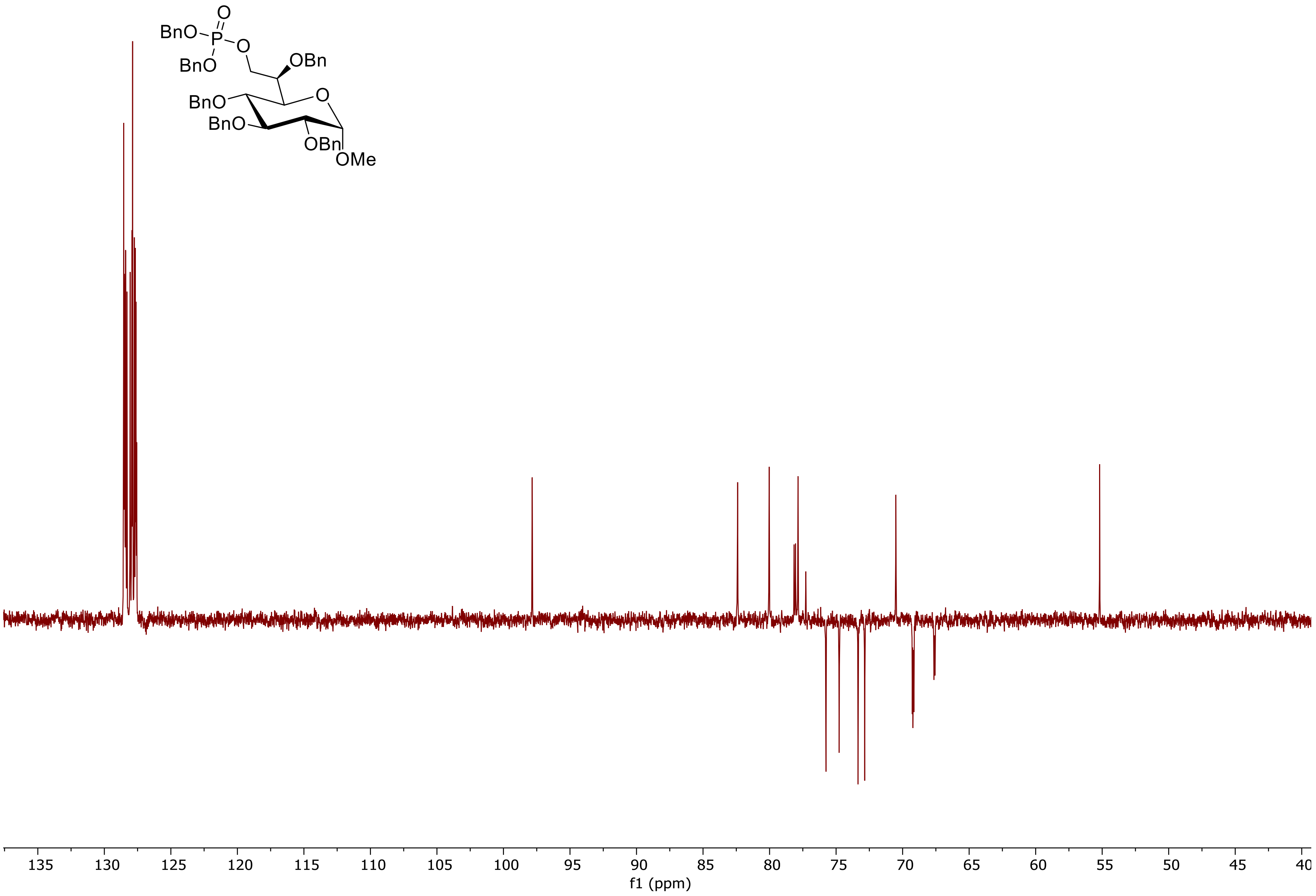

^{31}P NMR spectrum of 1-*O*-methyl 2,3,4,6-tetra-*O*-benzyl-D-*glycero*-α-D-*gluco*-heptopyranoside 7-(dibenzyl)phosphate (**13**)

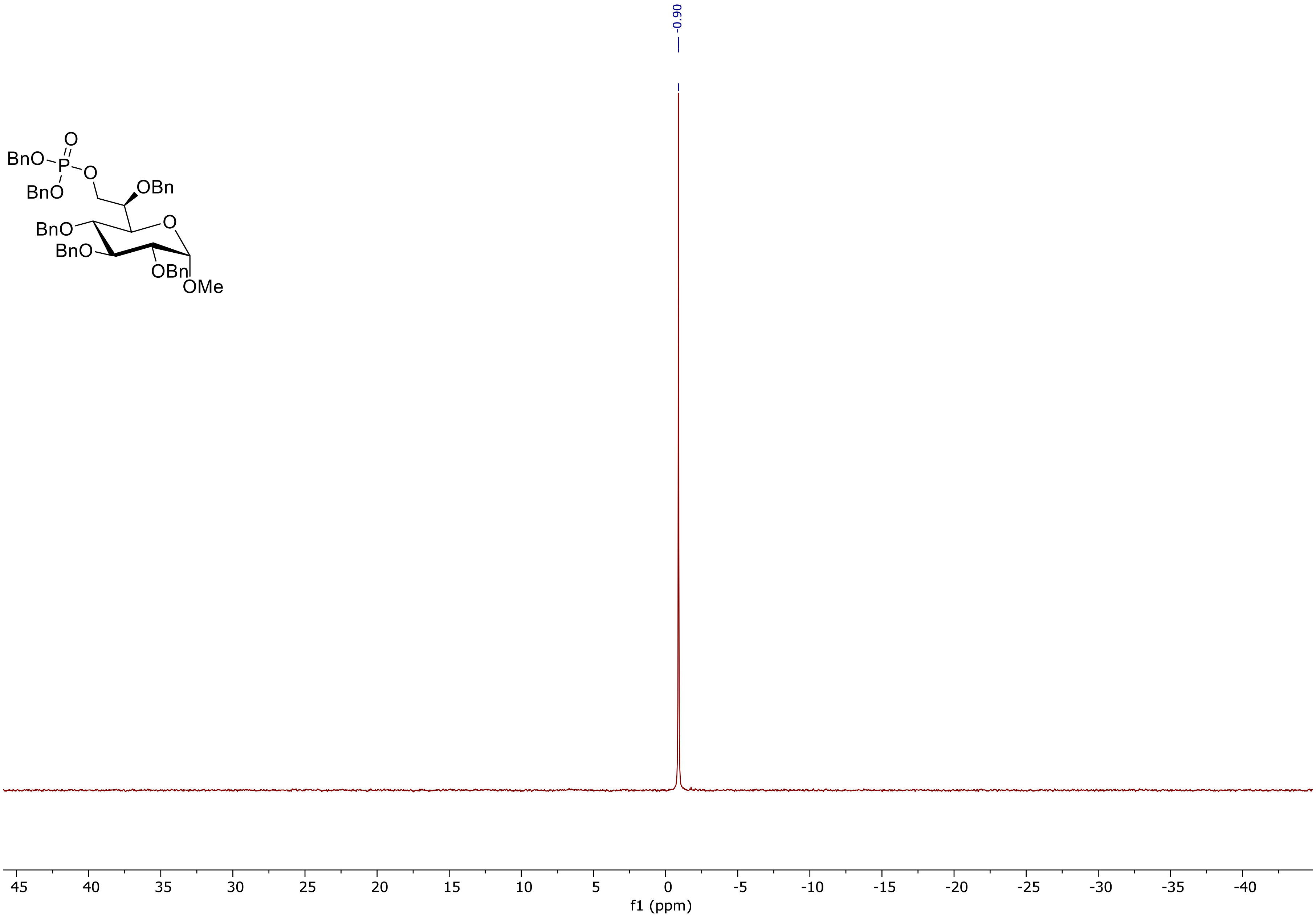

COSY NMR spectrum of 1-*O*-methyl 2,3,4,6-tetra-*O*-benzyl-D-*glycero*-α-D-*gluco*-heptopyranoside 7-(dibenzyl)phosphate (**13**)

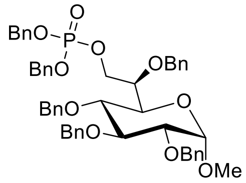

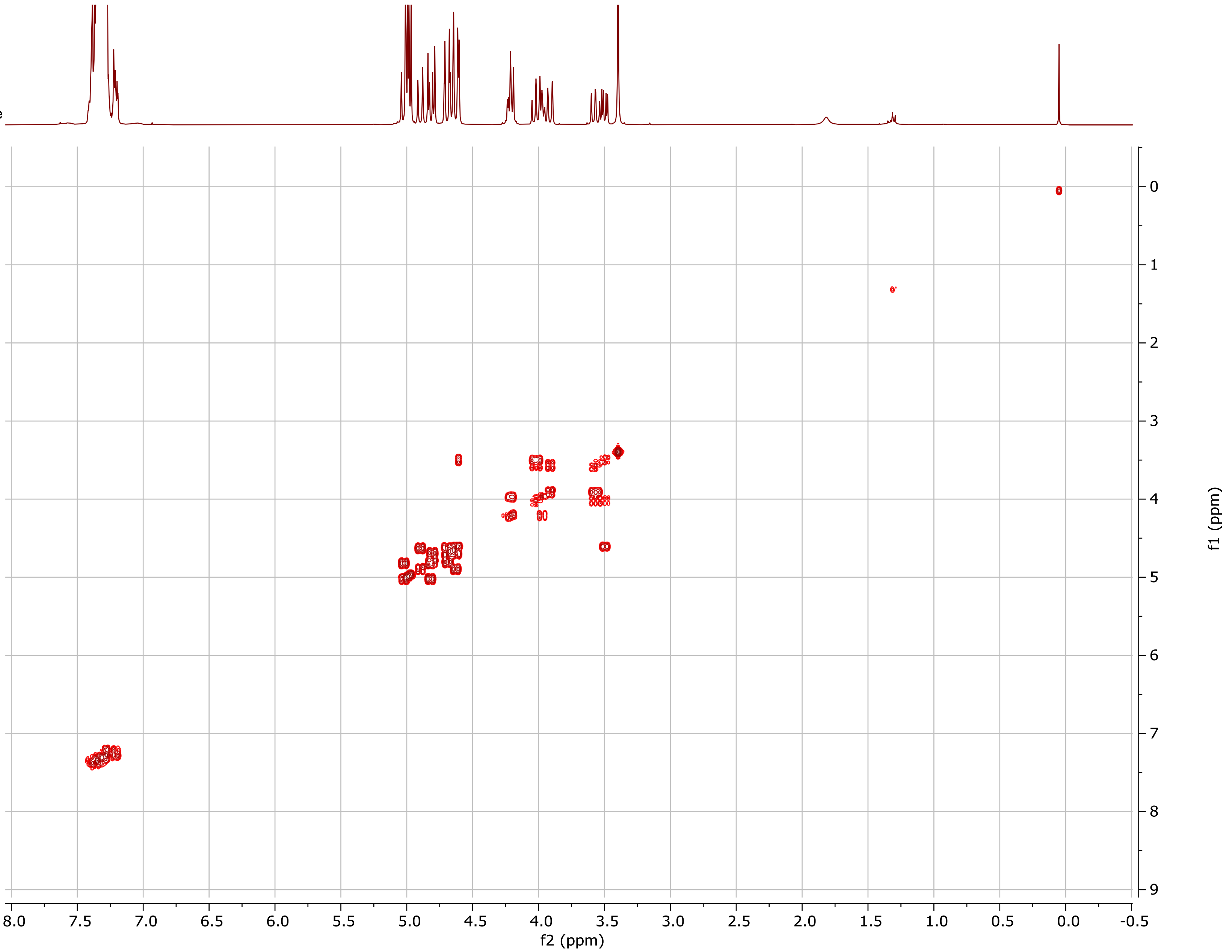

NOESY NMR spectrum of 1-*O*-methyl 2,3,4,6-tetra-*O*-benzyl-D-*glycero*-α-D-*gluco*-heptopyranoside 7-(dibenzyl)phosphate (**13**)

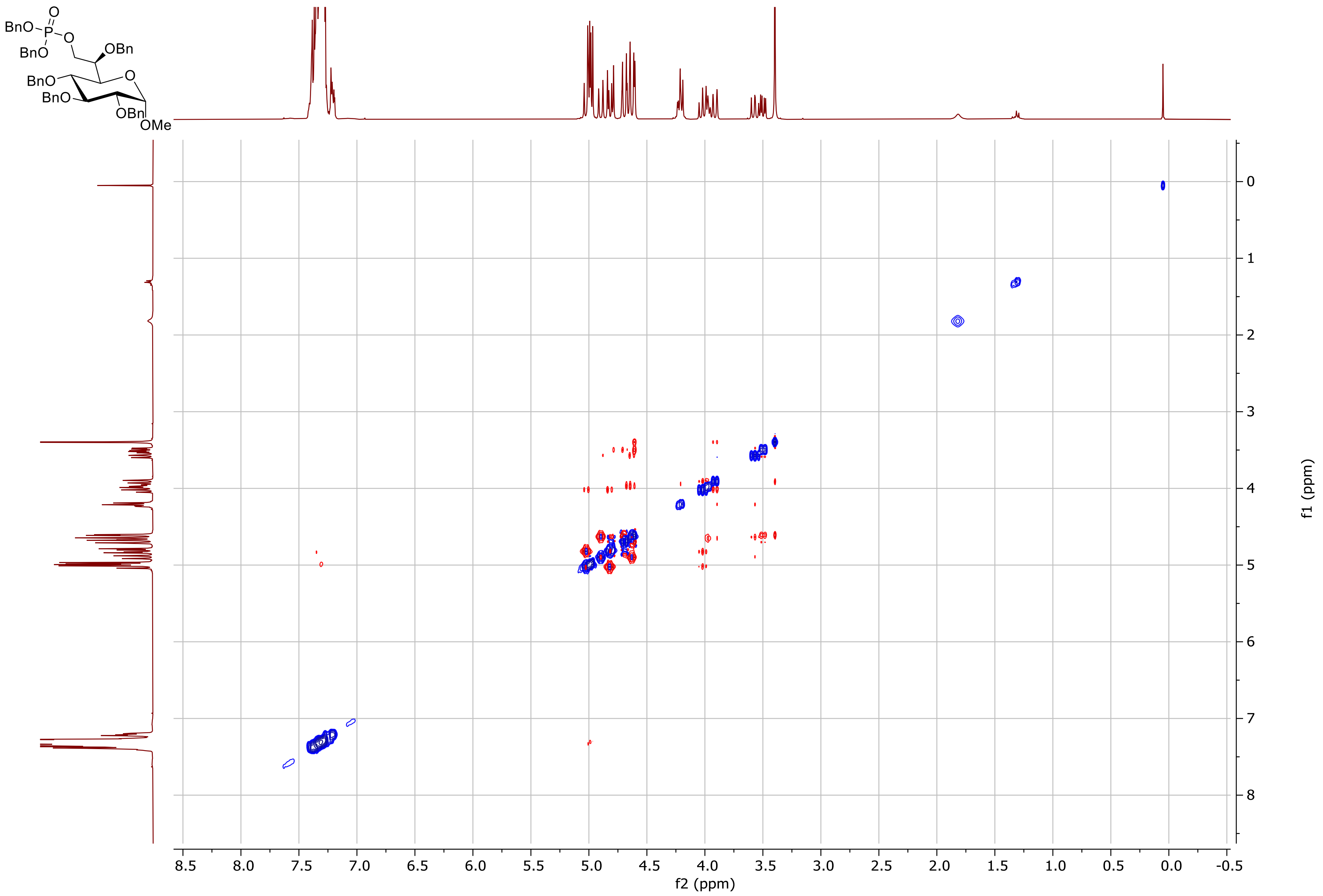

HSQC NMR spectrum of 1-*O*-methyl 2,3,4,6-tetra-*O*-benzyl-D-*glycero*-α-D-*gluco*-heptopyranoside 7-(dibenzyl)phosphate (**13**)

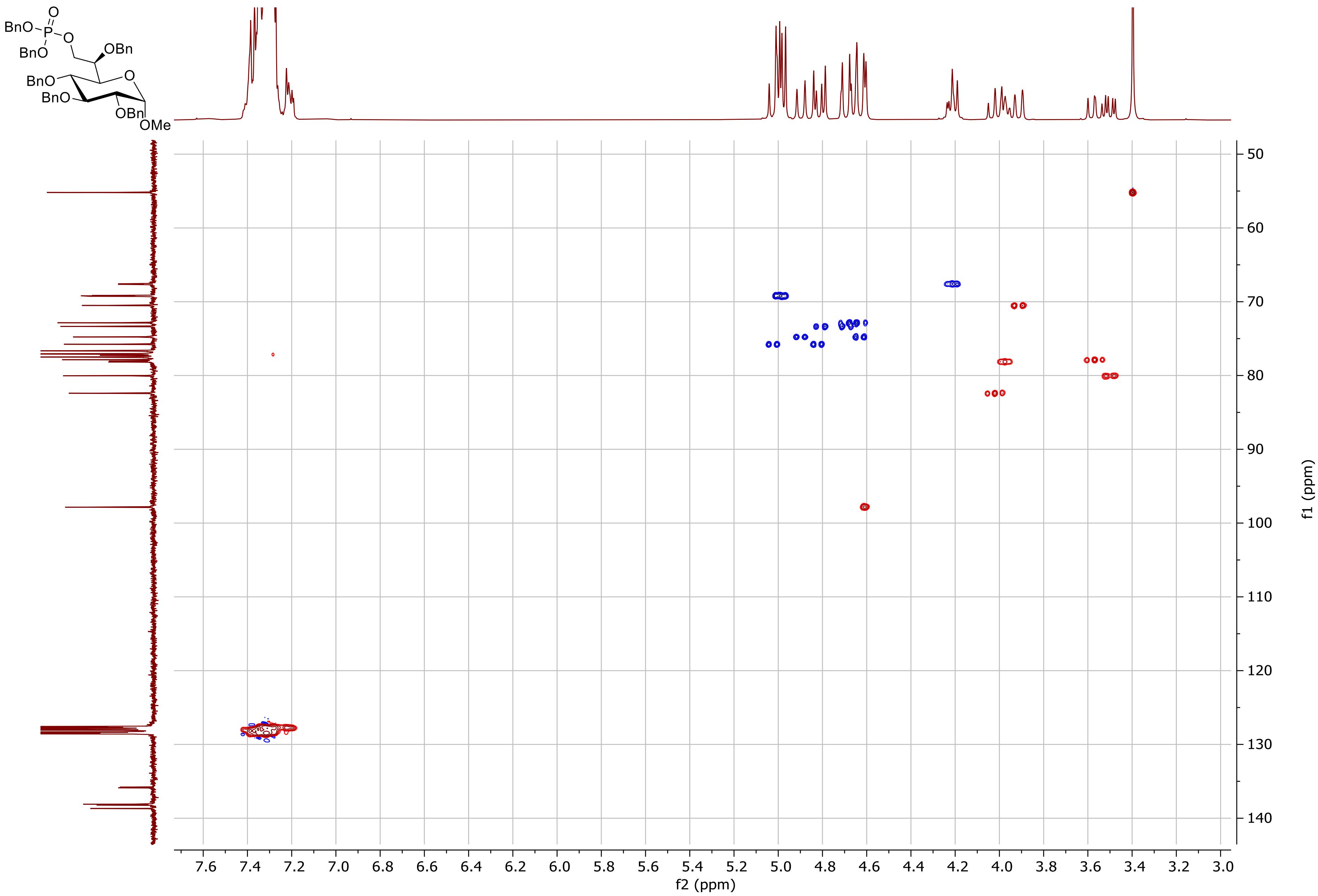

^{31}P-HMBC NMR spectrum of 1-O-methyl 2,3,4,6-tetra-O-benzyl-D-glycero-α-D-gluco-heptopyranoside 7-(dibenzyl)phosphate (**13**)

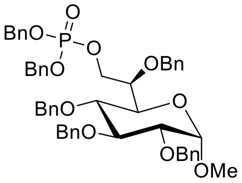

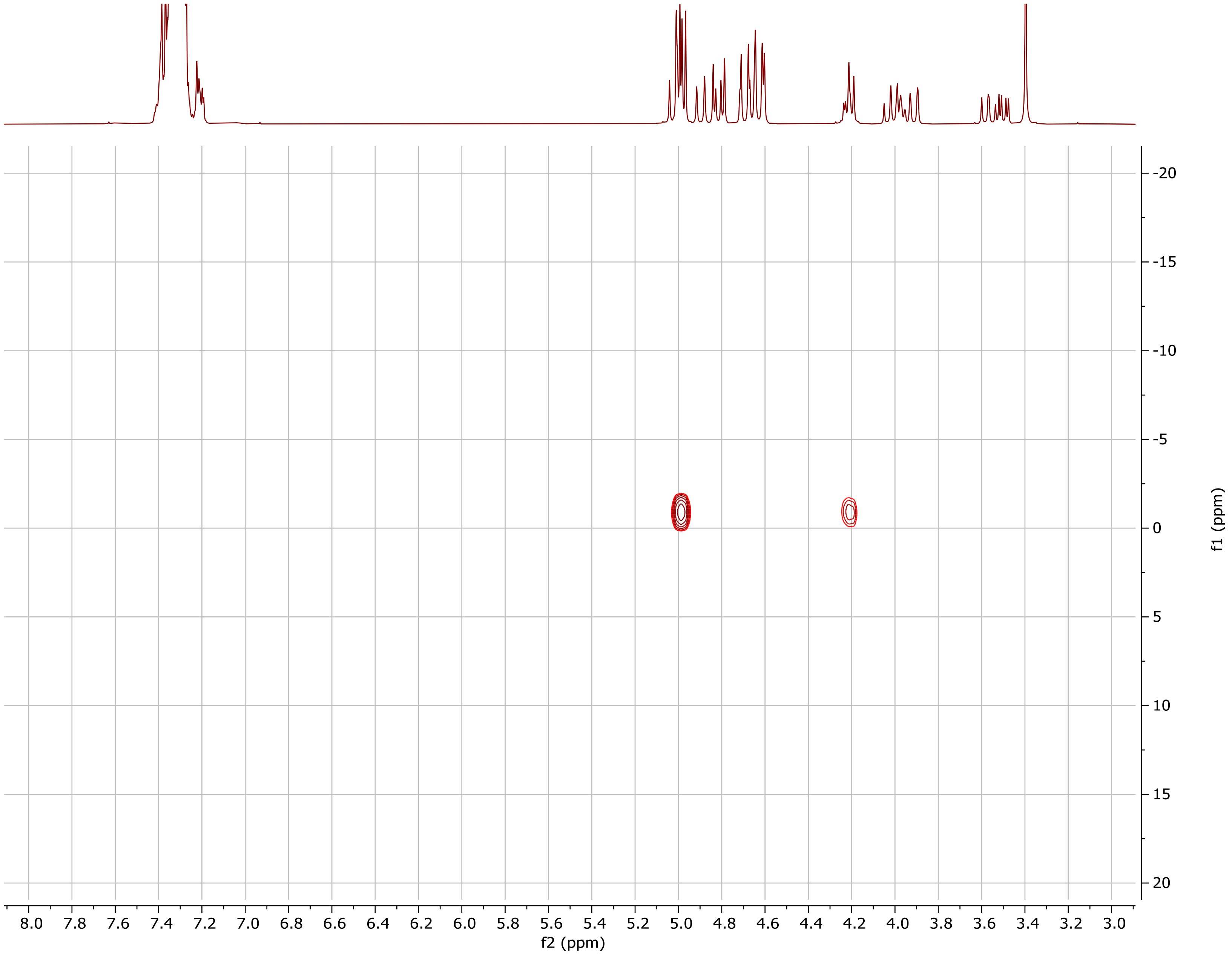

^{1}H NMR spectrum (300 MHz, in D_2O) of 1-*O*-methyl D-*glycero*-α-D-*gluco*-heptopyranoside 7-phosphate (**14**)

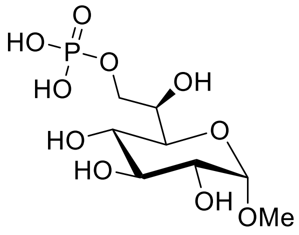

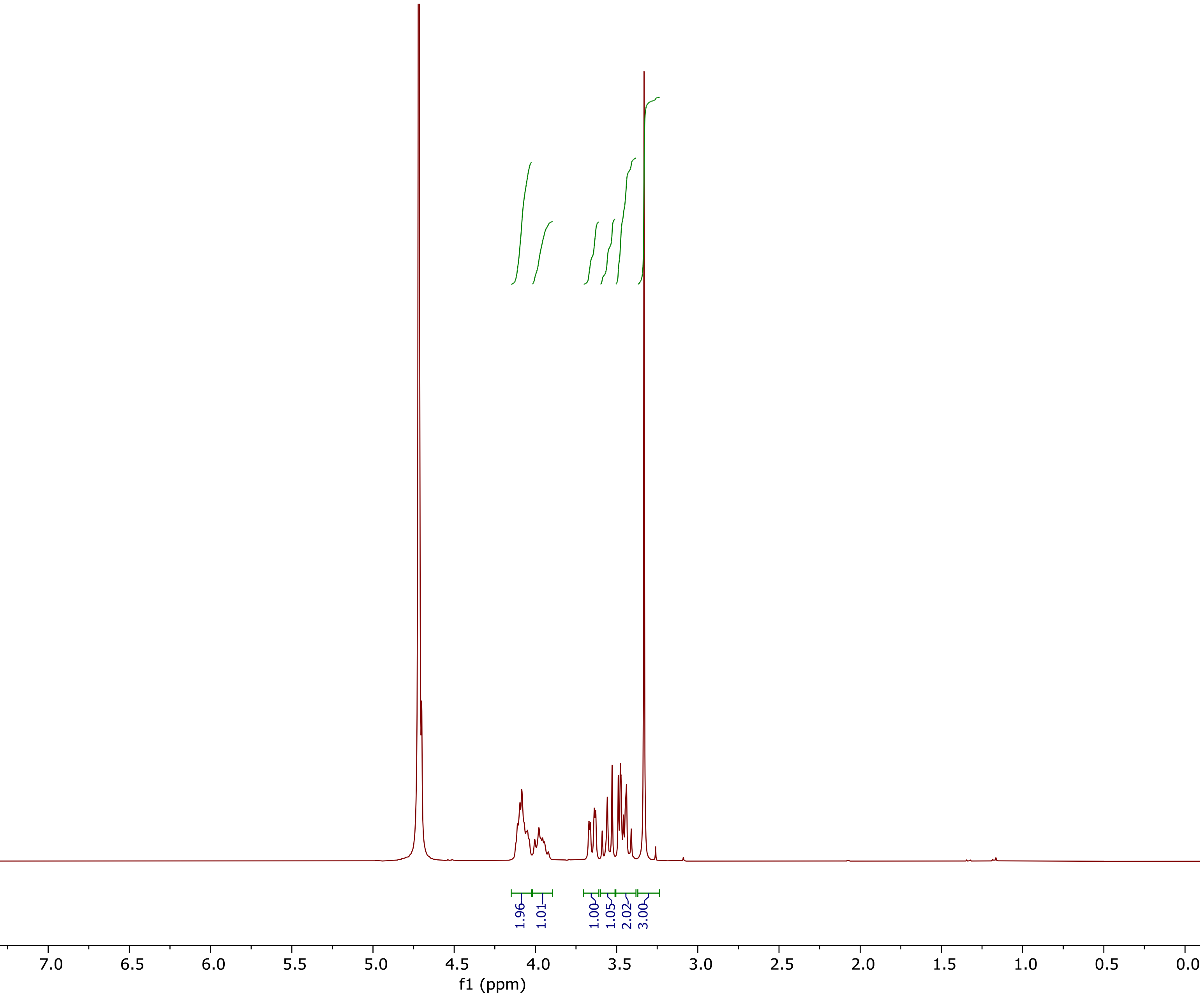

^{1}H DOSY-filtered NMR spectrum (300 MHz, in D_2O) of 1-*O*-methyl D-*glycero*-α-D-*gluco*-heptopyranoside 7-phosphate (**14**)

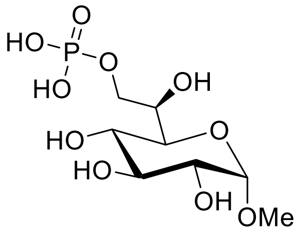

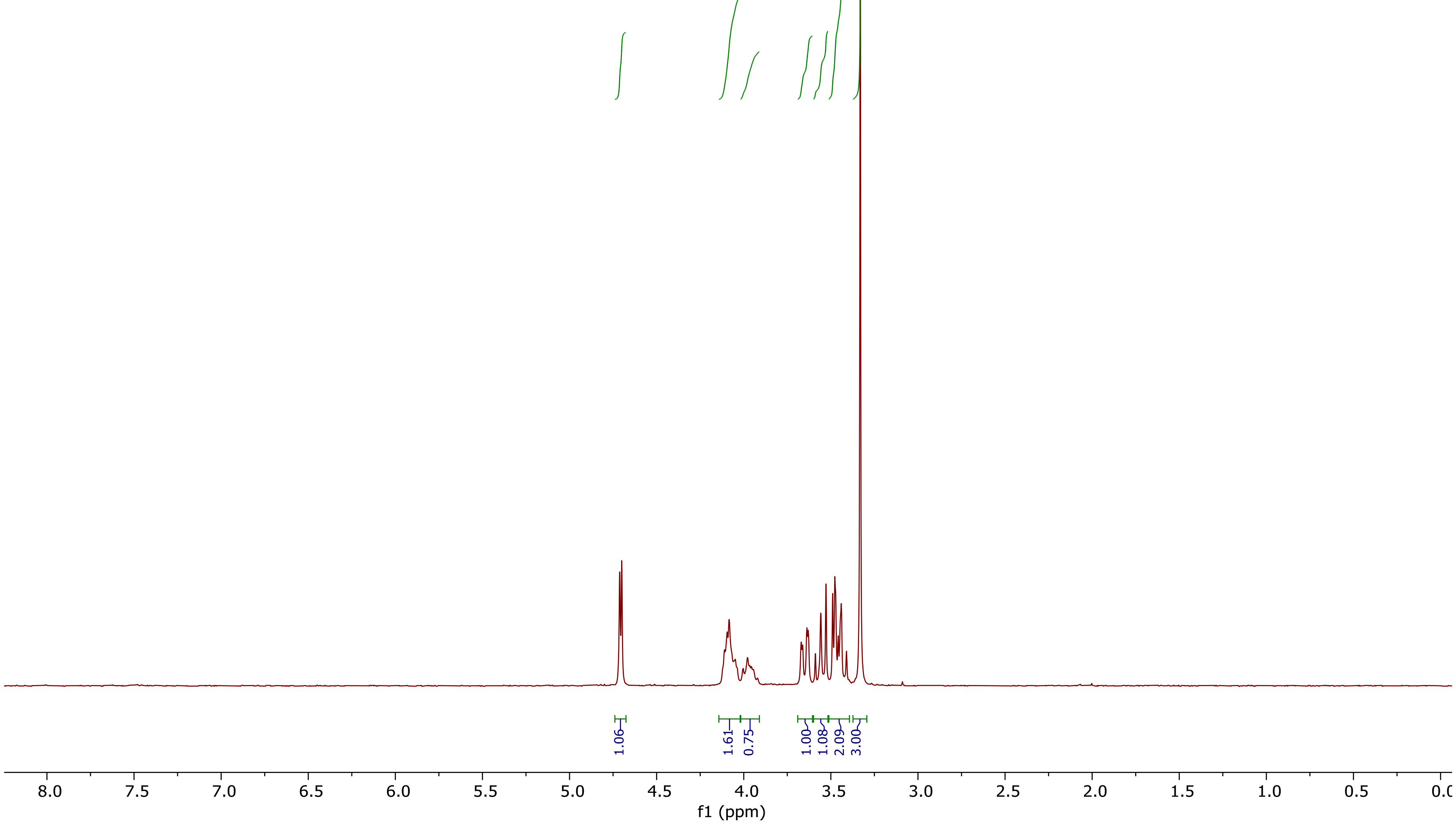

^{13}C NMR spectrum (101 MHz, in D_2O) of 1-*O*-methyl D-*glycero*-α-D-*gluco*-heptopyranoside 7-phosphate (**14**)

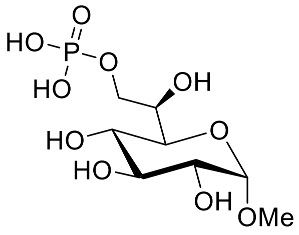

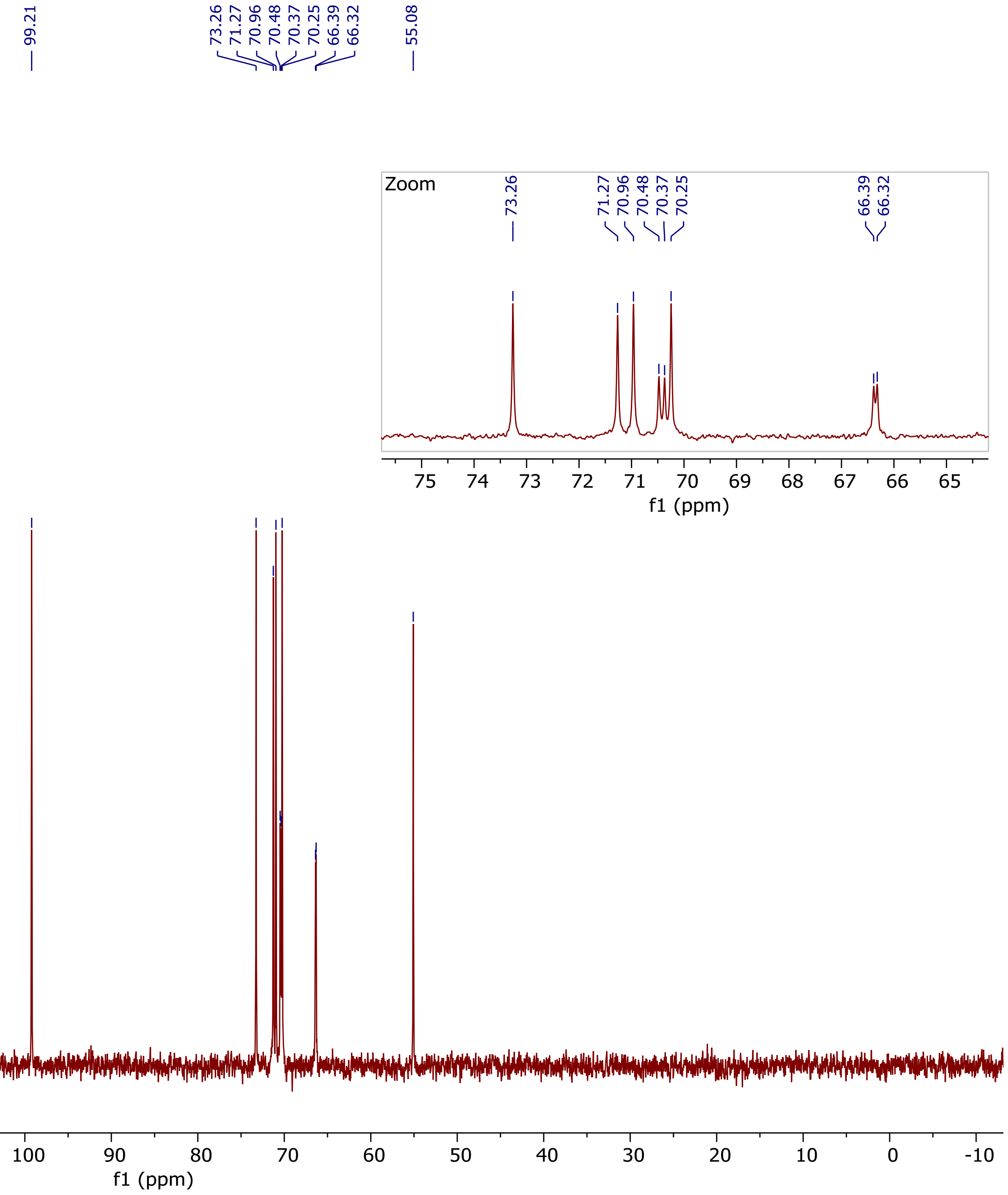

DEPT NMR spectrum (101 MHz, in D_2O) of 1-*O*-methyl D-*glycero*-α-D-*gluco*-heptopyranoside 7-phosphate (**14**)

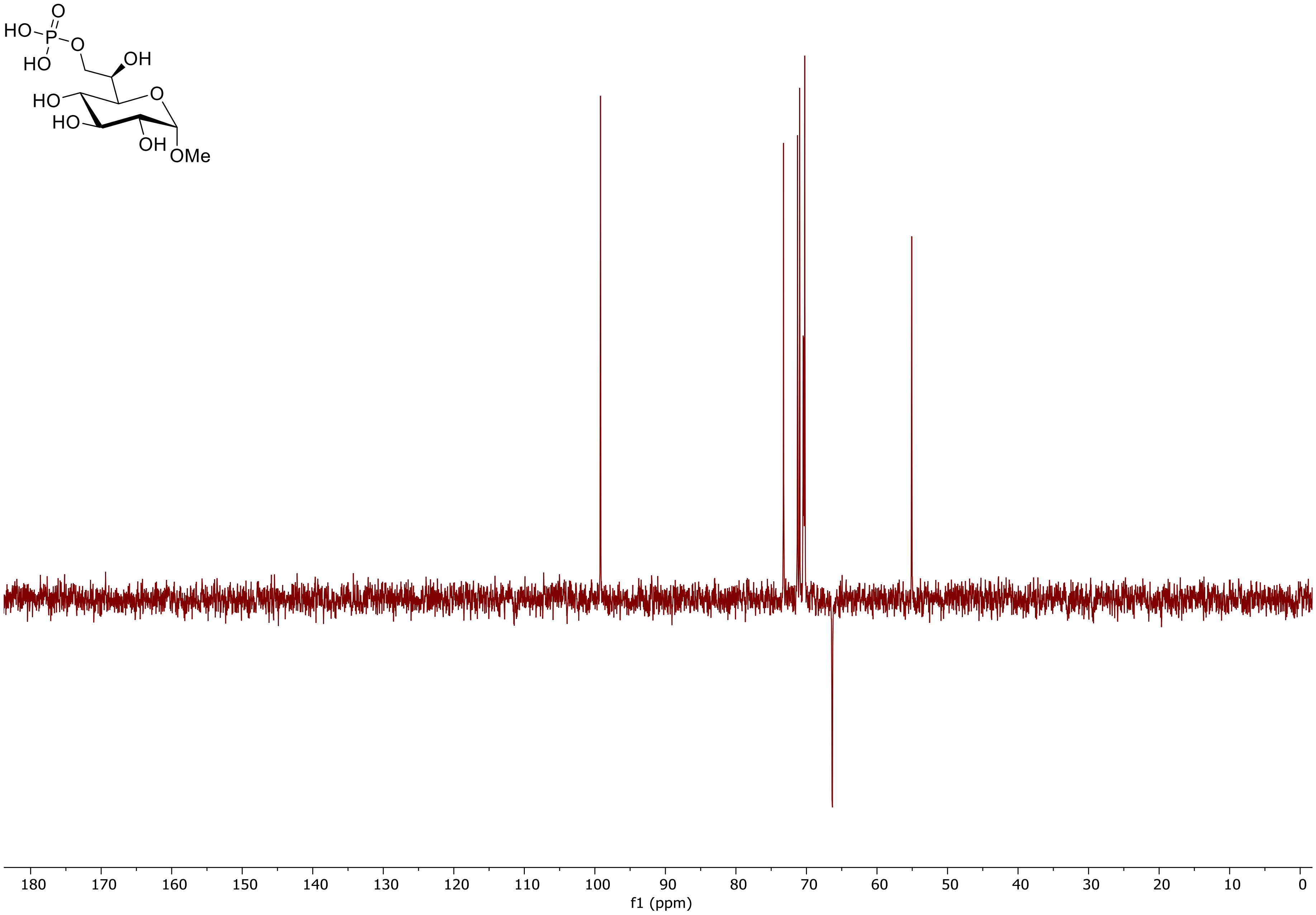

^{31}P NMR spectrum (162 MHz, in D_2O) of 1-*O*-methyl D-*glycero*-α-D-*gluco*-heptopyranoside 7-phosphate (**14**)

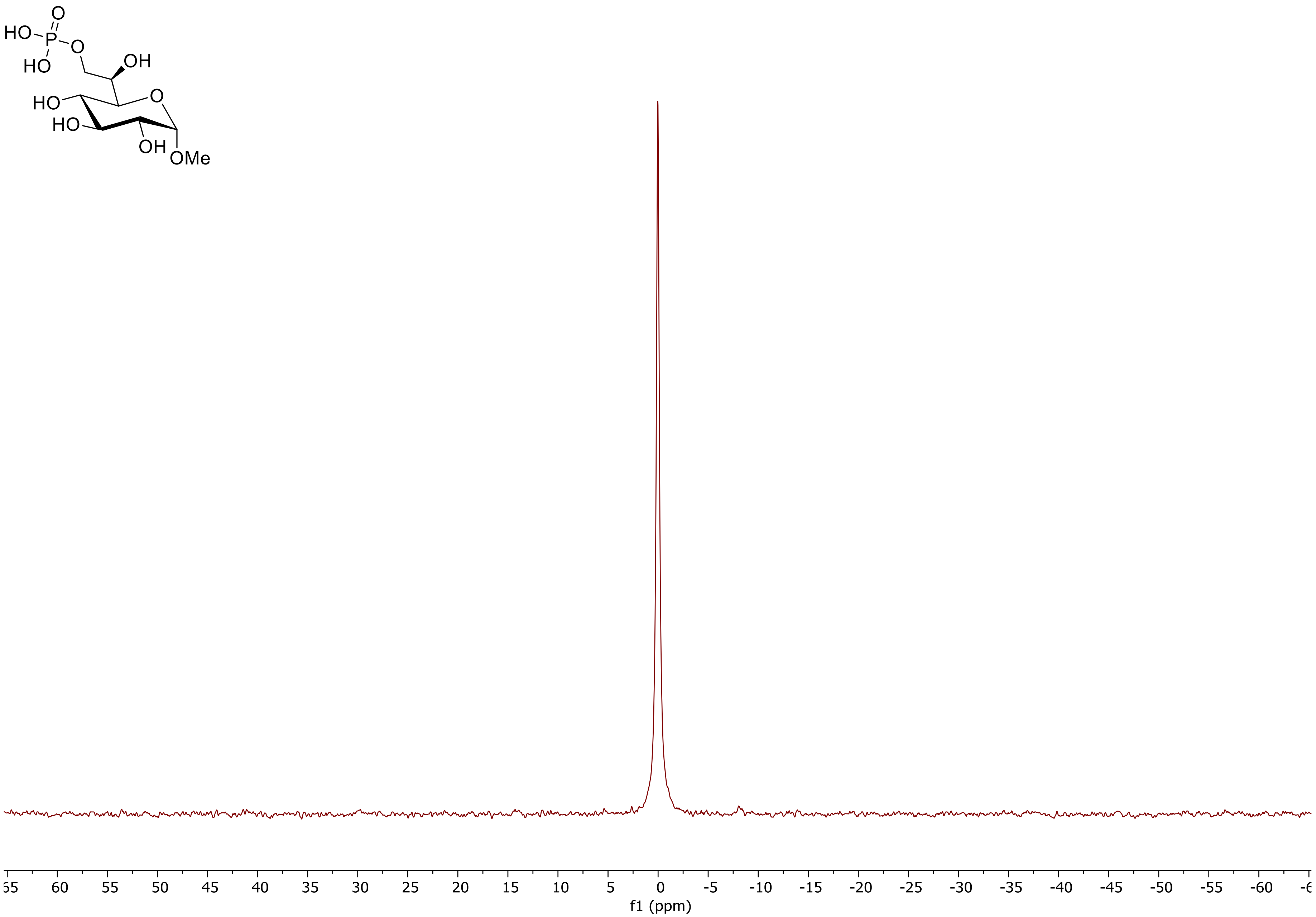

COSY NMR spectrum (400 MHz, in D_2O) of 1-*O*-methyl D-*glycero*-α-D-*gluco*-heptopyranoside 7-phosphate (**14**)

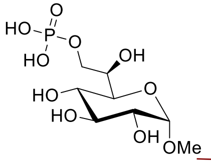

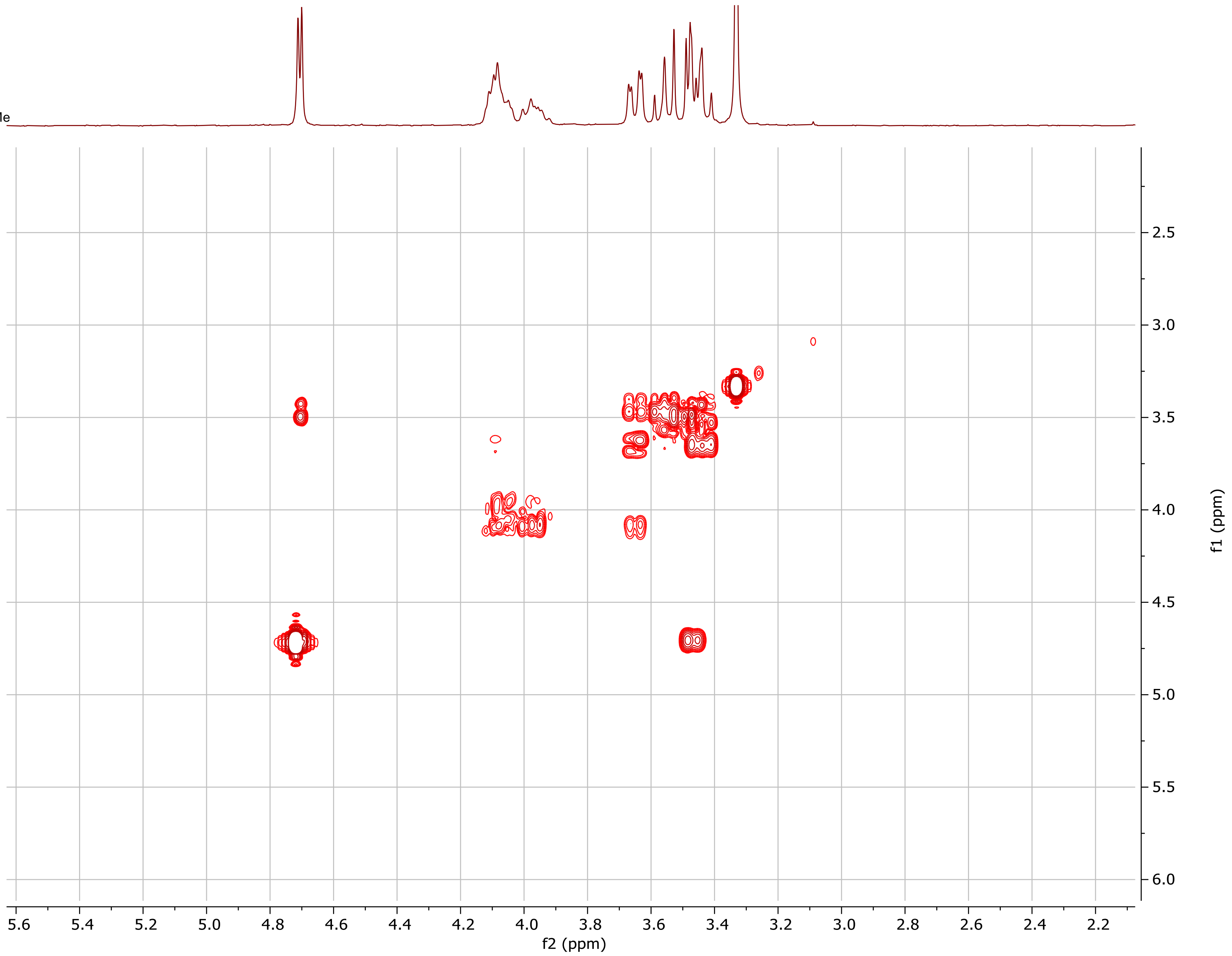

NOESY NMR spectrum (400 MHz, in D_2O) of 1-*O*-methyl D-*glycero*-α-D-*gluco*-heptopyranoside 7-phosphate (**14**)

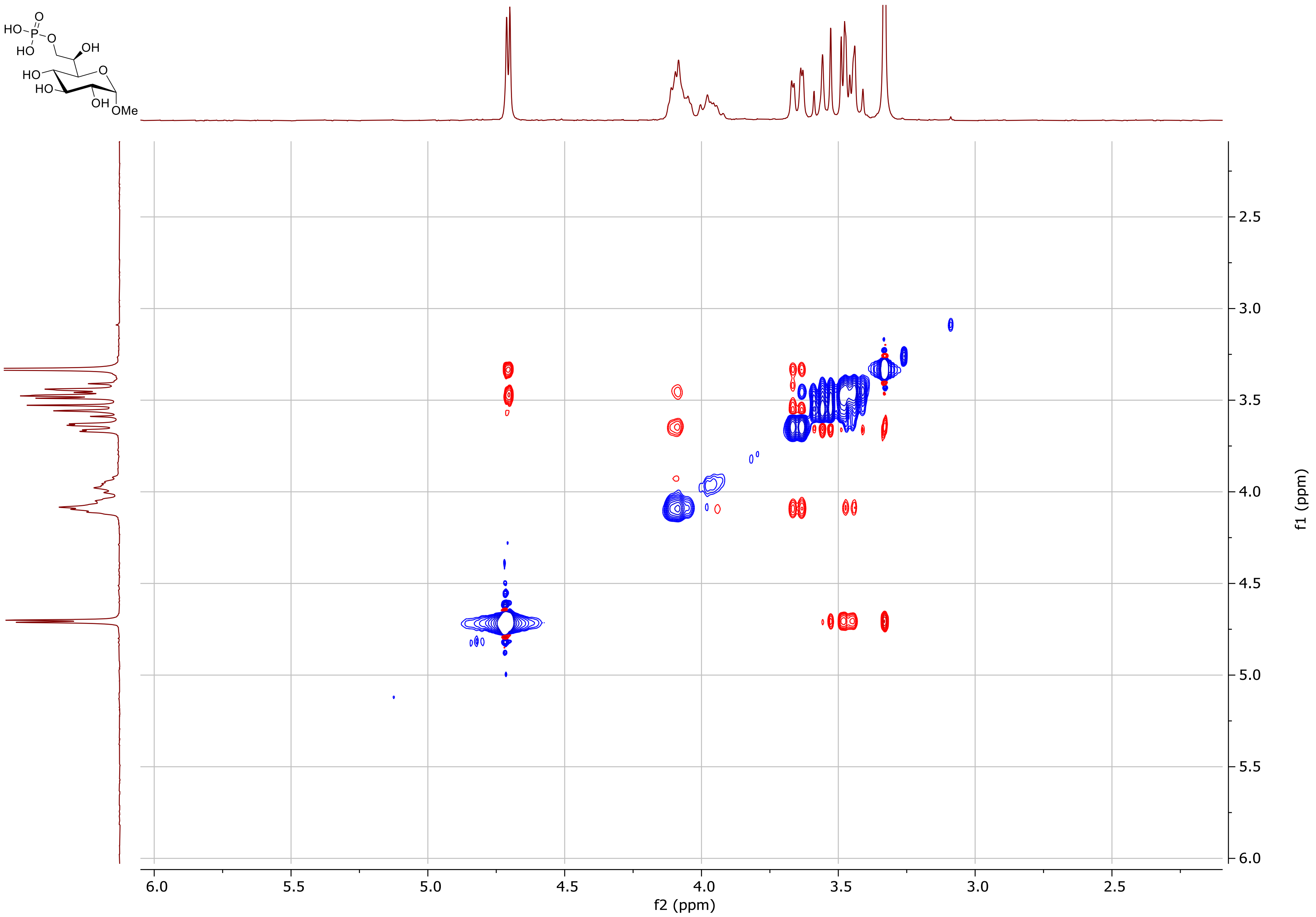

HSQC NMR spectrum (400 & 101 MHz, D_2O) of 1-*O*-methyl D-*glycero*-α-D-*gluco*-heptopyranoside 7-phosphate (**14**)

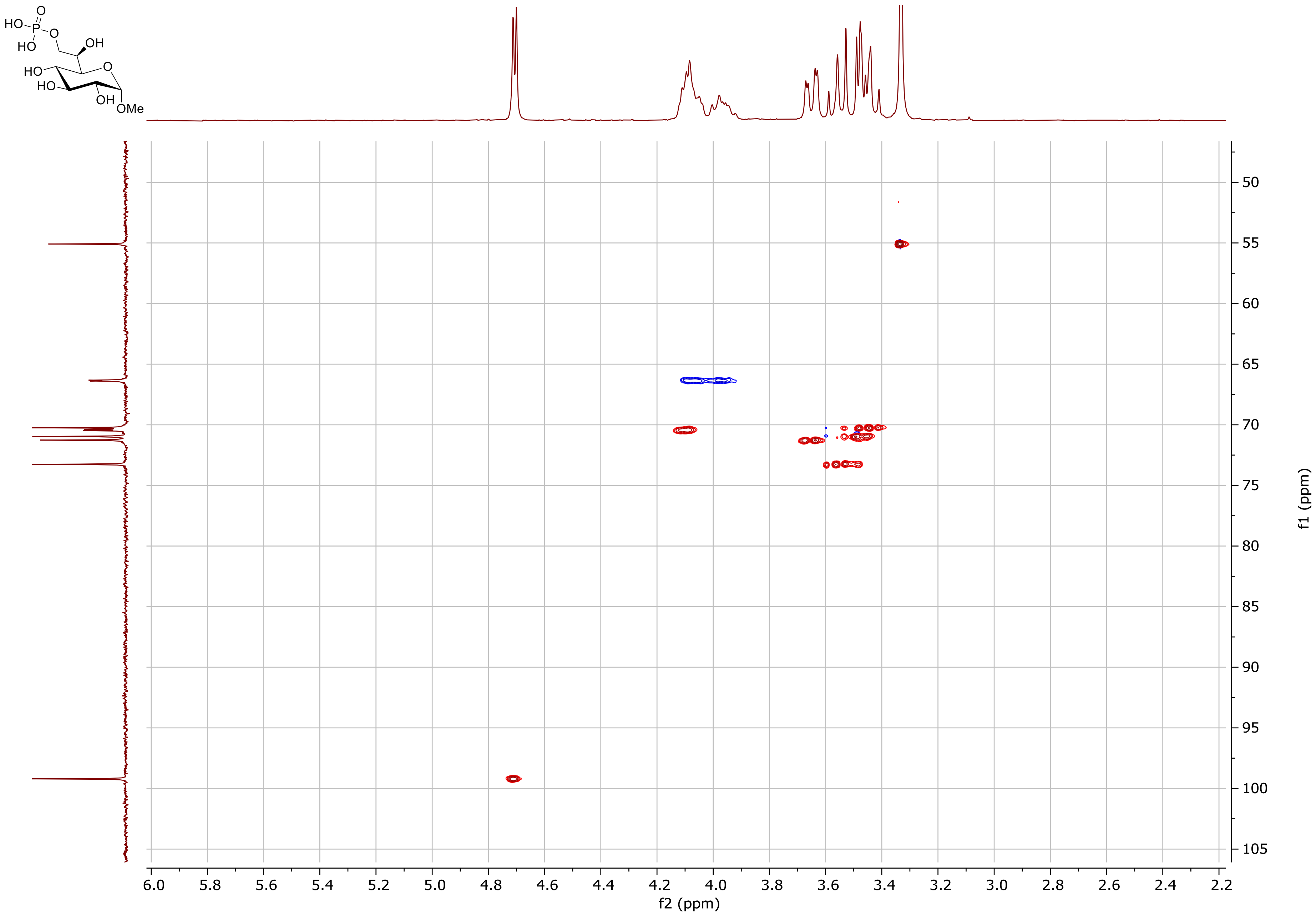

HSQC-TOCSY NMR spectrum (400 & 101 MHz, D_2O) of 1-*O*-methyl D-*glycero*-α-D-*gluco*-heptopyranoside 7-phosphate (**14**)

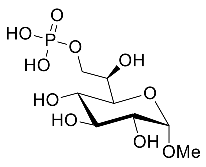

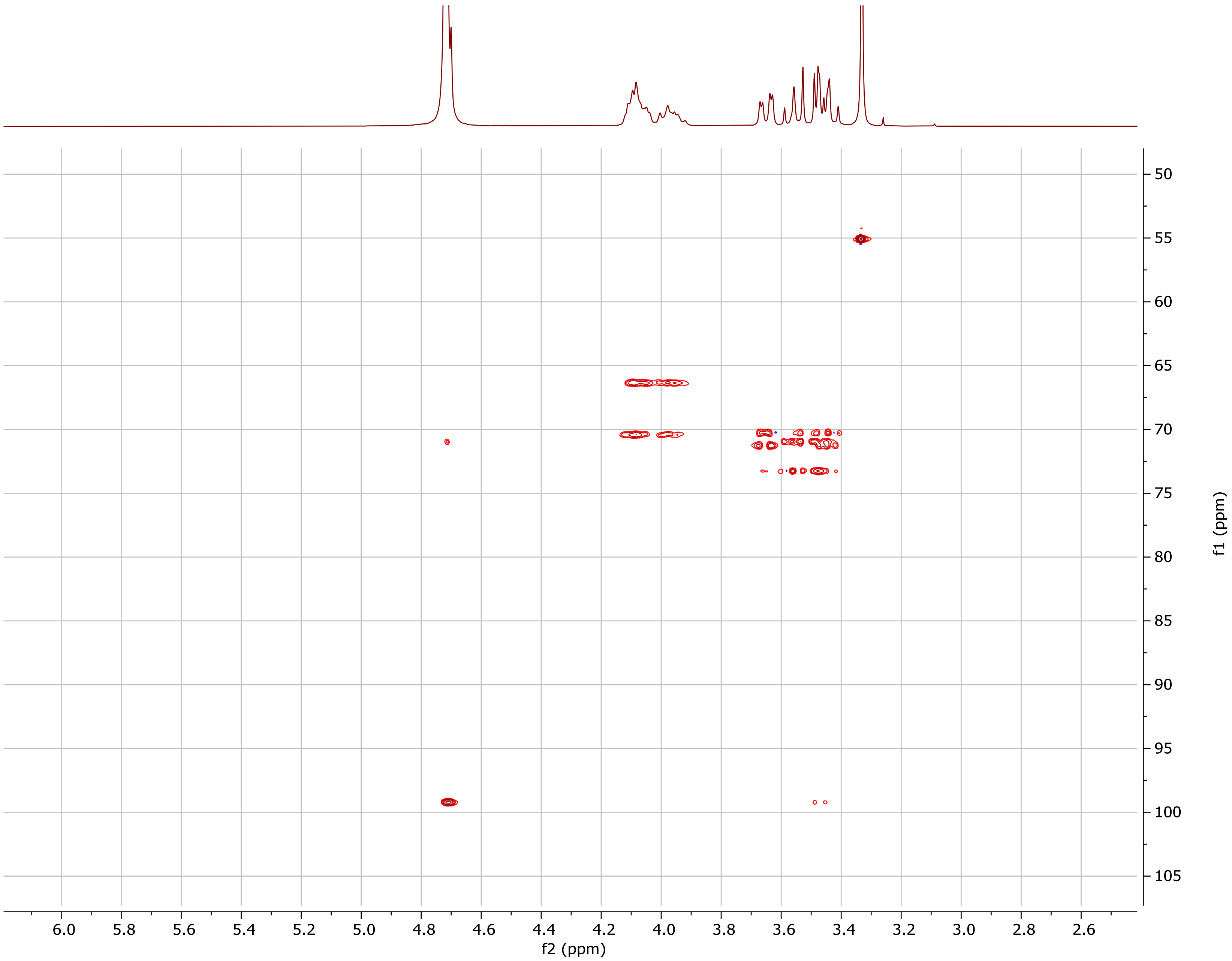

^{1}H,^{31}P-HMBC NMR spectrum (400 & 162 MHz, D_2O) of 1-*O*-methyl D-*glycero*-α-D-*gluco*-heptopyranoside 7-phosphate (**14**)

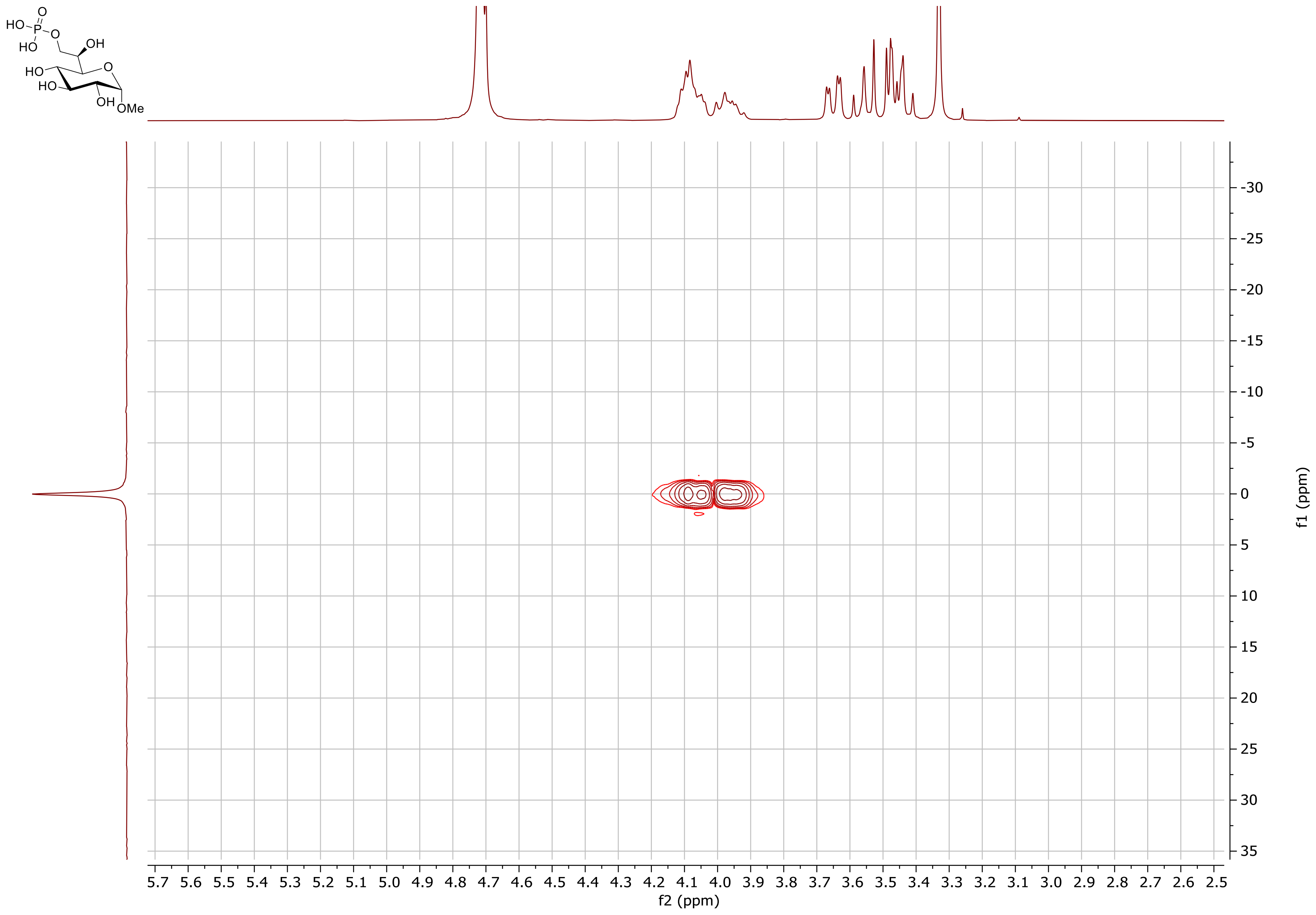

Supplement: Supplementary file 1 [file molecules-27-07534-s001.zip › molecules-1996454-supplementary.pdf]
